# A DNA barcoding method for identifying and quantifying the composition of pollen species collected by European honeybees, *Apis mellifera* (Hymenoptera: Apidae)

**DOI:** 10.1007/s13355-018-0565-9

**Published:** 2018-05-16

**Authors:** Tsunashi Kamo, Yoshinobu Kusumoto, Yoshinori Tokuoka, Satoru Okubo, Hiroshi Hayakawa, Mikio Yoshiyama, Kiyoshi Kimura, Akihiro Konuma

**Affiliations:** 1 Ecosystem Services Assessment Unit, Division of Biodiversity, Institute for Agro-Environmental Sciences, National Agriculture and Food Research Organization, 3-1-3 Kannondai, Tsukuba, Ibaraki 305-8604 Japan; 2 Botanical Society of Tosa, 2452-1, Ananai, Aki, Kochi 784-0032 Japan; 3 Animal Genetics Unit, Division of Animal Breeding and Reproduction Research, Institute of Livestock and Grassland Science, National Agriculture and Food Research Organization, 2 Ikenodai, Tsukuba, Ibaraki 305-0901 Japan; 4 Present Address: Museum of Natural and Environmental History, Shizuoka, 5762 Oya, Suruga-ku, Shizuoka, Shizuoka 422-8017 Japan

**Keywords:** *Apis mellifera*, DNA barcoding, ITS2, Pollen pellet, *trnL*-*trnF*

## Abstract

The European honeybee, *Apis mellifera* L. (Hymenoptera: Apidae), is the most important crop pollinator, and there is an urgent need for a sustained supply of honeybee colonies. Understanding the availability of pollen resources around apiaries throughout the brood-rearing season is crucial to increasing the number of colonies. However, detailed information on the floral resources used by honeybees is limited due to a scarcity of efficient methods for identifying pollen species composition. Therefore, we developed a DNA barcoding method for identifying the species of each pollen pellet and for quantifying the species composition by summing the weights of the pellets for each species. To establish the molecular biological protocol, we analyzed 1008 pellets collected between late July and early September 2016 from five hives placed in a forest/agricultural landscape of Hokkaido, northern Japan. Pollen was classified into 31 plant taxa, of which 29 were identified with satisfactory discrimination (25 species and 4 genera) using *trnL*-*trnF* and ITS2 as DNA barcoding regions together with available floral and phenological information. The remaining two taxa were classified to the species level using other DNA barcoding regions. Of the 1008 pollen pellets tested, 1005 (99.7%) were successfully identified. As an example of the use of this method, we demonstrated the change in species composition of pollen pellets collected each week for 9 weeks from the same hive.

**Electronic supplementary material:**

The online version of this article (10.1007/s13355-018-0565-9) contains supplementary material, which is available to authorized users.

## Introduction

The European honeybee, *Apis mellifera* L. (Hymenoptera: Apidae), has been introduced globally to produce honey and to pollinate crops. Given the current decline of wild pollinators (a problem particularly observed in North America and parts of Europe) and the recent expansion in pollinator-dependent crop cultivation (IPBES 2016), demand for managed pollinators of crops is increasing, and there is an urgent need for a sustained supply of bee colonies to maintain food production (Aizen and Harder 2009; Aizen et al. 2008; Klein et al. 2007).

Honeybees, including managed bees, forage for pollen and nectar as energy sources to feed their own colonies. Foraging workers bring pollen in the form of pollen pellets from flowers to their hives. Pollen is transformed into beebread, the sole food for the brood, which provides them with proteins, amino acids, vitamins, minerals, and lipids (Campos et al. 2008). A decline in the yield of pollen pellets potentially hinders bee colonies’ growth, reproduction, and resilience to stress factors such as pathogens and parasites (Alaux et al. 2010; Allen and Jeffree 1956; Al-Tikrity et al. 1972; Fewell and Winston 1992; Smart et al. 2016; Todd and Reed 1970). Although beekeepers can compensate to some extent for food shortages by providing pollen substitutes such as soybean flour (Alqarni 2006), the availability of floral resources including cultivated and wild flowers around apiaries throughout the brood-rearing seasons is crucial to maintaining and increasing the number of colonies (Naug 2009; Requier et al. 2015). In this context, there are many studies examining how the areas of and distances to mass-cultivated flowers or other land uses, including natural and semi-natural habitats, affect foraging behaviors and colony survival (Danner et al. 2016; Odoux et al. 2012; Requier et al. 2016; Steffan-Dewenter and Kuhn 2003). However, there is still insufficient data on the floral resources used by honeybees to inform appropriate landscape management around apiaries, such as the times and positions for planting additional flower resources relative to colony position (Couvillon et al. 2014; Decourtye et al. 2010). This information could also be useful for recognizing competition for resources between managed honeybees and wild bee species and the disruption of evolutionary plant–pollinator networks (Cane and Tepedino 2017; Goulson 2003).

An important factor hampering the understanding of the floral resources of pollen is that there is no single most efficient method for identifying and quantifying pollen species collected by honeybees. Light microscopic analysis is frequently applied (e.g., Dimou and Thrasyvoulou 2007; Long and Krupke 2016), but that method lacks discriminatory power at lower taxonomic levels (Galimberti et al. 2014; Khansari et al. 2012) and requires expertise and labor. The emerging tool of multiplexed next-generation sequencing is now proposed to be the most powerful method of investigating, as many pollen species are contained in the pollen pellets (Cornman et al. 2015; Kraaijeveld et al. 2015), but there is doubt about the credibility of using this technique for quantification due to possible biases in PCR amplification between plant species (Keller et al. 2015; Richardson et al. 2015; Sickel et al. 2015).

European honeybees produce pollen pellets that are each comprise pollen from (essentially) a single plant species (Almaraz-Abarca et al. 2007; Campos et al. 1997; Decourtye et al. 2011). This characteristic offers the great advantage of enabling us to assume that a pellet is an almost pure source of DNA. Identifying the pollen species in each pellet using DNA information is theoretically possible, and the species composition can be quantified by summing the weights of the pellets for each species. However, to the best of our knowledge, the study reported in the present paper is the first to implement this concept in the analysis of pollen pellets.

The purpose of this study was to first determine which of several DNA barcoding regions could be used to identify pollen species to an acceptable taxonomic level and to develop a DNA barcoding method for identifying the pollen species in each pellet collected from an agricultural landscape in Hokkaido, northern Japan. Based on these data, we then quantified the species composition by summing the weights of the pellets for each species. Finally, using the method developed here, we demonstrated the seasonal changes in the species composition of pollen resources collected from late July to early September 2016 from a honeybee hive in Hokkaido.

## Materials and methods

### Pollen collection and DNA extraction

**Fig. 1:**
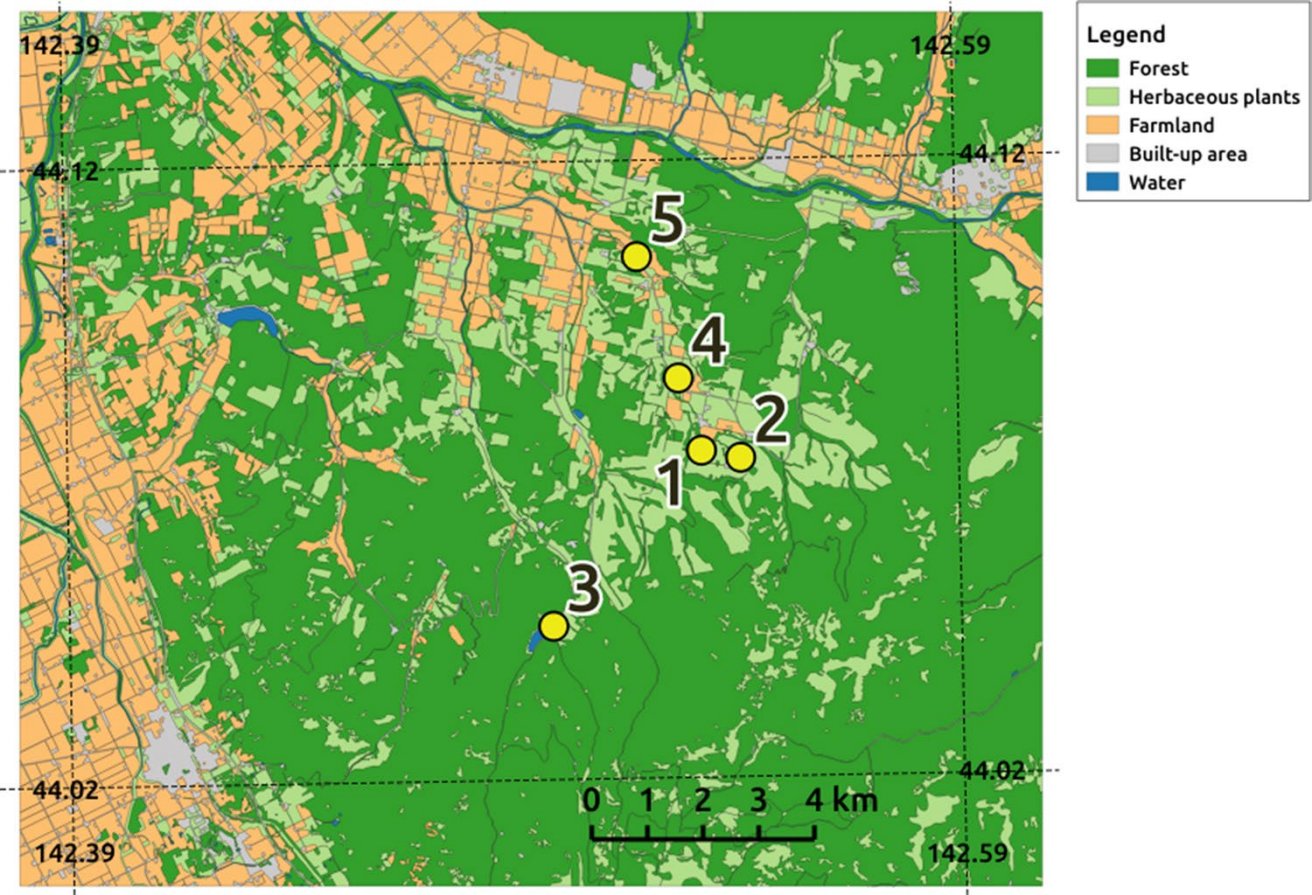
Locations of honeybee hives (sites 1–5) set up on 19 July 2016 in Shibetsu City, northern Hokkaido. In this landscape, herbaceous plants were mainly grasses for livestock. Honeybee pollen pellets were collected weekly from 21 July to 14 September

Honeybee pollen pellets were sampled in Hokkaido, where many migratory beekeepers in Japan spend the summer with their bee colonies to collect honey and raise their colonies in conditions that are cooler than in other parts of Japan. We set up five experimental apiaries in an agricultural area of Shibetsu City on 19 July 2016, sited with consideration to differences in the surrounding land uses (Fig. 1). Sites 1 and 2 were located within grasslands (labeled “herbaceous plants” in Fig. 1); site 3 was also in grassland, but close to forests; and sites 4 and 5 were placed along a gradient from grasslands to farmlands. Each apiary comprised seven queen-right hives, two of which were equipped with pollen traps to collect pollen pellets (for details, see Galimberti et al. 2014). We collected pollen pellets every week for 9 weeks from late July to early September in 2016. Pollen collection was performed in the morning for about 3 h starting at around 09:00 hours. Pollen pellets collected from each hive were put into a sampling tube, packed on ice in a cooler bag, brought back to a laboratory in Tsukuba, Ibaraki, weighed, and then stored at − 20 °C until analysis.

From each apiary, we chose the hive that had constantly higher pollen yields than the others throughout the monitoring period for analyses. To cover as many pollen species as possible with the most efficient use of time and labor, we used all pollen pellets collected for 9 weeks from the chosen hive at site 5—which was nearest to farmlands—to take into account seasonal changes in floral resources in a heterogeneous agricultural landscape, and we used three collections (obtained on 26 July, 10 August, and 25 August) from the chosen hive in each apiary at sites 1–4 to consider varied floral resources in different land uses ranging from forests to grasslands. From each collection, a sample of 48 randomly chosen pollen pellets were used for species identification. We decided on this number of sampled pollen pellets because a set of 48 tubes was a convenient number to handle in the molecular biological procedures. We analyzed a total of 1008 pollen pellets (21 samples), which were derived from four hives for 3 weeks (12 samples) and from one hive for 9 weeks (9 samples; see Table S1 in the Electronic supplementary material, ESM).

DNA was extracted using a DNeasy Plant Mini Kit (Qiagen, Valencia, CA, USA). Each pollen pellet was weighed before DNA extraction. A microtube containing a pollen pellet, two stainless steel beads (5 mm diameter), and 400 µL of lysis buffer was vortexed for 10 s. The following procedures were conducted according to the manufacturer’s protocol, except for elution from the DNeasy spin column in the final step, where we used 1 × 100 µL of elution buffer.

### Amplification and sequencing of barcoding regions

DNA barcoding analysis was conducted using the *trnL* intron and *trnL*-*trnF* intergenic spacer of chloroplast DNA (*trnL*-*trnF*) and the internal transcribed spacer 2 region of nuclear ribosomal DNA (ITS2). The primer combination used for PCR amplification and sequencing of *trnL*-*trnF* was trnL-c (forward): 5′-CGAAATCGGTAGACGCTACG-3′ and trnF-f (reverse): 5′-ATTTGAACTGGTGACACGAG-3′ (Taberlet et al. 1991). The primer combination used for ITS2 was ITS3 (forward): 5′-GCATCGATGAAGAACGCAG-3′ and ITS4 (reverse): 5′-TCCTCCGCTTATTGATATGC-3′ (White et al. 1990). The amplification reaction contained 0.08 µL of Ex Taq polymerase (TaKaRa Bio, Shiga, Japan; 5 U/µL), 1.6 µL of Ex Taq buffer (Mg^2+^ free), 1.3 µL of MgCl_2_ (25 mM), 1.3 µL of dNTP mixture (2.5 mM each), 0.5 µL of DNA template, 0.5 µL each of forward and reverse primers (10 µM), and purified water up to 16.6 µL. PCR cycles consisted of an initial denaturation step for 4 min at 94 °C, 35 cycles of denaturation (30 s at 94 °C), annealing (40 s at 52 °C), extension (1 min at 72 °C), and a final extension at 72 °C for 10 min. The PCR products obtained were checked by electrophoresis on 2% (w/v) agarose gel, treated with ExoSAP-IT (USB Corp., Cleveland, OH, USA), and directly sequenced in both directions in an ABI 3130XL genetic analyzer (Applied Biosystems, Foster City, CA, USA) with a Big Dye Terminator Cycle Sequencing Ready Reaction Kit (Applied Biosystems) using the same primers as were used for PCR.

Other DNA barcoding regions (*psbA*-*trnH* and *matK*) were used for further pollen species identification if the results for *trnL*-*trnF* and ITS2 were not sufficient. The primer combination used for *psbA*-*trnH* was psbAF (forward): 5′-GTTATGCATGAACGTAATGCTC-3′ and trnHR (reverse): 5′-CGCGCATGGTGGATTCACAAATC-3′ (Sang et al. 1997). PCR conditions were identical to those for *trnL*-*trnF* and ITS2. The primer combination for *matK* was designed for a candidate species (*Monochoria korsakowii* Regel et Maack) implied by the result of *psbA*-*trnH*: 116F (forward) 5′-AGTGCAGTACTTGTGAAACGT-3′ and 1183R (reverse) 5′-ACAAATCGGTCCAAATGGGC-3′. The following steps were used for the PCR amplification: denaturation at 95 °C for 30 s, annealing and extension at 68 °C for 1 min, decreasing by 1 °C in each cycle for 10 cycles, followed by 25 cycles of 95 °C for 30 s, 58 °C for 1 min, and a final extension at 72 °C for 4 min. The PCR products obtained from the DNA templates were processed in the same manner as for other taxa.

### Molecular identification of barcoding regions

Molecular identification of the amplified sequences was performed by reconstructing a phylogenetic tree for each sequence along with species showing high similarity that were retrieved from the GenBank/NCBI database (https://blast.ncbi.nlm.nih.gov/Blast.cgi) using the Basic Local Alignment Search Tool search (Altschul et al. 1990). The amplified sequences and similar sequences collected from the database were aligned using ClustalW (Thompson et al. 1994), included within MEGA7 (Kumar et al. 2016). Phylogenetic relationships were analyzed using maximum likelihood based on the Tamura–Nei model (Tamura and Nei 1993). The maximum likelihood analyses were performed with MEGA7. By considering floral and phenological information (Ohashi et al. 2015, 2016a, b, 2017a, b; Shimizu 2003), most of the sequences could be identified at the species level (Tables S2, S3 in the ESM). Identification of the remaining sequences terminated at the genus level. The results for each DNA barcoding region were then unified; in cases where identification by each DNA barcoding region for a single taxon terminated at different levels, the more highly resolved identification was adopted. The sequences were deposited in the DNA Data Bank of Japan (DDBJ accession numbers LC375689 to LC375713 for *trnL*-*trnF*, LC375714 to LC375737 for ITS2, LC375738 for *psbA*-*trnH*, and LC375739 for *matK*). Nomenclature followed that of the YList (http://ylist.info), an online service of Japanese plant names, including a nomenclature index.

### Application of the method to explore seasonal changes in pollen resources

Using the abovementioned methods, we demonstrated the seasonal change in the composition of pollen species of one hive at site 5 throughout 9 weeks. After identifying the species for each pellet, the cumulative pellet weight of each species was calculated, and the proportional change in species composition was plotted.

## Results

### Molecular identification of honeybee pollen pellets

Based on the sequencing analyses, 1008 pollen pellets were classified into 31 taxa. They were identified at the species or genus levels using DNA barcoding region(s) together with floral and phenological information. Although 25 of 31 taxa (80.6%) could be identified using *trnL*-*trnF* and 24 (77.4%) could be identified using ITS2 (Table 1), we were able to identify 29 taxa (93.5%) using the combination of these two DNA barcoding regions. The two unidentifiable taxa were re-examined using *psbA*-*trnH*. One of them was identified as *Commelina communis* L.; the other likely belonged to the family Pontederiaceae. Two native species, *M. korsakowii* and *Monochoria vaginalis* (Burm. f.) C. Presl ex Kunth, and two alien species, *Eichhornia crassipes* (Mart.) Solms and *Heteranthera limosa* (Sw.) Willd., are the four known species of Pontederiaceae in Japan. Because *M. korsakowii* was the only species among the four that was distributed within the study area (Ohashi et al. 2015; Shimizu 2003), we designed a pair of primers for *matK* that were suitable for this species and confirmed this identification.

**Table 1:** Molecular identification of honeybee pollen pellets collected in Hokkaido, northern Japan

No.	Identified plant^a^	Identification using each DNA barcoding region	
		*trnL*-*trnF*	ITS2
1	*Actinidia polygama* (Siebold et Zucc.) Planch. ex Maxim.	*Actinidia polygama*	*Actinidia polygama*
2	*Angelica* sp.	*Angelica* sp.	*Angelica* sp.
3	*Artemisia montana* (Nakai) Pamp.	*Artemisia* sp.	*Artemisia montana*
4	*Asparagus officinalis* L.	*Asparagus officinalis*	*Asparagus officinalis*
5	*Chelidonium majus* L.	*Chelidonium majus*	*Chelidonium majus*
6	*Chenopodium album* L.	*Chenopodium album*	*Chenopodium* sp.
7	*Cirsium vulgare* (Savi) Ten.	*Cirsium vulgare*	*Cirsium vulgare*
8	*Commelina communis* L.^b^	–	–
9	*Cucurbita maxima* Duchesne ex Lam.	–	*Cucurbita maxima*
10	*Fagopyrum esculentum* Moench	*Fagopyrum esculentum*	–
11	*Fallopia sachalinensis* (F. Schmidt) Ronse Decr.	*Fallopia sachalinensis*	–
12	*Filipendula camtschatica* (Pall.) Maxim.	*Filipendula* sp.	*Filipendula camtschatica*
13	*Hydrangea paniculata* Siebold	*Hydrangea paniculata*	*Hydrangea paniculata*
14	*Hydrangea petiolaris* Siebold et Zucc.	*Hydrangea petiolaris*	–
15	*Hypochaeris radicata* L.	*Hypochaeris radicata*	*Hypochaeris radicata*
16	*Kalopanax septemlobus* (Thunb.) Koidz.	*Kalopanax septemlobus*	*Kalopanax septemlobus*
17	*Monochoria korsakowii* Regel et Maack^c^	–	–
18	*Oryza sativa* L.	*Oryza sativa*	–
19	*Parasenecio* sp.	*Parasenecio* sp.	Senecioneae
20	*Plantago lanceolata* L.	*Plantago* sp.	*Plantago lanceolata*
21	*Plantago* sp.	–	*Plantago* sp.
22	*Rudbeckia laciniata* L.^d^	*Rudbeckia hirta*	*Rudbeckia laciniata*
23	*Sagittaria trifolia* L.	–	*Sagittaria trifolia*
24	*Sinapis alba* L.	–	*Sinapis alba*
25	*Solanum nigrum* L.	*Solanum nigrum*	*Solanum nigrum*
26	*Solidago gigantea* Aiton	*Solidago gigantea*	*Solidago gigantea*
27	*Solidago virgaurea* L.	*Solidago* sp.	*Solidago virgaurea*
28	*Tilia* sp.	*Tilia* sp.	*Tilia* sp.
29	*Trifolium pratense* L.	*Trifolium* sp.	*Trifolium pratense*
30	*Trifolium repens* L.	*Trifolium repens*	*Trifolium repens*
31	*Zea mays* L.	*Zea mays*	–

^a^In cases where identification using each DNA barcoding region terminated at different levels (nos. 3, 8, 12, 19, 20, 27, and 29), the more highly resolved identification was adopted

^b,c^Identifiable using other DNA barcoding regions (^b^*psbA*-*trnH* or ^c^*matK*). See Figs. S3, S4 in the ESM for the molecular phylogenetic trees

^d^Because the *trnL*-*trnF* sequence of *Rudbeckia laciniata* was not registered in the database, identification using ITS2 was adopted. ITS2 molecular identification confirmed that *Rudbeckia hirta* was located in a different monophyletic group from that of pollen pellet no. 22 (Fig. S2 in the ESM)

Of the 31 taxa, 25 were classified to species level by referring to the phylogenetic trees in *trnL*-*trnF* and ITS2, together with the available information about the flora and the flowering seasons in Hokkaido (Tables 1, S2, S3; Figs. S1–S4 in the ESM). The four remaining taxa were classified to the genus level: *Angelica* sp., *Parasenecio* sp., *Plantago* sp., and *Tilia* sp. Because 29 of the 31 taxa were classifiable using *trnL*-*trnF* and ITS2, this combination proved to be practical for identifying the honeybee pollen pellets collected in Hokkaido. The validity of the species identification was confirmed to be reasonable by comparison with the results of a vegetation survey (Table S4 in the ESM).

The combined use of *trnL*-*trnF* and ITS2, and sometimes also *psbA*-*trnH* and *matK*, as DNA barcoding regions resulted in the successful classification of 1005 of the 1008 (99.7%) pollen pellets. Of the three pellets that could not be classified, two were mixed pellets, which obviously consisted of two or more pollen species based on the color of the pellets, and, for no plausible reason, one pellet showed no PCR amplification for any of the barcoding regions. Because these three pollen pellets accounted for a very small percentage of all pellets, we conducted no further analysis on them.

### Seasonal change in pollen species composition

**Fig. 2a–i:**
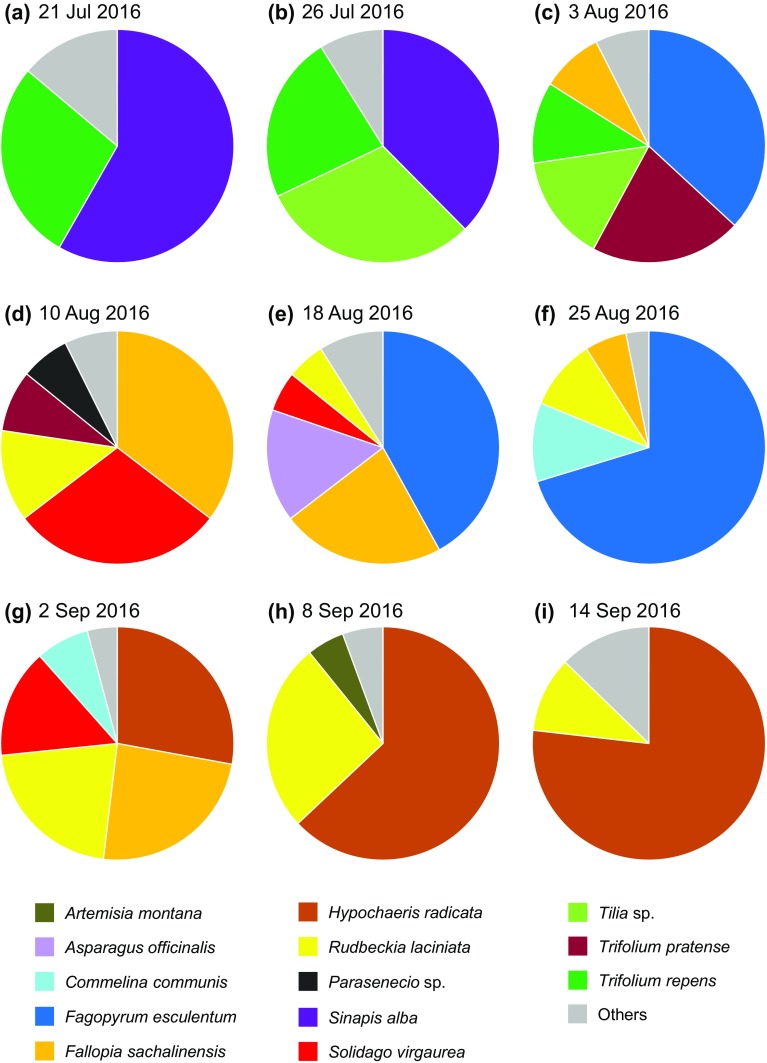
Species compositions (w/w) of honeybee pollen pellets collected at Shibetsu City, northern Hokkaido. Proportions of minor species (< 5%), shown as “others” in the plots, were as follows: **a** *Tilia* sp. (3.8%), *Actinidia polygama* (3.0%), *Hydrangea petiolaris* (2.5%), *Hydrangea paniculata* (2.1%), *Hypochaeris radicata* (1.7%), *Parasenecio* sp. (0.8%); **b** *H. petiolaris* (2.8%), *Parasenecio* sp. (2.7%), *H. paniculata* (1.9%), *H. radicata* (1.5%); **c** *Rudbeckia laciniata* (3.6%), *Parasenecio* sp. (2.6%), *Sinapis alba* (1.2%); **d** *Fagopyrum esculentum* (3.9%), *H. radicata* (3.5%); **e** *Trifolium pratense* (2.2%), *Parasenecio* sp. (1.6%), *Trifolium repens* (1.4%), *Chenopodium album* (1.3%), *Commelina communis* (1.2%), unidentified (1.3%); **f** *T. pratense* (1.6%), *Solidago gigantea* (1.6%); **g** *S. gigantea* (4.2%); **h** *Solidago virgaurea* (3.8%), *C. communis* (1.9%); **i** *Artemisia montana* (4.2%), *S. virgaurea* (3.5%), *Solanum nigrum* (2.3%), *T. pratense* (1.6%), *T. repens* (1.1%)

Using the abovementioned method, we calculated the proportional changes in pollen species composition by weight in one hive at site 5 throughout nine consecutive weeks from late June to mid-September 2016 (Fig. 2). In the pollen pellets sampled in July, *Sinapis alba* L., *Tilia* sp., and *Trifolium repens* L. constituted the major species. *Actinidia polygama* (Siebold et Zucc.) Planch. ex Maxim., *Hydrangea petiolaris* Siebold et Zucc., and *Hydrangea paniculata* Siebold were also detected as minor species, indicating that honeybees utilized, to some extent, the flowers of trees and shrubs until early August in the area studied. In the samples from August, *Fagopyrum esculentum* Moench was the most abundant species, accompanied by the pollen of weeds such as *Fallopia sachalinensis* (F. Schmidt) Ronse Decr., *Rudbeckia laciniata* L., *Solidago virgaurea* L., and *Trifolium pratense* L. In the samples from September, *Hypochaeris radicata* L. was the dominant species, followed by *R. laciniata*, which was continuously observed from August. It was thus conceivable that the direction in which honeybees flew to forage for flower resources changed dramatically from mountainous areas to farming areas in early August.

## Discussion

The present study revealed that a combination of several DNA barcoding regions and available floral and phenological information could be used to identify pollen pellets to satisfactory taxonomic levels. We tested *trnL*-*trnF* and ITS2 as DNA barcoding regions, because the former is variable enough to allow the identification of most plants to the genus level (Kraaijeveld et al. 2015) and the latter was used successfully to identify 92.7% of 6600 plant samples (Buchheim et al. 2011; Chen et al. 2010). These regions resulted in good amplification in our study, and good identification was possible due to abundant information in the reference database; nevertheless, other DNA barcoding regions, such as ITS1, *rbcL*, *psbA*-*trnH*, and *matK*, would also be promising for use as standard primers. For example, *psbA*-*trnH* was used as an alternative to *trnL*-*trnF* while we were establishing our protocol because it showed resolution in identification that was as high as that of *trnL*-*trnF* (data not shown). The only drawback of *psbA*-*trnH* in our trial was the relatively poor quality of the chromatograms, although there may still be room for improvement by modifying the primers or the PCR conditions used. Using multiple DNA barcoding regions is desirable as it enables high discriminatory power and accurate identification through double-checking (Galimberti et al. 2014), but it appears that the choice of DNA barcoding regions from among standardized regions is flexible and can be modified depending on the level of taxonomic variation.

Honeybees reportedly form each pollen pellet from a single plant species (Almaraz-Abarca et al. 2007; Campos et al. 1997; Decourtye et al. 2011), and our results confirmed this behavior; only two pellets of the 1008 tested were mixed pellets. Therefore, our protocol is feasible without additional consideration of this issue. However, DNA meta-barcoding should also be considered when dealing with other bee species or analyzing a set of pollen pellets collectively. That approach has been the focus of many recent studies, since it has the potential to be more labor-saving and it targets a greater number of pollen species (Cornman et al. 2015; Galimberti et al. 2014; Keller et al. 2015; Long and Krupke 2016; Richardson et al. 2015). Multiplexed next-generation sequencing is a key technique, as enables researchers to obtain a list of species contained in a heterogeneous mixture of pollen pellets. However, we need to bear in mind an intrinsic shortcoming of this procedure. As noted in several reports, data obtained from meta-barcoding is less reliable in terms of quantification than other techniques such as light microscopy (Richardson et al. 2015; Sickel et al. 2015). This weakness in quantification derives from the following variations between plant species: the number of ribosomal DNA cassette copies, genome copy number, DNA extraction efficiency, and primer annealing efficiency (Sickel et al. 2015). In an unfortunate case, we could miss the most abundant pollen species in a sample due to its poor amplification in the first PCR. Worse still, we would not recognize that we had missed it. An alternative scheme to compensate for this defect is light microscopic analysis, but, even though this would ensure that the most abundant pollen species is not overlooked, it does require expert knowledge. In addition, even with the skill of experienced specialists, pollen is often identified only to the family level or higher (Galimberti et al. 2014; Khansari et al. 2012).

The units used for the three methods mentioned above are different. Our newly developed method, the unit of which is fresh weight, is practical for studying bee nutrition. It is not the number but the total weight of pollen grains that is important for the growth of bee colonies, although pollen quality has also proven important in this aspect (Di Pasquale et al. 2013; Pernal and Currie 2001). In light microscopic analysis, the unit used is pollen grain count, which is useful for studying plant pollination. These two units are interconvertible only if one counts the number of pollen grains in a given weight of pollen pellets of each plant species of interest. In DNA meta-barcoding analysis, the subject of measurement is totally different; the unit is the number of amplicons. This is not interconvertible with the other two units, and it is necessary to pay attention to the intrinsic bias derived from PCR.

In some cases, a vegetation survey around the sampling site will be indispensable for identifying the species in pollen pellets (as was the case with a study conducted in the Italian Alps; Galimberti et al. 2014) because there is insufficient DNA sequencing data in the database for plants in areas where flora and vegetation have not been fully studied. In contrast, in the present study, identification by DNA barcoding proved to be satisfactory only with the available floral and phenological information; therefore, vegetation surveys are nonessential, at least in Northern Hemisphere areas with similar vegetation, such as Europe and North America. Nevertheless, vegetation surveys confirm the identification reached through DNA barcoding and provide a list of possible species that were unidentifiable in the molecular biological investigation. For example, with *Plantago* sp. (no. 21 in Table 1), because discrimination was not achieved at the species level by reconstructing the phylogenetic tree, identification was terminated at the genus level. However, the vegetation survey revealed that two species of this genus, *Plantago lanceolata* L. and *Plantago asiatica* L., were frequently observed in grasslands (Table S4 in the ESM). As *P. lanceolata* (no. 20) was identified by DNA barcoding, *Plantago* sp. (no. 21) is likely to be *P. asiatica*. Another example is that of *Parasenecio* sp. (no. 19). Since *Parasenecio hastatus* (L.) H. Koyama subsp. *orientalis* was the only species observed in the vegetation survey (Table S4 in the ESM), this would be the first to be investigated in the case that further detailed identification was needed for pollen pellet no. 19.

The flowering season of each species examined should be taken into consideration. The identification of *A. polygama* (no. 1 in Table 1) seems reasonable, even though it did not appear in the vegetation survey as frequently as other species in this genus: *Actinidia arguta* (Siebold et Zucc.) Planch. ex Miq. and *Actinidia kolomikta* (Maxim. et Rupr.) Maxim. (Table S4 in the ESM). In Bibai, approximately 130 km south of Shibetsu City, *A. arguta* blooms from mid- to late June, *A. kolomikta* blooms from early to mid-July, and *A. polygama* blooms from mid- to late July (Yamaguchi and Kikuzawa 1993). We started sampling in late July, which is why *A. polygama* was the only species from the genus *Actinidia* detected in our study.

The appropriate number of pollen pellets in a sample depends on the information that researchers aim to acquire. We decided to use 48 pollen pellets per sample for technical reasons. Because we sampled the same number of pellets from each hive on each date regardless of the total number of pellets collected, the sampling ratio varied from 1.1 to 27.6% by weight of pellets (Table S1 of the ESM). This sampling method could be acceptable when attempting to elucidate the proportional composition of the main species used by honeybees. However, further investigation should be carried out to identify the minimum number of pellets needed for an accurate analysis of species composition, as is discussed in a report using pollen grains (Lau et al. 2017).

## Conclusion

To elucidate appropriate landscape management around apiaries, including the planting of additional flower resources, it is essential to be able to recognize the composition of species that honeybees use as the seasons change. The method for analyzing pollen pellets developed in the present study is the method best suited to this task. Light microscopic analysis is a lower-cost alternative method for achieving this goal, but it requires more expertise and its power to discriminate to species level is generally weak. Multiplexed next-generation sequencing would undoubtedly be the best solution for detecting as many of the species present in a large mixture of pollen pellets as possible, as also reported by other researchers.

## Electronic supplementary material

Below is the link to the electronic supplementary material.

Supplementary material 1 (PDF 58 kb)

Supplementary material 2 (PDF 66 kb)

Supplementary material 3 (PDF 65 kb)

Supplementary material 4 (PDF 71 kb)

Supplementary material 5 (PDF 214 kb)

Supplementary material 6 (PDF 221 kb)

Supplementary material 7 (PDF 125 kb)

Supplementary material 8 (PDF 124 kb)
