# Supplementary material for: A DNA barcoding method for identifying and quantifying the composition of pollen species collected by European honeybees, Apis mellifera (Hymenoptera: Apidae)
Source: Appl Entomol Zool. 2018 May 16;53(3):353–61. doi: 10.1007/s13355-018-0565-9 (PMC6060998; doi:10.1007/s13355-018-0565-9)
Supplement: Supplementary file 1 — Supplementary material 1 (PDF 58 kb) [file 13355_2018_565_MOESM1_ESM.pdf]

Table S1 Weights of pollen pellets trapped at Site 5 in Shibetsu City, Hokkaido

	Date	Sampling time	Trapped pollen pellets (g fresh weight/h)
1	21 July 2016	10:18–13:18	2.94
2	26 July 2016	10:20–13:20	2.41
3	3 August 2016	09:35–12:00	4.26
4	10 August 2016	08:56–11:56	1.56
5	18 August 2016	09:45–12:39	6.44
6	25 August 2016	09:05–12:05	10.04
7	2 September 2016	09:05–12:05	8.01
8	8 September 2016	08:35–11:35	2.99
9	14 September 2016	09:11–12:11	2.20
