# Supplementary material for: A DNA barcoding method for identifying and quantifying the composition of pollen species collected by European honeybees, Apis mellifera (Hymenoptera: Apidae)
Source: Appl Entomol Zool. 2018 May 16;53(3):353–61. doi: 10.1007/s13355-018-0565-9 (PMC6060998; doi:10.1007/s13355-018-0565-9)
Supplement: Supplementary file 2 — Supplementary material 2 (PDF 66 kb) [file 13355_2018_565_MOESM2_ESM.pdf]

Table S2 *trnL-trnF* molecular identification of honeybee pollen pellets collected in Hokkaido, northern Japan

No.[a]	Identified plant	Species in a monophyletic group with a pollen pellet in the phylogenetic tree [b]	
		Distributed in study area	Not reported in study area
1	*Actinidia polygama*	*Actinidia polygama*	*Actinidia valvata* var. *valvata*
2	*Angelica* sp.	—	*Angelica nitida*
3	*Artemisia* sp.	*Artemisia indica* var. *maximowiczii*, *Artemisia montana*	*Artemisia argyi*
4	*Asparagus officinalis*	*Asparagus officinalis*	—
5	*Chelidonium majus*	*Chelidonium majus*	—
6	*Chenopodium album*	*Chenopodium album*	*Chenopodium gigantospermum* var. *standleyanum*
7	*Cirsium vulgare*	*Cirsium vulgare*	*Cirsium carniolicum* subsp. *rufescens*, *Cirsium echinus*, *Cirsium lidjiangense*
10	*Fagopyrum esculentum*	*Fagopyrum esculentum*	—
11	*Fallopia sachalinensis*	*Fallopia sachalinensis*	*Fallopia japonica*
12	*Filipendula* sp.	—	*Filipendula ulmaria*
13	*Hydrangea paniculata*	*Hydrangea paniculata*	*Hydrangea heteromalla*
14	*Hydrangea petiolaris*	*Hydrangea petiolaris*	—
15	*Hypochaeris radicata*	*Hypochaeris radicata*	*Hypochaeris glabra*
16	*Kalopanax septemlobus*	*Kalopanax septemlobus*	—
18	*Oryza sativa*	*Oryza sativa*	*Oryza barthii*, *Oryza glaberrima*, *Oryza glumipatula*, *Oryza meridionalis*, *Oryza nivara*, *Oryza rufipogon*
19	*Parasenecio* sp.	—	*Parasenecio deltophyllus*, *Parasenecio maowenensis*
20	*Plantago* sp.	—	*Plantago argentea*, *Plantago leiopetala*
22	*Rudbeckia hirta* [c]	*Rudbeckia hirta*	*Ratibida columnaris*
25	*Solanum nigrum*	*Solanum nigrum*	*Solanum opacum*, *Solanum retroflexum*, *Solanum scabrum*, *Solanum tarderemotum*, *Solanum umalilaense*, *Solanum villosum*
26	*Solidago gigantea*	*Solidago gigantea*	*Solidago elongata*
27	*Solidago* sp.	—	*Solidago decurrens*, *Solidago litoralis*
28	*Tilia* sp.	*Tilia japonica*, *Tilia miqueliana*	*Tilia amurensis*, *Tilia mandshurica*, *Tilia megaphylla*, *Tilia oliveri*, *Tilia paucicostata*, *Tilia rufa*, *Tilia taquetii*
29	*Trifolium* sp.	*Trifolium pratense*, *Trifolium repens*	*Trifolium alpestre*, *Trifolium caudatum*, *Trifolium heldreichianum*, *Trifolium pannonicum*, *Trifolium velebiticum*
30	*Trifolium repens*	*Trifolium repens*	—
31	*Zea mays*	*Zea mays*	*Zea diploperennis*, *Zea luxurians*, *Zea nicaraguensis*, *Zea perennis*

[a] Pollen pellets No. 8, 9, 17, 21, 23, and 24 showed no amplification in PCR.
[b] Subclade(s) within a monophyletic group were excluded. Floral and phenological information was confirmed by reference to the literature (Ohashi et al. 2015, 2016a, 2016b, 2017a, 2017b; Shimizu 2003).
[c] Together with identification using ITS2, No. 22 was identified as *Rudbeckia laciniata* (see Table 1)
