# Supplementary material for: A DNA barcoding method for identifying and quantifying the composition of pollen species collected by European honeybees, Apis mellifera (Hymenoptera: Apidae)
Source: Appl Entomol Zool. 2018 May 16;53(3):353–61. doi: 10.1007/s13355-018-0565-9 (PMC6060998; doi:10.1007/s13355-018-0565-9)
Supplement: Supplementary file 3 — Supplementary material 3 (PDF 65 kb) [file 13355_2018_565_MOESM3_ESM.pdf]

Table S3 ITS2 molecular identification of honeybee pollen pellets collected in Hokkaido, northern Japan

No.[a]	Identified plant	Species in a monophyletic group with a pollen pellet in the phylogenetic tree [b]	
		Distributed in study area	Not reported in study area
1	*Actinidia polygama*	*Actinidia polygama*	—
2	*Angelica* sp.	—	*Angelica dahurica* var. *formosana*
3	*Artemisia montana*	*Artemisia montana*	*Artemisia stelleriana*
4	*Asparagus officinalis*	*Asparagus officinalis*	—
5	*Chelidonium majus*	*Chelidonium majus*	—
6	*Chenopodium* sp.	*Chenopodium album*, *Chenopodium murale*	*Chenopodium acuminatum* subsp. *virgatum*, *Chenopodium berlandieri*, *Chenopodium giganteum*, *Chenopodium gigantospermum* var. *standleyanum*, *Chenopodium opulifolium*, *Chenopodium novopokrovskyanum*
7	*Cirsium vulgare*	*Cirsium vulgare*	*Cirsium muticum*
9	*Cucurbita maxima*	*Cucurbita maxima*	—
12	*Filipendula camtschatica*	*Filipendula camtschatica*	—
13	*Hydrangea paniculata*	*Hydrangea paniculata*	—
15	*Hypochaeris radicata*	*Hypochaeris radicata*	*Agoseris elata*, *Taraxacum alpinum*
16	*Kalopanax septemlobus*	*Kalopanax septemlobus*	—
19	Senecioneae		*Cremanthodium humile*, *Parasenecio adenostyloides*, *Parasenecio firmus*, *Parasenecio subglaber*, *Syneilesis palmata*
20	*Plantago lanceolata*	*Plantago lanceolata*	*Plantago lagopus*, *Plantago leiopetala*
21	*Plantago* sp.	*Plantago asiatica*, *Plantago major*	*Plantago cornuti*, *Plantago erosa*, *Plantago hostifolia*, *Plantago uniglumis*
22	*Rudbeckia laciniata*	*Rudbeckia laciniata*	—
23	*Sagittaria trifolia*	*Sagittaria trifolia*	—
24	*Sinapis alba*	*Sinapis alba*	—
25	*Solanum nigrum*	*Solanum nigrum*	*Solanum americanum*, *Solanum clavatum*, *Solanum physalifolium*
26	*Solidago gigantea*	*Solidago gigantea*	—
27	*Solidago virgaurea*	*Solidago virgaurea*	—
28	*Tilia* sp.	*Tilia japonica*, *Tilia maximowicziana*, *Tilia miqueliana*	*Tilia koreana*, *Tilia oliveri*, *Tilia tuan*
29	*Trifolium pratense*	*Trifolium pratense*	*Trifolium caudatum*, *Trifolium cherleri*
30	*Trifolium repens*	*Trifolium repens*	—

[a] Pollen pellets No. 8, 10, 11, 14, 17, 18, and 31 showed no amplification in PCR.
[b] Subclade(s) within a monophyletic group were excluded. Floral and phenological information was confirmed by reference to the literature (Ohashi et al. 2015, 2016a, 2016b, 2017a, 2017b; Shimizu 2003).
