# Supplementary material for: A DNA barcoding method for identifying and quantifying the composition of pollen species collected by European honeybees, Apis mellifera (Hymenoptera: Apidae)
Source: Appl Entomol Zool. 2018 May 16;53(3):353–61. doi: 10.1007/s13355-018-0565-9 (PMC6060998; doi:10.1007/s13355-018-0565-9)
Supplement: Supplementary file 4 — Supplementary material 4 (PDF 71 kb) [file 13355_2018_565_MOESM4_ESM.pdf]

Table S4 Plant species that were observed in a vegetation survey and that belong to the same genera as were identified in the pollen pellets

No. of pollen pellet[a]	Plant species observed in vegetation survey	Appearance (%)[b]	
		Forest	Grassland
1	*Actinidia arguta*	51.1	0.7
	Actinidia kolomikta	26.7	0.0
	Actinidia polygama	6.7	0.7
2	*Angelica anomala*	2.2	0.0
	Angelica ursina	22.2	2.8
3	*Artemisia montana*	28.9	21.3
	Artemisia indica var. *maximowiczii*	0.0	10.6
5	*Chelidonium majus* var. *asiaticum*	4.4	0.7
6	*Chenopodium album*	2.2	1.4
7	*Cirsium kamtschaticum*	17.8	0.7
	Cirsium vulgare	0.0	1.4
	Cirsium sp.	6.7	1.4
8	*Commelina communis*	0.0	5.0
10	*Fagopyrum esculentum*	0.0	0.7
11	*Fallopia convolvulus*	0.0	0.7
	Fallopia sachalinensis	24.4	11.3
12	*Filipendula camtschatica*	11.1	2.8
13, 14	*Hydrangea paniculata*	22.2	0.0
	Hydrangea petiolaris	60.0	0.0
	Hydrangea serrata var. *yesoensis*	0.0	1.4
15	*Hypochaeris radicata*	0.0	14.9
16	*Kalopanax septemlobus*	71.1	0.0
19	*Parasenecio hastatus* subsp. *orientalis*	20.0	1.4
20, 21	*Plantago asiatica*	13.3	21.3
	Plantago lanceolata	0.0	21.3
22	*Rudbeckia laciniata*	15.6	11.3
26, 27	*Solidago gigantea* subsp. *serotina*	4.4	8.5
	Solidago virgaurea subsp. *asiatica*	24.4	1.4
28	*Tilia japonica*	80.0	0.7
	Tilia maximowicziana	40.0	0.0
29, 30	*Trifolium pratense*	0.0	22.7
	Trifolium repens	0.0	41.1

[a] Numbering of pollen pellets is identical to that used in Tables 1, S2, and S3. Because farmland was not included in the vegetation survey, vegetables (No. 4 and 9), a field crop (No. 31), companion weeds in paddy fields (No. 17 and 23), and a plant used as green manure (No. 24) were not observed.

[b] The vegetation survey was conducted in forest (*n*=45) and grassland (*n*=141)

To validate the identification of species shown in Table 1, a vegetation survey was conducted from 15 to 22 August 2016 (Table S4). We recorded all species and their cover values (%) in 1 m × 1 m quadrats: 141 quadrats were set up on typical herbaceous vegetation in the study area, and 45 quadrats were set up to survey typical forest vegetation by the methods of Braun-Blanquet. The vegetation survey recorded 391 plant species. The species corresponding to those found during the identification of pollen pellets, together with their closely related species, are listed here. Twenty of the species detected during the pollen pellet identification were also recorded in the vegetation survey. For the four taxa identified to the genus level (*Angelica* sp., *Parasenecio* sp., *Plantago* sp., and *Tilia* sp.), the species shown in Table S4 represent the estimated species for each taxon. Although *Asparagus officinalis*, *Cucurbita maxima*, *Oryza sativa*, *Sinapis alba*, and *Zea mays* were not recorded due to the exclusion of farmland from the vegetation survey, these crops, vegetables, and green manures were widely cultivated in the surveyed area. Similarly, *Monochoria korsakowii* and *Sagittaria trifolia*, which are companion weeds in paddy fields, were not recorded in the vegetation survey. The sole exception was *Solanum nigrum*, which was detected during pollen pellet identification but, for no plausible reason, was not recorded in the vegetation survey.
