# Supplementary material for: A DNA barcoding method for identifying and quantifying the composition of pollen species collected by European honeybees, Apis mellifera (Hymenoptera: Apidae)
Source: Appl Entomol Zool. 2018 May 16;53(3):353–61. doi: 10.1007/s13355-018-0565-9 (PMC6060998; doi:10.1007/s13355-018-0565-9)
Supplement: Supplementary file 5 — Supplementary material 5 (PDF 214 kb) [file 13355_2018_565_MOESM5_ESM.pdf]

Fig. S1 Molecular phylogenetic trees of honeybee pollen pellets by maximum likelihood method in *trnL-trnF*

The evolutionary history was inferred by using the maximum likelihood method based on the Tamura–Nei model. The tree with the highest log likelihood is shown. The percentage of trees in which the associated taxa clustered together is shown next to the branches. Initial tree(s) for the heuristic search were obtained automatically by applying the Neighbor-Join and BioNJ algorithms to a matrix of pairwise distances estimated using the maximum composite likelihood approach, and then selecting the topology with superior log likelihood value. Each tree is drawn to scale, with branch lengths indicating the number of substitutions per site. The analysis involved 10–21 nucleotide sequences. All positions containing gaps and missing data were eliminated. Evolutionary analyses were conducted in MEGA7. The numbering of honeybee pollen pellets is as follows: (a) No. 1; (b) No. 2; (c) No. 3; (d) No. 4; (e) No. 5; (f) No. 6; (g) No. 7; (h) No. 10; (i) No. 11; (j) No. 12; (k) No. 13; (l) No. 14; (m) No. 15; (n) No. 16; (o) No. 18; (p) No. 19; (q) No. 20; (r) No. 22; (s) No. 25; (t) No. 26; (u) No. 27; (v) No. 28; (w) No. 29; (x) No. 30; (y) No. 31.

(a)

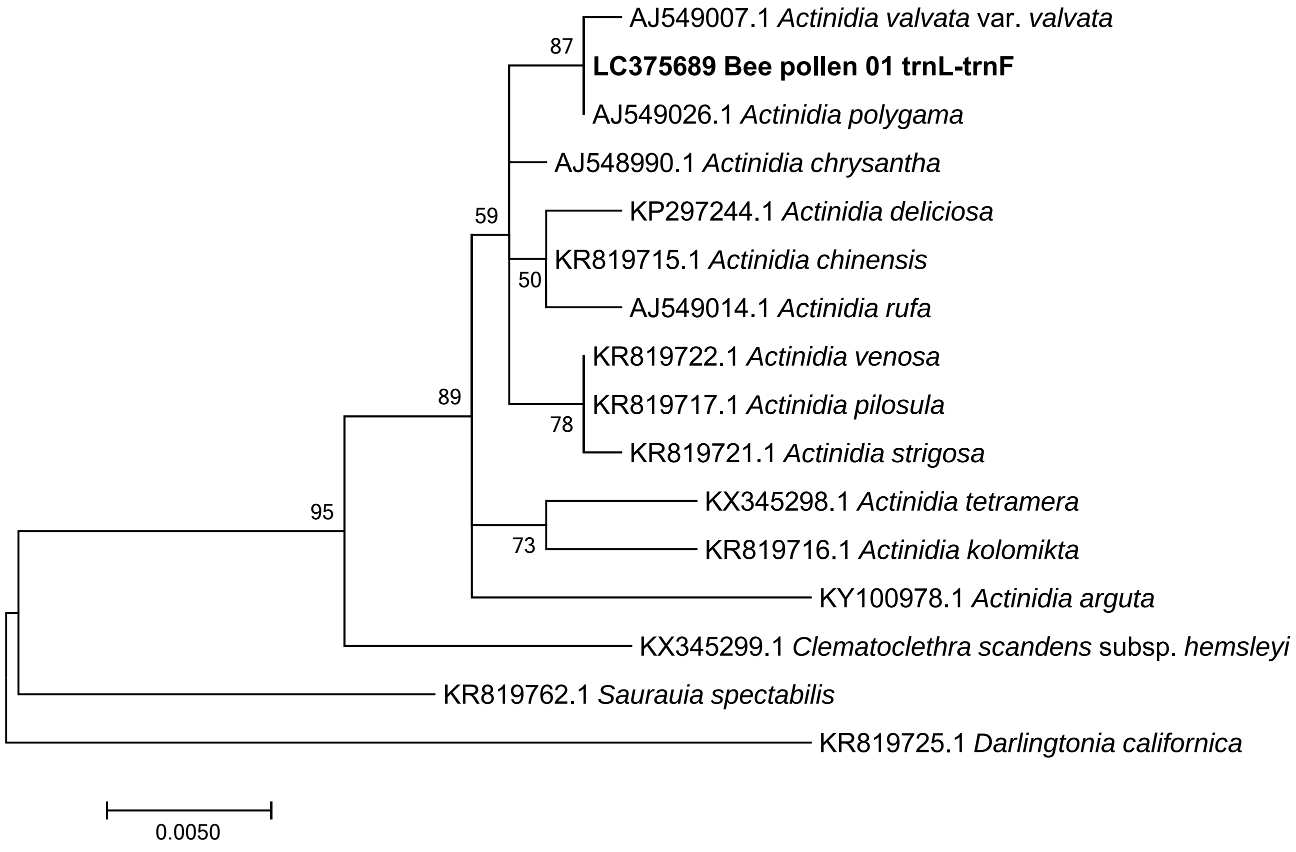
AJ549007.1 Actinidia valvata var. valvata
87
LC375689 Bee pollen 01 trnL-trnF
AJ549026.1 Actinidia polygama
AJ548990.1 Actinidia chrysantha
59
KP297244.1 Actinidia deliciosa
KR819715.1 Actinidia chinensis
50
AJ549014.1 Actinidia rufa
KR819722.1 Actinidia venosa
89
KR819717.1 Actinidia pilosula
78
KR819721.1 Actinidia strigosa
95
KX345298.1 Actinidia tetramera
73
KR819716.1 Actinidia kolomikta
KY100978.1 Actinidia arguta
KX345299.1 Clematoclethra scandens subsp. hemsleyi
KR819762.1 Saurauia spectabilis
KR819725.1 Darlingtonia californica
0.0050

(b)

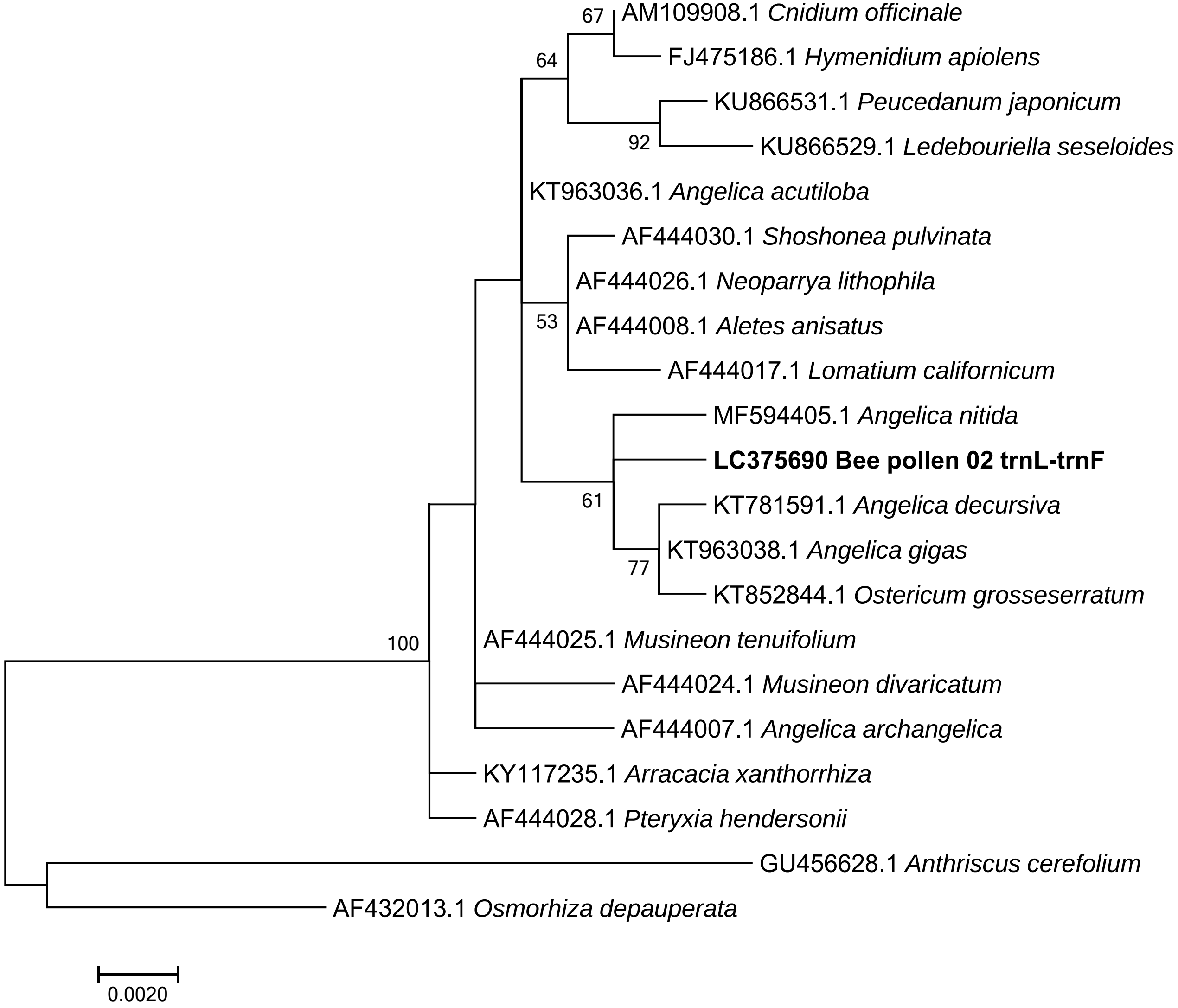
67
AM109908.1 Cnidium officinale
64
FJ475186.1 Hymenidium apiolens
KU866531.1 Peucedanum japonicum
92
KU866529.1 Ledebouriella seseloides
KT963036.1 Angelica acutiloba
AF444030.1 Shoshonea pulvinata
AF444026.1 Neoparrya lithophila
53
AF444008.1 Aletes anisatus
AF444017.1 Lomatium californicum
MF594405.1 Angelica nitida
LC375690 Bee pollen 02 trnL-trnF
61
KT781591.1 Angelica decursiva
KT963038.1 Angelica gigas
77
KT852844.1 Ostericum grosseserratum
100
AF444025.1 Musineon tenuifolium
AF444024.1 Musineon divaricatum
AF444007.1 Angelica archangelica
KY117235.1 Arracacia xanthorrhiza
AF444028.1 Pteryxia hendersonii
GU456628.1 Anthriscus cerefolium
AF432013.1 Osmorhiza depauperata
0.0020

(c)

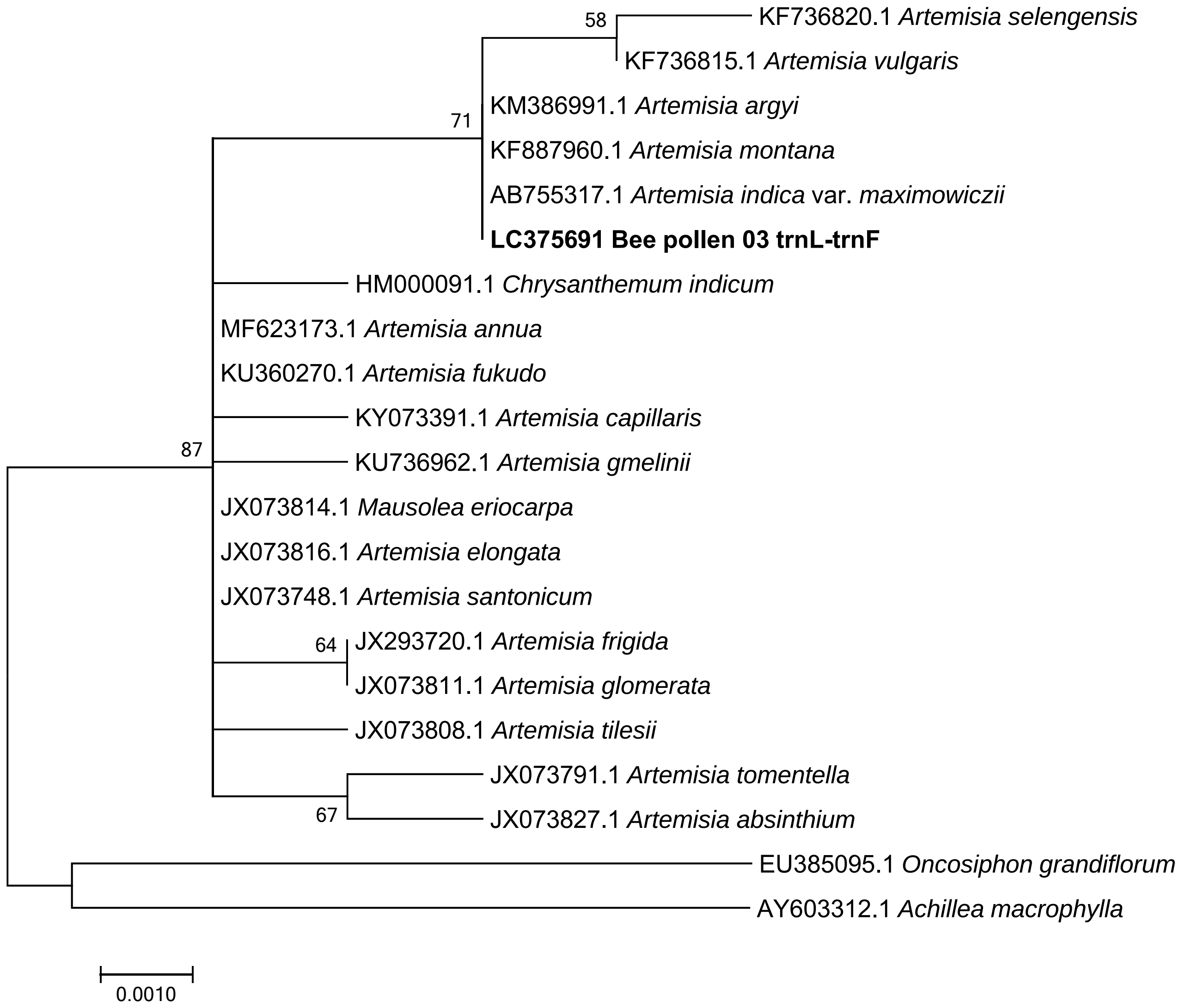
58
KF736820.1 Artemisia selengensis
KF736815.1 Artemisia vulgaris
71
KM386991.1 Artemisia argyi
KF887960.1 Artemisia montana
AB755317.1 Artemisia indica var. maximowiczii
LC375691 Bee pollen 03 trnL-trnF
HM000091.1 Chrysanthemum indicum
MF623173.1 Artemisia annua
KU360270.1 Artemisia fukudo
KY073391.1 Artemisia capillaris
87
KU736962.1 Artemisia gmelinii
JX073814.1 Mausolea eriocarpa
JX073816.1 Artemisia elongata
JX073748.1 Artemisia santonicum
64
JX293720.1 Artemisia frigida
JX073811.1 Artemisia glomerata
JX073808.1 Artemisia tilesii
JX073791.1 Artemisia tomentella
67
JX073827.1 Artemisia absinthium
EU385095.1 Oncosiphon grandiflorum
AY603312.1 Achillea macrophylla
0.0010

(d)

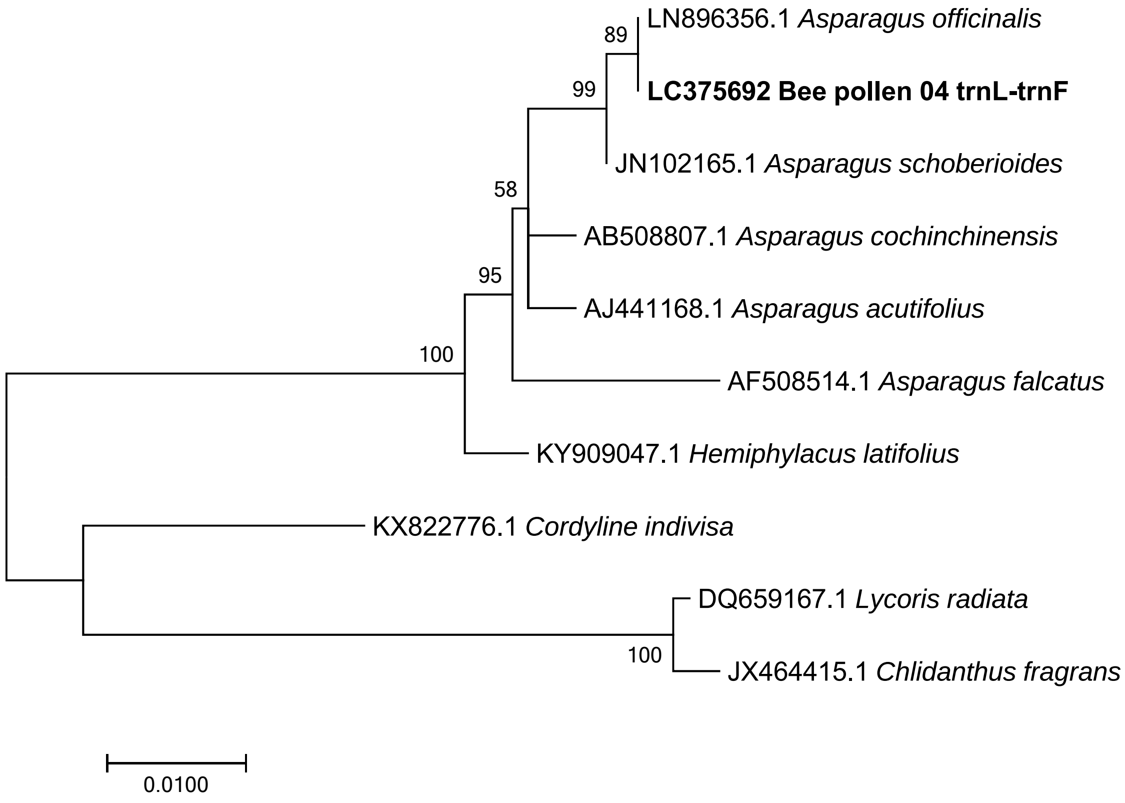

LN896356.1 *Asparagus officinalis*
89
LC375692 Bee pollen 04 trnL-trnF
99
JN102165.1 *Asparagus schoberioides*
58
AB508807.1 *Asparagus cochinchinensis*
95
AJ441168.1 *Asparagus acutifolius*
100
AF508514.1 *Asparagus falcatus*
KY909047.1 *Hemiphylacus latifolius*
KX822776.1 *Cordyline indivisa*
DQ659167.1 *Lycoris radiata*
100
JX464415.1 *Chlidanthus fragrans*
0.0100

(e)

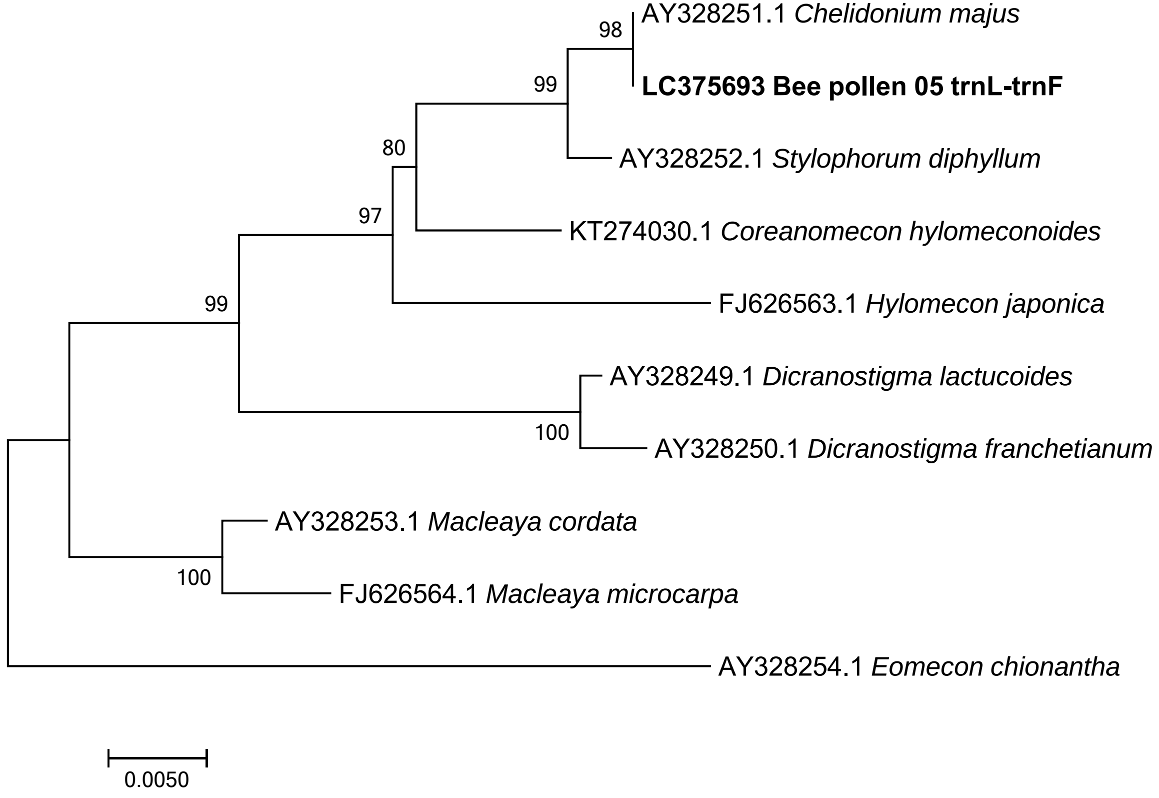

98
AY328251.1 *Chelidonium majus*
99
LC375693 Bee pollen 05 trnL-trnF
80
AY328252.1 *Stylophorum diphyllum*
97
KT274030.1 *Coreanomecon hylomeconoides*
99
FJ626563.1 *Hylomecon japonica*
AY328249.1 *Dicranostigma lactucoides*
100
AY328250.1 *Dicranostigma franchetianum*
AY328253.1 *Macleaya cordata*
100
FJ626564.1 *Macleaya microcarpa*
AY328254.1 *Eomecon chionantha*
0.0050

(f)

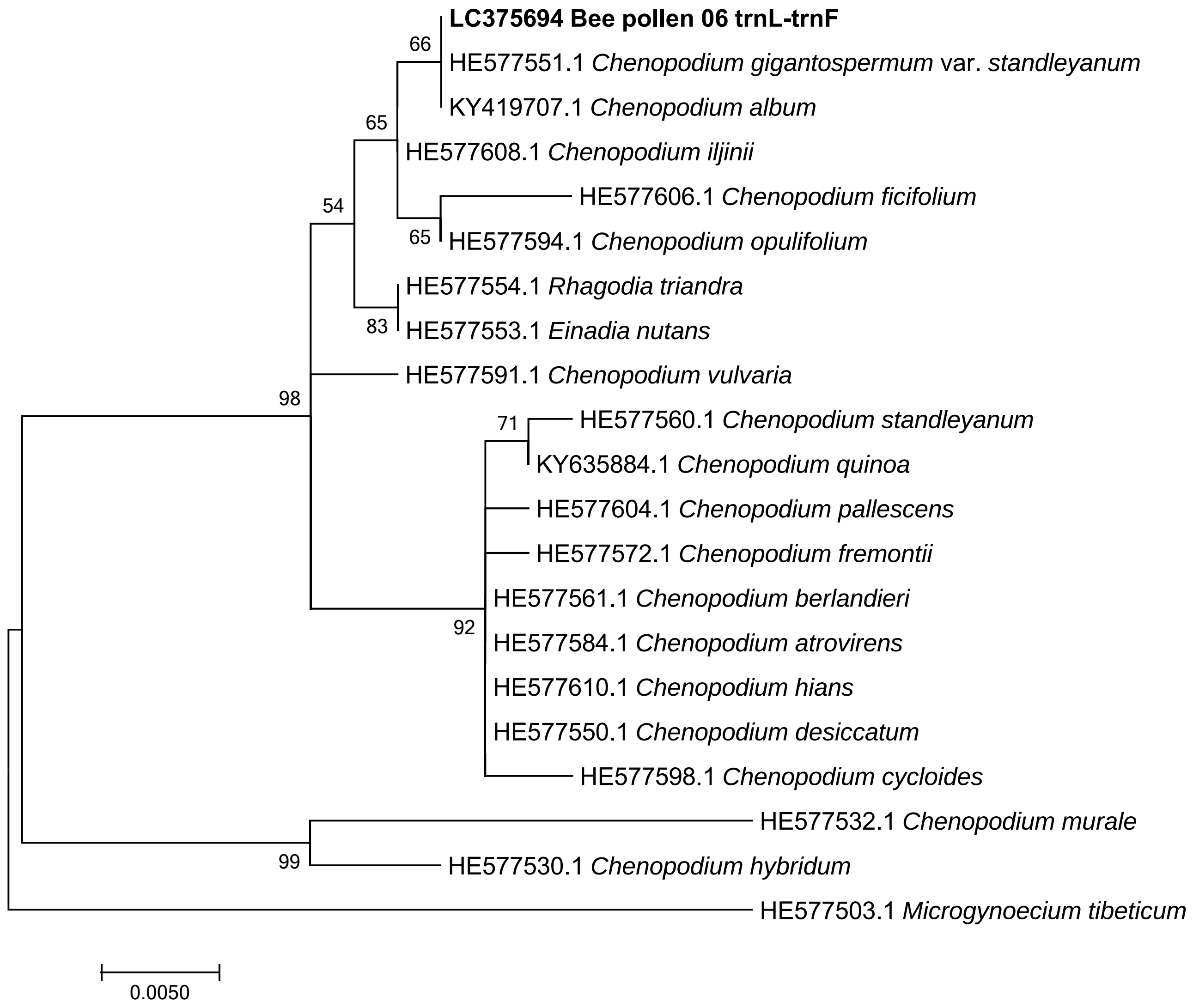
LC375694 Bee pollen 06 trnL-trnF
HE577551.1 Chenopodium gigantospermum var. standleyanum
KY419707.1 Chenopodium album
HE577608.1 Chenopodium iljinii
HE577606.1 Chenopodium ficifolium
HE577594.1 Chenopodium opulifolium
HE577554.1 Rhagodia triandra
HE577553.1 Einadia nutans
HE577591.1 Chenopodium vulvaria
HE577560.1 Chenopodium standleyanum
KY635884.1 Chenopodium quinoa
HE577604.1 Chenopodium pallescens
HE577572.1 Chenopodium fremontii
HE577561.1 Chenopodium berlandieri
HE577584.1 Chenopodium atrovirens
HE577610.1 Chenopodium hians
HE577550.1 Chenopodium desiccatum
HE577598.1 Chenopodium cycloides
HE577532.1 Chenopodium murale
HE577530.1 Chenopodium hybridum
HE577503.1 Microgynoecium tibeticum
66
65
54
65
83
98
71
92
99
0.0050

(g)

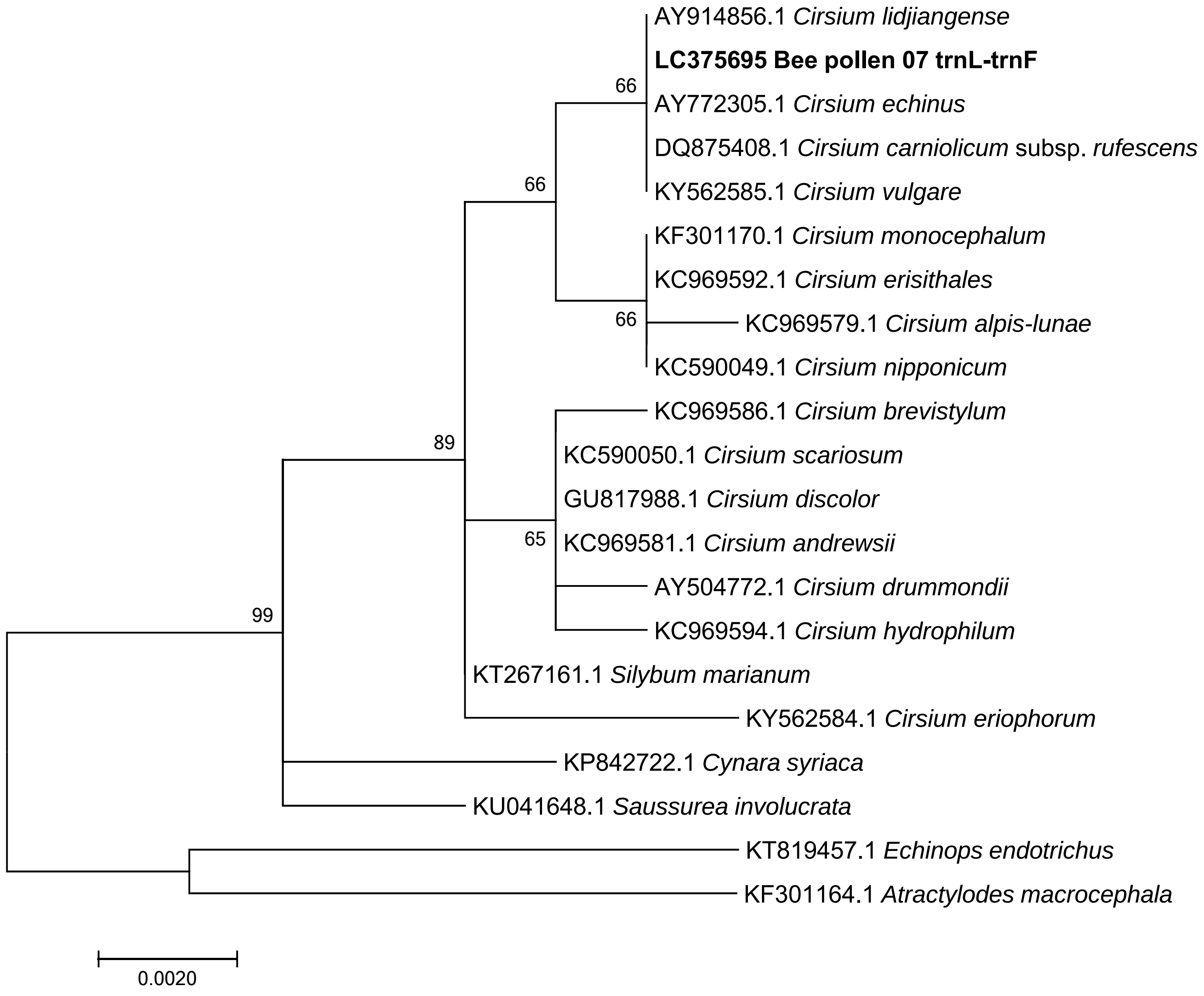

AY914856.1 *Cirsium lidjiangense*
LC375695 Bee pollen 07 trnL-trnF
AY772305.1 *Cirsium echinus*
DQ875408.1 *Cirsium carniolicum* subsp. *rufescens*
KY562585.1 *Cirsium vulgare*
KF301170.1 *Cirsium monocephalum*
KC969592.1 *Cirsium erisithales*
KC969579.1 *Cirsium alpis-lunae*
KC590049.1 *Cirsium nipponicum*
KC969586.1 *Cirsium brevistylum*
KC590050.1 *Cirsium scariosum*
GU817988.1 *Cirsium discolor*
KC969581.1 *Cirsium andrewsii*
AY504772.1 *Cirsium drummondii*
KC969594.1 *Cirsium hydrophilum*
KT267161.1 *Silybum marianum*
KY562584.1 *Cirsium eriophorum*
KP842722.1 *Cynara syriaca*
KU041648.1 *Saussurea involucrata*
KT819457.1 *Echinops endotrichus*
KF301164.1 *Atractylodes macrocephala*
66
66
66
89
65
99
0.0020

(h)

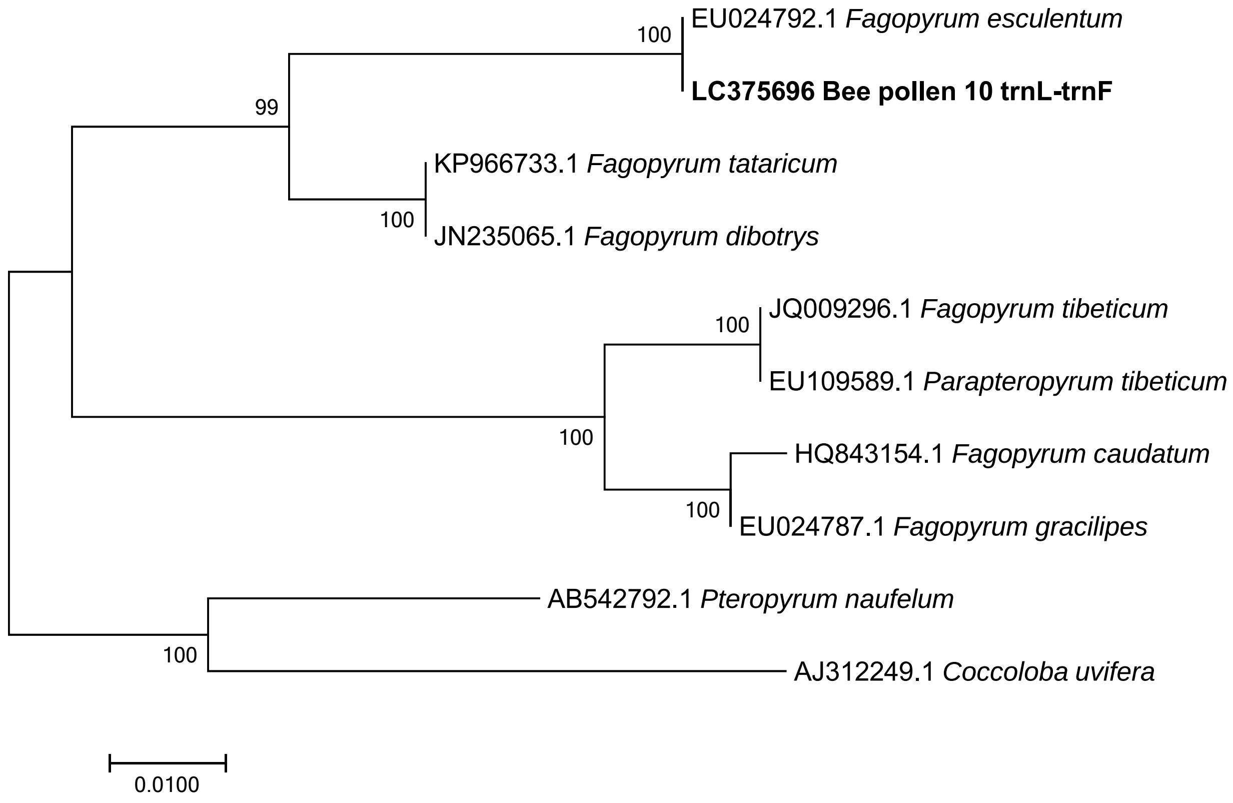

100
EU024792.1 Fagopyrum esculentum
99
LC375696 Bee pollen 10 trnL-trnF
KP966733.1 Fagopyrum tataricum
100
JN235065.1 Fagopyrum dibotrys
100
JQ009296.1 Fagopyrum tibeticum
EU109589.1 Parapteropyrum tibeticum
100
HQ843154.1 Fagopyrum caudatum
100
EU024787.1 Fagopyrum gracilipes
AB542792.1 Pteropyrum naufelum
100
AJ312249.1 Coccoloba uvifera
0.0100

(i)

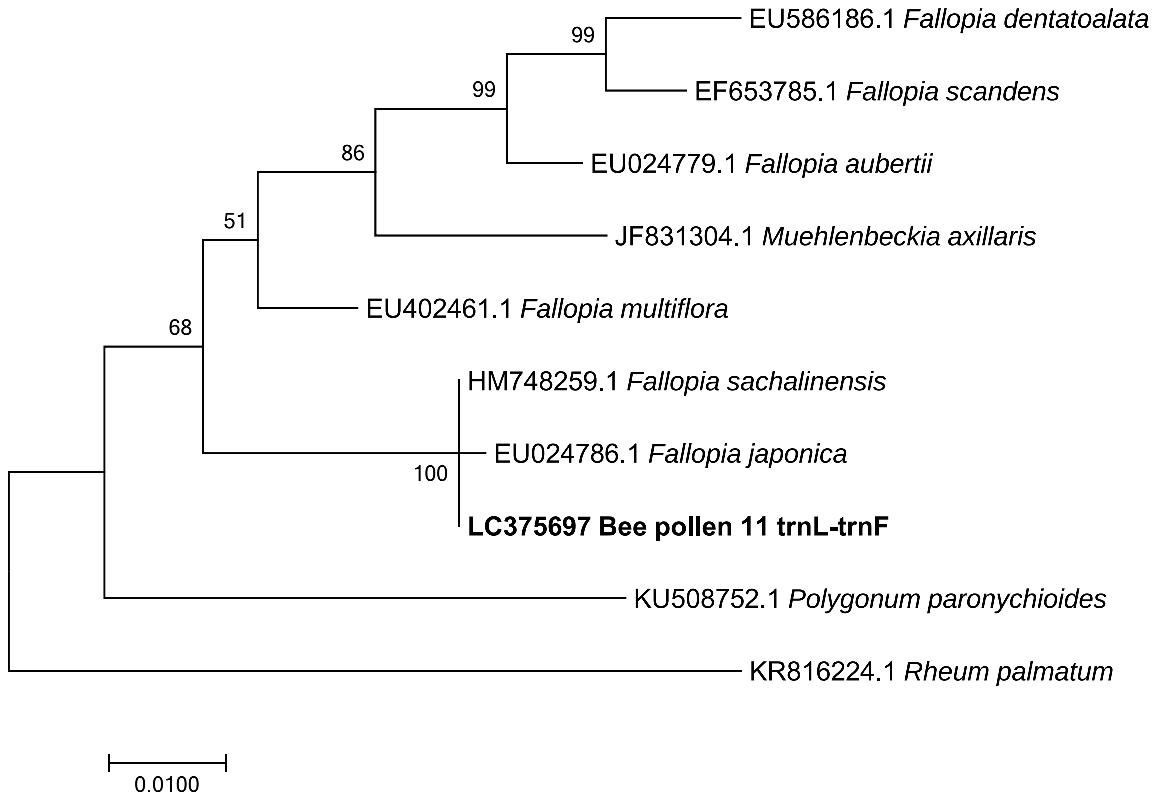
EU586186.1 Fallopia dentatoalata
EF653785.1 Fallopia scandens
EU024779.1 Fallopia aubertii
JF831304.1 Muehlenbeckia axillaris
EU402461.1 Fallopia multiflora
HM748259.1 Fallopia sachalinensis
EU024786.1 Fallopia japonica
LC375697 Bee pollen 11 trnL-trnF
KU508752.1 Polygonum paronychioides
KR816224.1 Rheum palmatum
99
99
86
51
68
100
0.0100

(j)

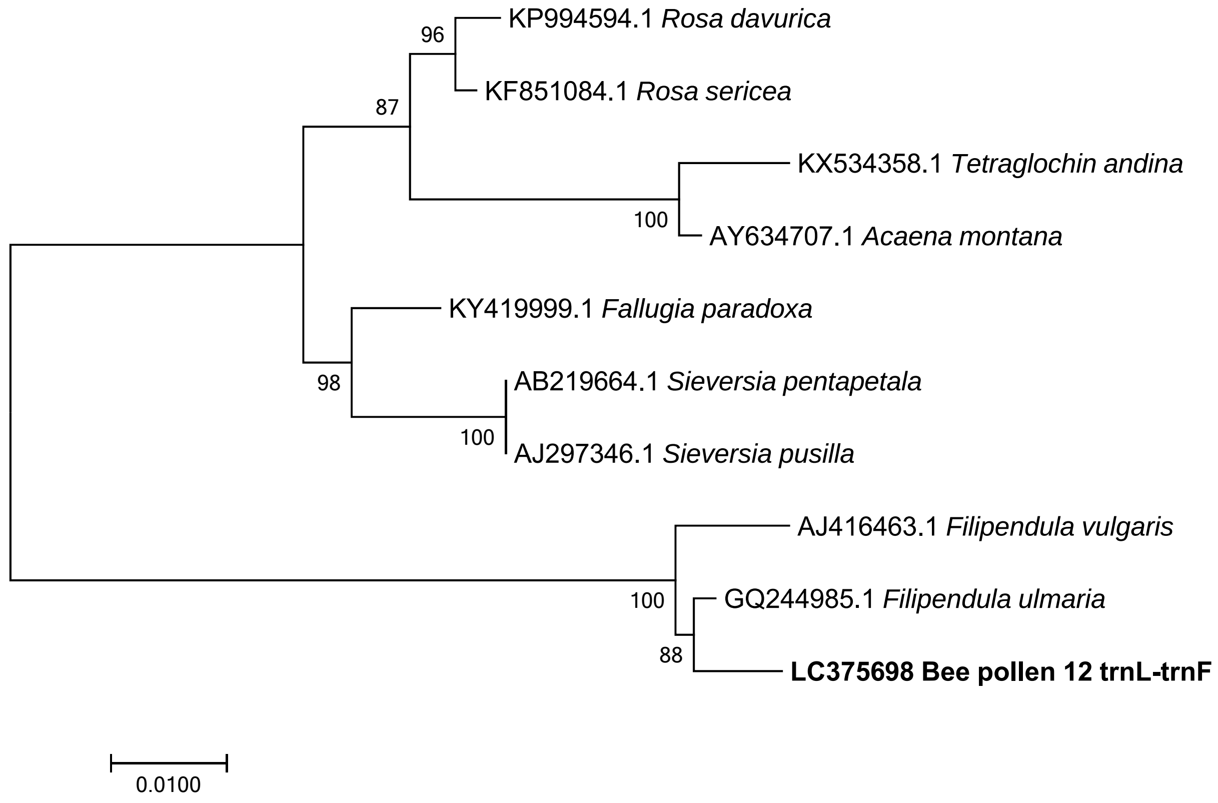
KP994594.1 *Rosa davurica*
KF851084.1 *Rosa sericea*
KX534358.1 *Tetraglochin andina*
AY634707.1 *Acaena montana*
KY419999.1 *Fallugia paradoxa*
AB219664.1 *Sieversia pentapetala*
AJ297346.1 *Sieversia pusilla*
AJ416463.1 *Filipendula vulgaris*
GQ244985.1 *Filipendula ulmaria*
LC375698 Bee pollen 12 trnL-trnF
96
87
100
98
100
100
88
0.0100

(k)

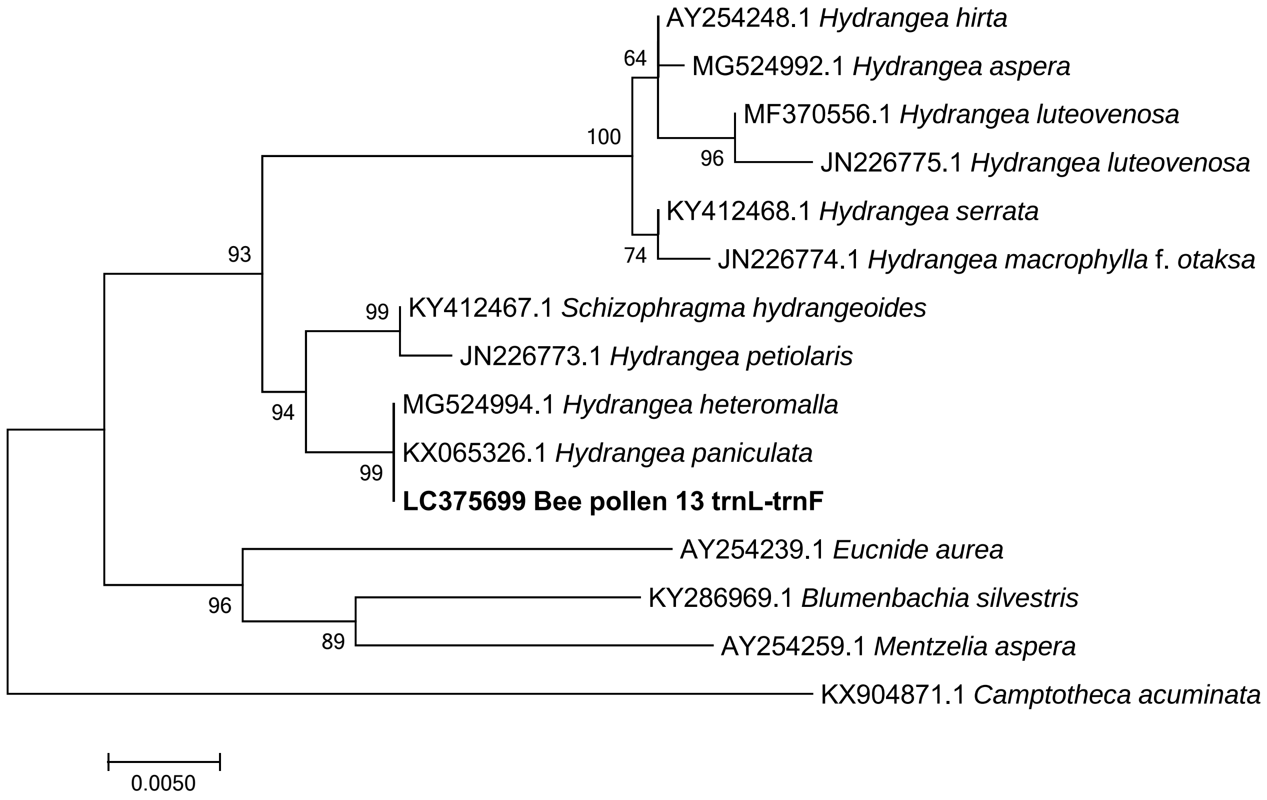
AY254248.1 Hydrangea hirta
64
MG524992.1 Hydrangea aspera
MF370556.1 Hydrangea luteovenosa
100
96
JN226775.1 Hydrangea luteovenosa
KY412468.1 Hydrangea serrata
93
74
JN226774.1 Hydrangea macrophylla f. otaksa
99
KY412467.1 Schizophragma hydrangeoides
JN226773.1 Hydrangea petiolaris
MG524994.1 Hydrangea heteromalla
94
KX065326.1 Hydrangea paniculata
99
LC375699 Bee pollen 13 trnL-trnF
AY254239.1 Eucnide aurea
KY286969.1 Blumenbachia silvestris
96
89
AY254259.1 Mentzelia aspera
KX904871.1 Camptotheca acuminata
0.0050

(I)

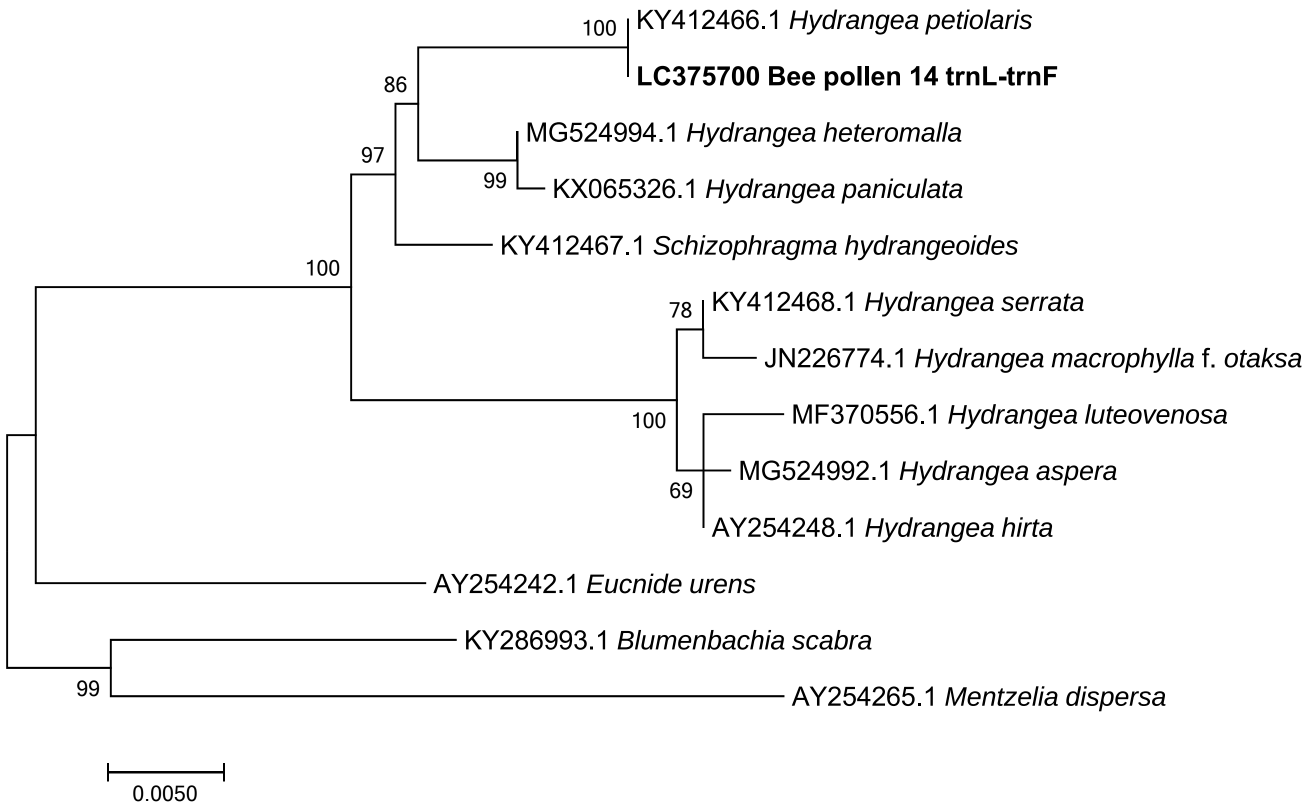
KY412466.1 Hydrangea petiolaris
LC375700 Bee pollen 14 trnL-trnF
MG524994.1 Hydrangea heteromalla
KX065326.1 Hydrangea paniculata
KY412467.1 Schizophragma hydrangeoides
KY412468.1 Hydrangea serrata
JN226774.1 Hydrangea macrophylla f. otaksa
MF370556.1 Hydrangea luteovenosa
MG524992.1 Hydrangea aspera
AY254248.1 Hydrangea hirta
AY254242.1 Eucnide urens
KY286993.1 Blumenbachia scabra
AY254265.1 Mentzelia dispersa
100
86
99
97
100
78
100
69
99
0.0050

(m)

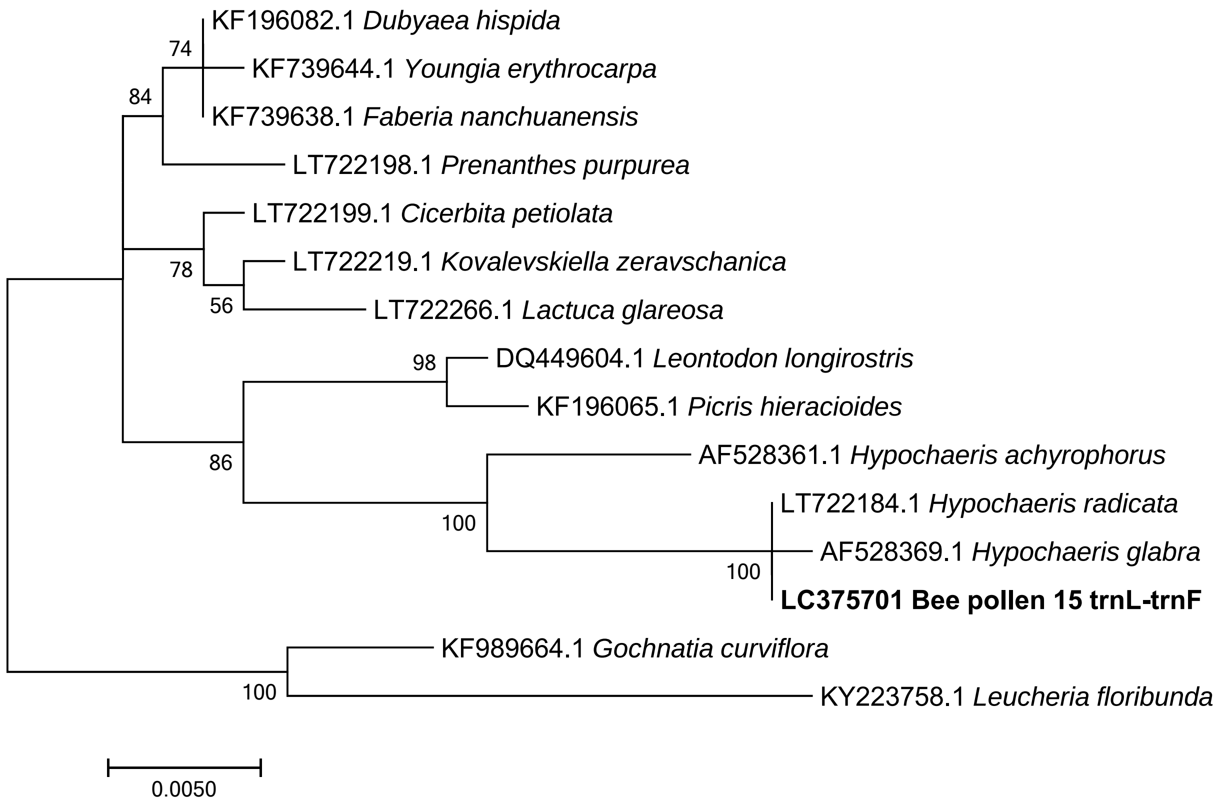
KF196082.1 *Dubyaea hispida*
74
KF739644.1 *Youngia erythrocarpa*
84
KF739638.1 *Faberia nanchuanensis*
LT722198.1 *Prenanthes purpurea*
LT722199.1 *Cicerbita petiolata*
LT722219.1 *Kovalevskiella zeravschanica*
78
56
LT722266.1 *Lactuca glareosa*
98
DQ449604.1 *Leontodon longirostris*
KF196065.1 *Picris hieracioides*
86
AF528361.1 *Hypochaeris achyrophorus*
LT722184.1 *Hypochaeris radicata*
100
AF528369.1 *Hypochaeris glabra*
100
LC375701 Bee pollen 15 trnL-trnF
KF989664.1 *Gochnatia curviflora*
100
KY223758.1 *Leucheria floribunda*
0.0050

(n)

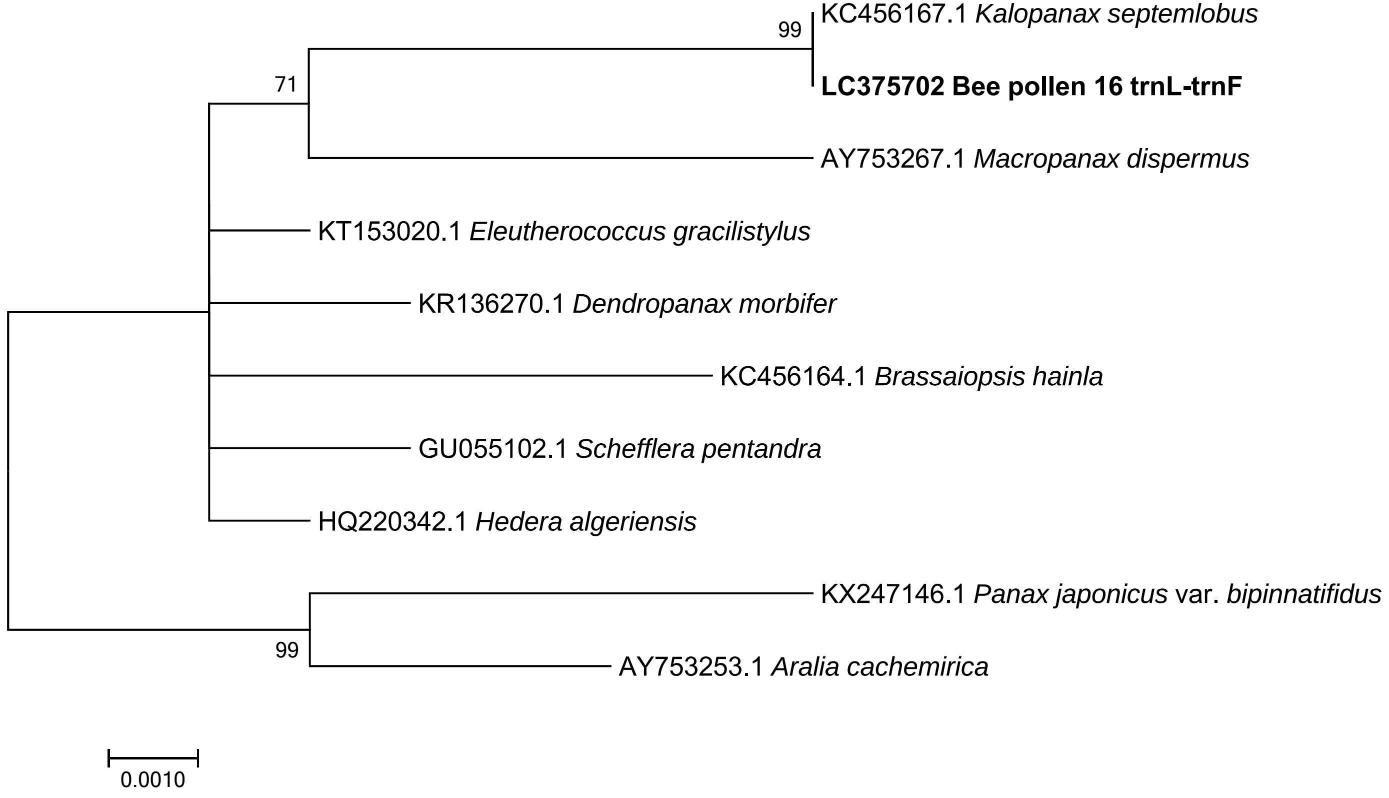
99
KC456167.1 *Kalopanax septemlobus*
71
LC375702 Bee pollen 16 trnL-trnF
AY753267.1 *Macropanax dispermus*
KT153020.1 *Eleutherococcus gracilistylus*
KR136270.1 *Dendropanax morbifer*
KC456164.1 *Brassaiopsis hainla*
GU055102.1 *Schefflera pentandra*
HQ220342.1 *Hedera algeriensis*
KX247146.1 *Panax japonicus* var. *bipinnatifidus*
99
AY753253.1 *Aralia cachemirica*
0.0010

(o)

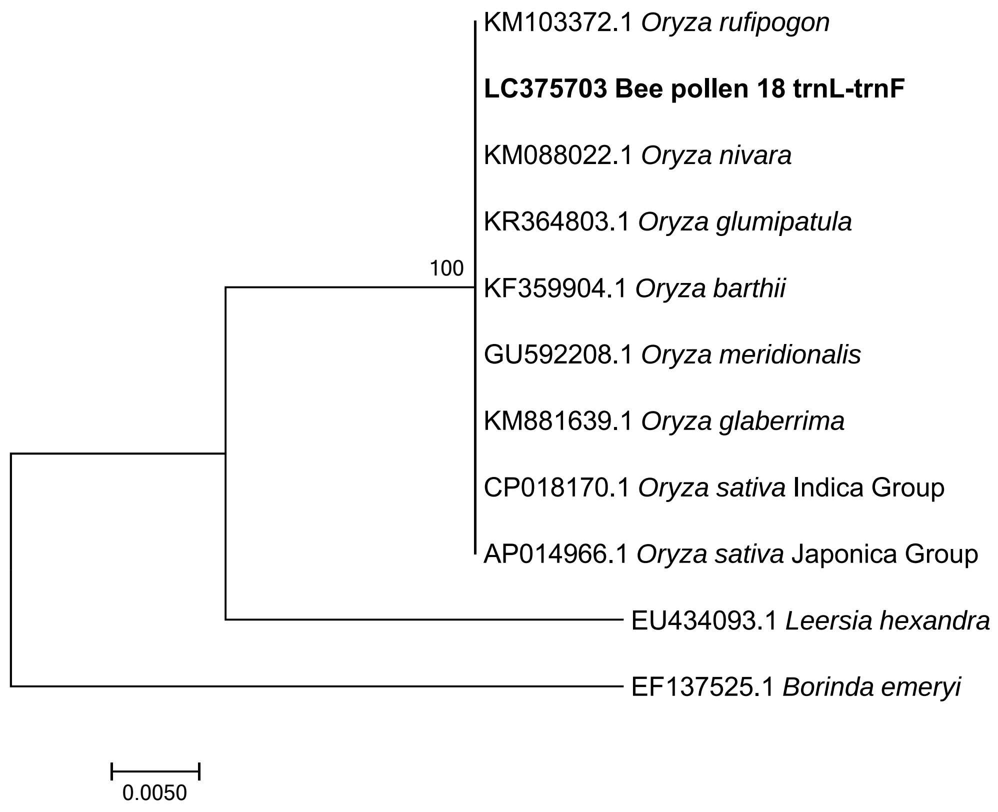
KM103372.1 Oryza rufipogon
LC375703 Bee pollen 18 trnL-trnF
KM088022.1 Oryza nivara
KR364803.1 Oryza glumipatula
100
KF359904.1 Oryza barthii
GU592208.1 Oryza meridionalis
KM881639.1 Oryza glaberrima
CP018170.1 Oryza sativa Indica Group
AP014966.1 Oryza sativa Japonica Group
EU434093.1 Leersia hexandra
EF137525.1 Borinda emeryi
0.0050

(p)

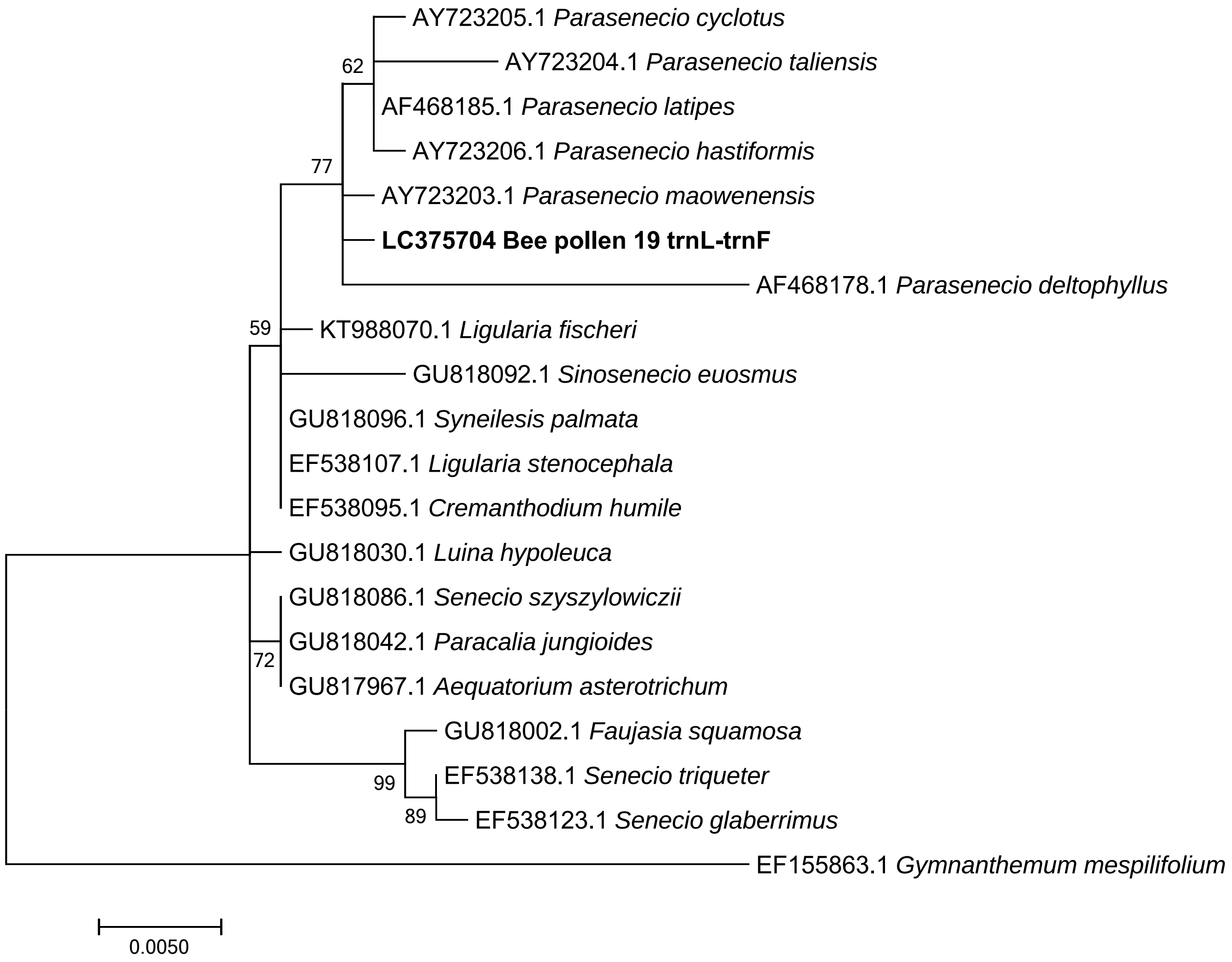
AY723205.1 Parasenecio cyclotus
AY723204.1 Parasenecio taliensis
AF468185.1 Parasenecio latipes
AY723206.1 Parasenecio hastiformis
AY723203.1 Parasenecio maowenensis
LC375704 Bee pollen 19 trnL-trnF
AF468178.1 Parasenecio deltophyllus
KT988070.1 Ligularia fischeri
GU818092.1 Sinosenecio euosmus
GU818096.1 Syneilesis palmata
EF538107.1 Ligularia stenocephala
EF538095.1 Cremanthodium humile
GU818030.1 Luina hypoleuca
GU818086.1 Senecio szyszylowiczii
GU818042.1 Paracalia jungioides
GU817967.1 Aequatorium asterotrichum
GU818002.1 Faujasia squamosa
EF538138.1 Senecio triqueter
EF538123.1 Senecio glaberrimus
EF155863.1 Gymnanthemum mespilifolium
62
77
59
72
99
89
0.0050

(q)

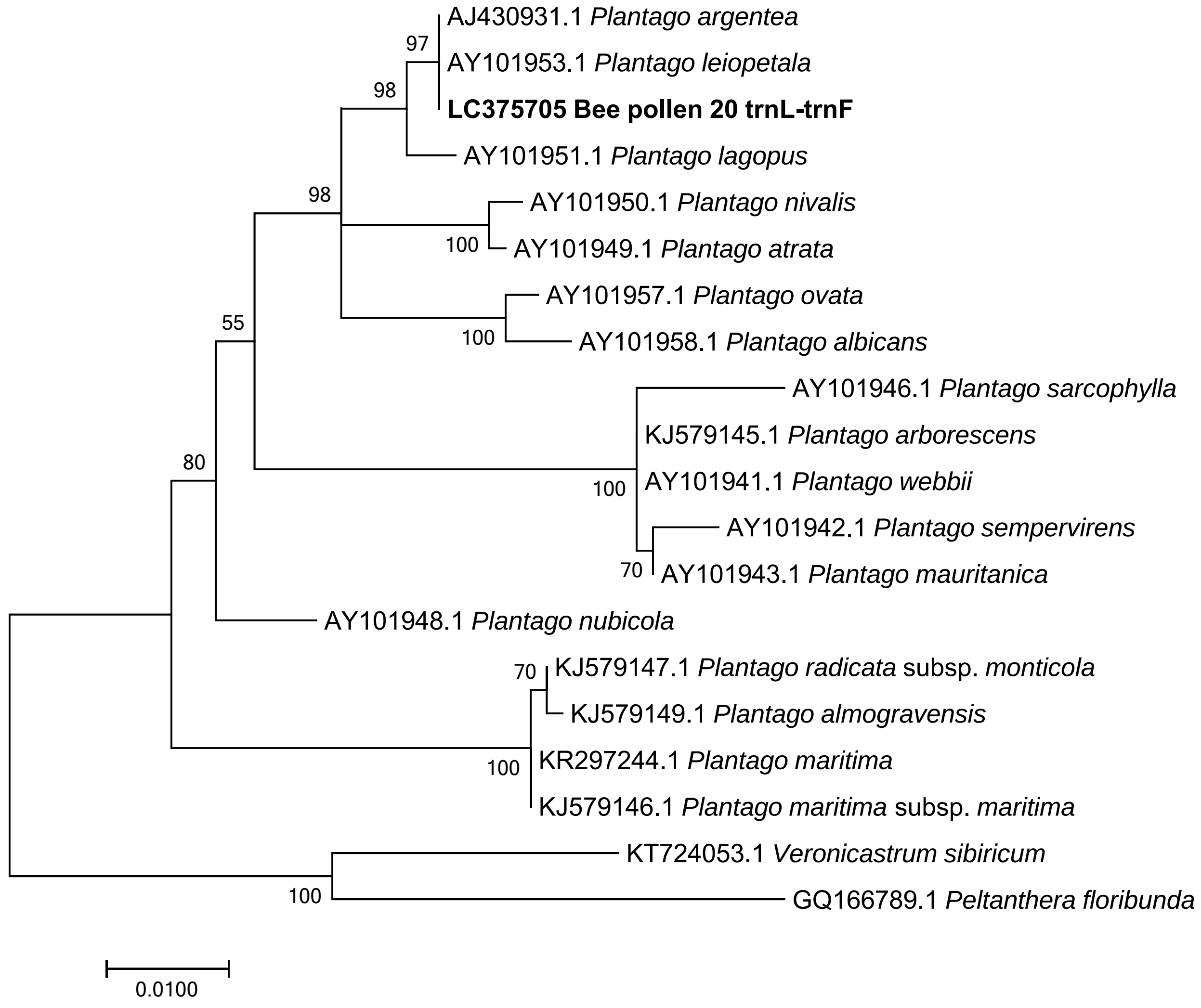
AJ430931.1 *Plantago argentea*
AY101953.1 *Plantago leiopetala*
LC375705 Bee pollen 20 trnL-trnF
AY101951.1 *Plantago lagopus*
AY101950.1 *Plantago nivalis*
AY101949.1 *Plantago atrata*
AY101957.1 *Plantago ovata*
AY101958.1 *Plantago albicans*
AY101946.1 *Plantago sarcophylla*
KJ579145.1 *Plantago arborescens*
AY101941.1 *Plantago webbii*
AY101942.1 *Plantago sempervirens*
AY101943.1 *Plantago mauritanica*
AY101948.1 *Plantago nubicola*
KJ579147.1 *Plantago radicata* subsp. *monticola*
KJ579149.1 *Plantago almogravensis*
KR297244.1 *Plantago maritima*
KJ579146.1 *Plantago maritima* subsp. *maritima*
KT724053.1 *Veronicastrum sibiricum*
GQ166789.1 *Peltanthera floribunda*
97
98
98
100
100
55
100
80
70
70
100
100
0.0100

(r)

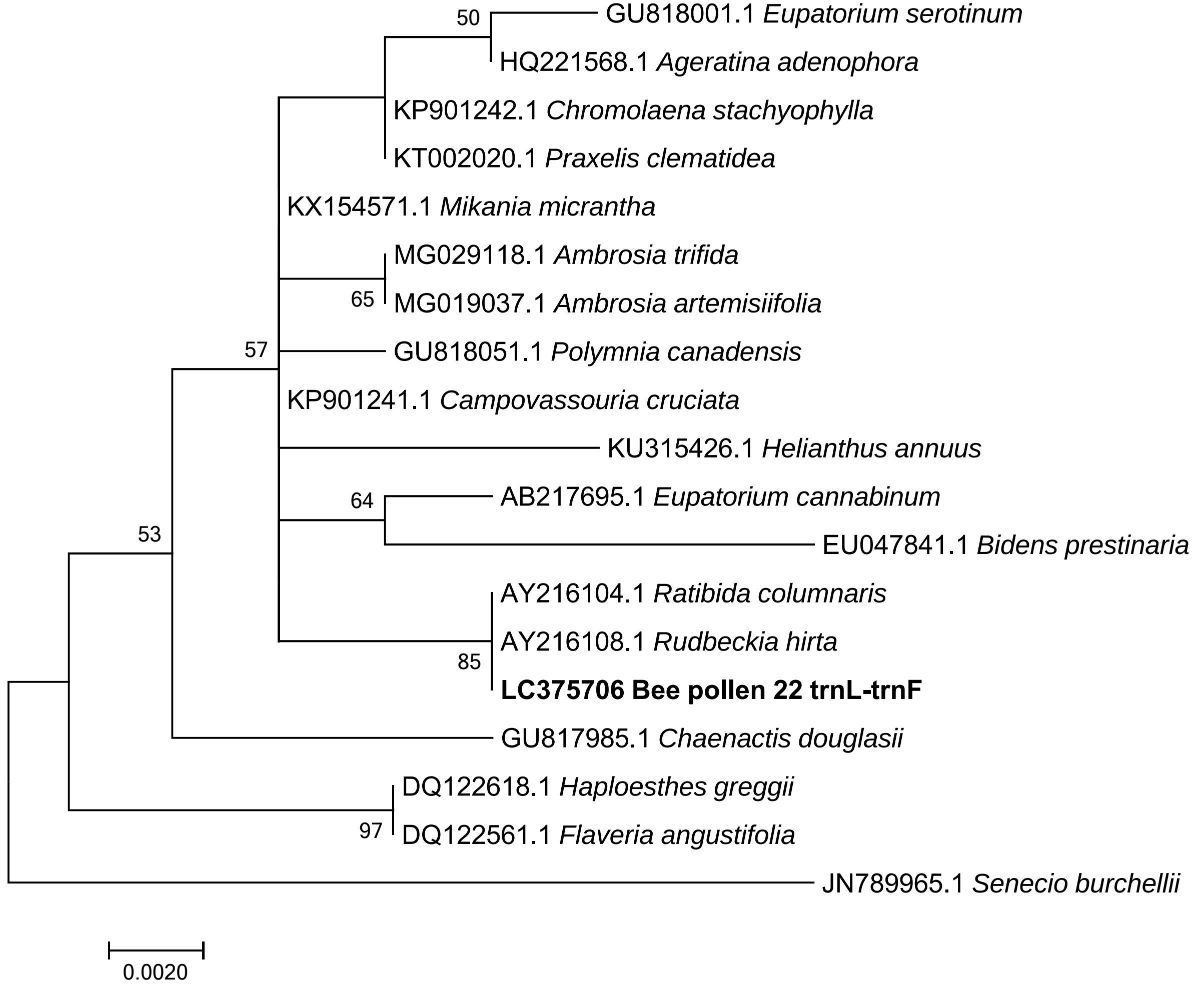

GU818001.1 *Eupatorium serotinum*
HQ221568.1 *Ageratina adenophora*
KP901242.1 *Chromolaena stachyophylla*
KT002020.1 *Praxelis clematidea*
KX154571.1 *Mikania micrantha*
MG029118.1 *Ambrosia trifida*
MG019037.1 *Ambrosia artemisiifolia*
GU818051.1 *Polymnia canadensis*
KP901241.1 *Campovassouria cruciata*
KU315426.1 *Helianthus annuus*
AB217695.1 *Eupatorium cannabinum*
EU047841.1 *Bidens prestinaria*
AY216104.1 *Ratibida columnaris*
AY216108.1 *Rudbeckia hirta*
LC375706 Bee pollen 22 trnL-trnF
GU817985.1 *Chaenactis douglasii*
DQ122618.1 *Haploesthes greggii*
DQ122561.1 *Flaveria angustifolia*
JN789965.1 *Senecio burchellii*
50
65
57
64
53
85
97
0.0020

(s)

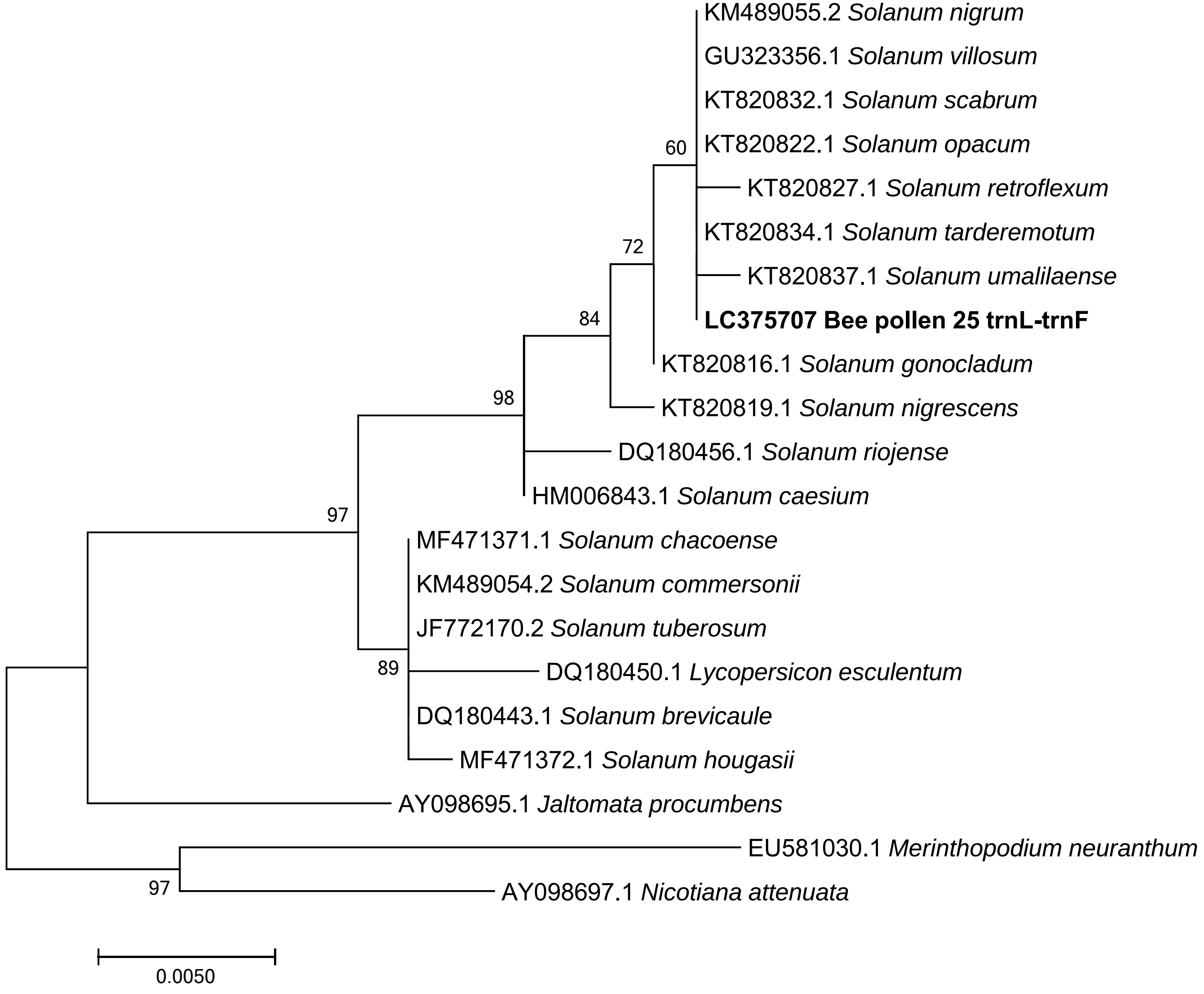
KM489055.2 Solanum nigrum
GU323356.1 Solanum villosum
KT820832.1 Solanum scabrum
KT820822.1 Solanum opacum
KT820827.1 Solanum retroflexum
KT820834.1 Solanum tarderemotum
KT820837.1 Solanum umalilaense
LC375707 Bee pollen 25 trnL-trnF
KT820816.1 Solanum gonocladum
KT820819.1 Solanum nigrescens
DQ180456.1 Solanum riojense
HM006843.1 Solanum caesium
MF471371.1 Solanum chacoense
KM489054.2 Solanum commersonii
JF772170.2 Solanum tuberosum
DQ180450.1 Lycopersicon esculentum
DQ180443.1 Solanum brevicaule
MF471372.1 Solanum hougasii
AY098695.1 Jaltomata procumbens
EU581030.1 Merinthopodium neuranthum
AY098697.1 Nicotiana attenuata
60
72
84
98
97
89
97
0.0050

(t)

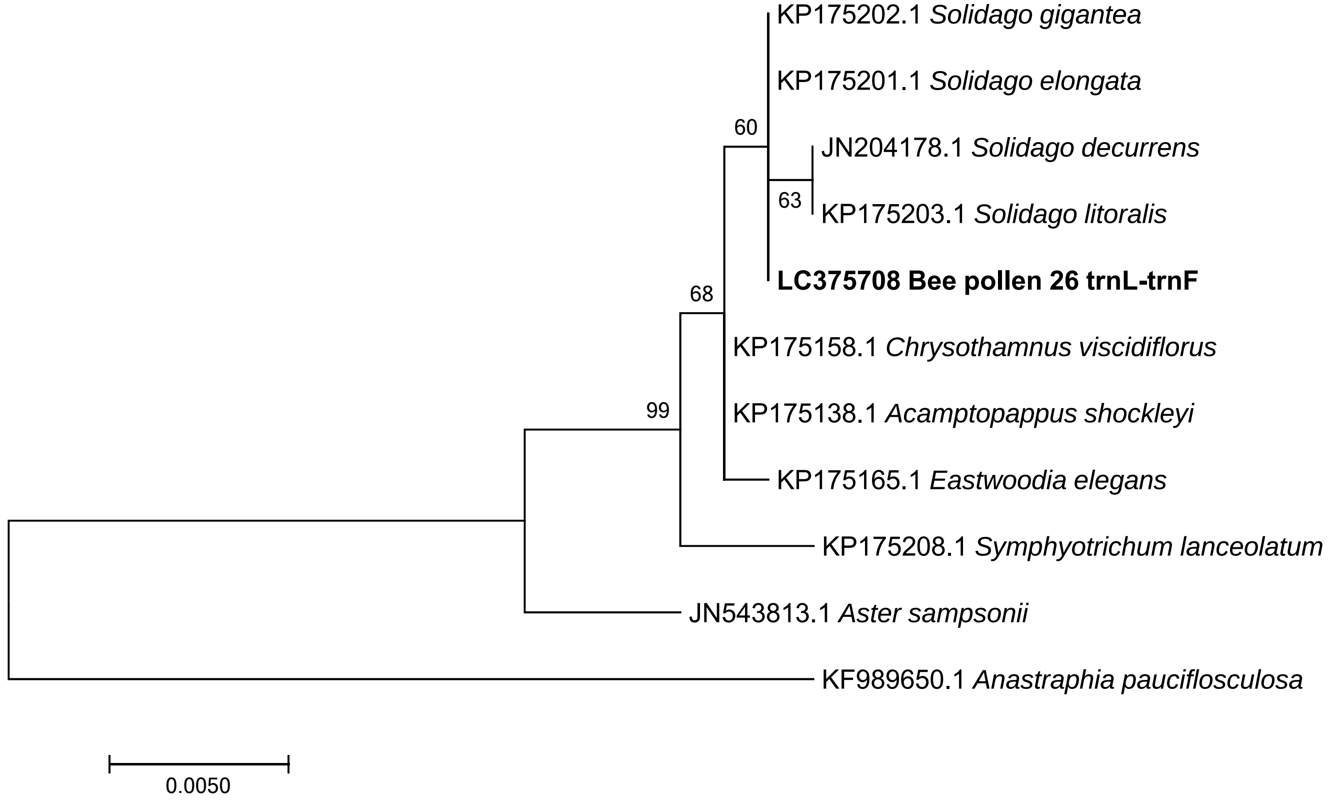
KP175202.1 Solidago gigantea
KP175201.1 Solidago elongata
60
JN204178.1 Solidago decurrens
63
KP175203.1 Solidago litoralis
LC375708 Bee pollen 26 trnL-trnF
68
KP175158.1 Chrysothamnus viscidiflorus
99
KP175138.1 Acamptopappus shockleyi
KP175165.1 Eastwoodia elegans
KP175208.1 Symphyotrichum lanceolatum
JN543813.1 Aster sampsonii
KF989650.1 Anastraphia pauciflosculosa
0.0050

(u)

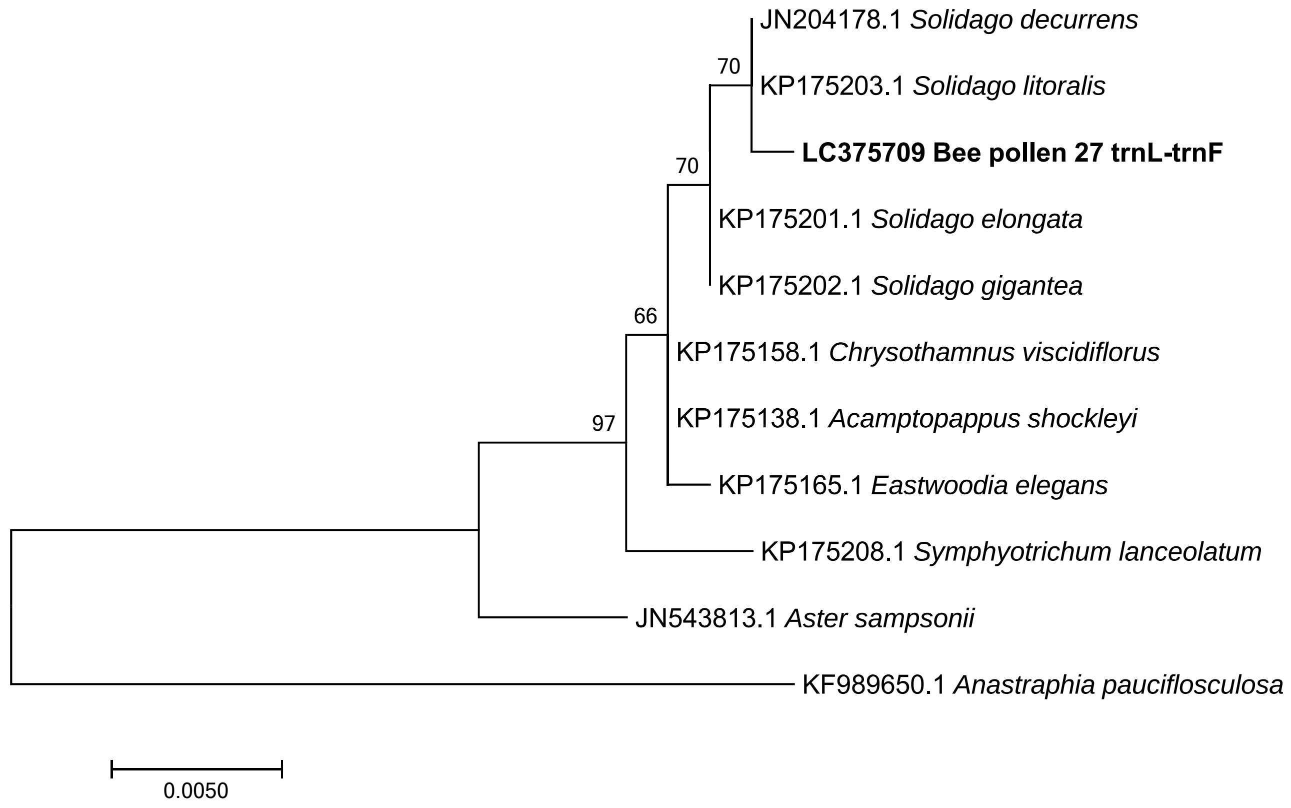

JN204178.1 *Solidago decurrens*
70
KP175203.1 *Solidago litoralis*
LC375709 Bee pollen 27 trnL-trnF
70
KP175201.1 *Solidago elongata*
KP175202.1 *Solidago gigantea*
66
KP175158.1 *Chrysothamnus viscidiflorus*
97
KP175138.1 *Acamptopappus shockleyi*
KP175165.1 *Eastwoodia elegans*
KP175208.1 *Symphyotrichum lanceolatum*
JN543813.1 *Aster sampsonii*
KF989650.1 *Anastraphia pauciflosculosa*
0.0050

(v)

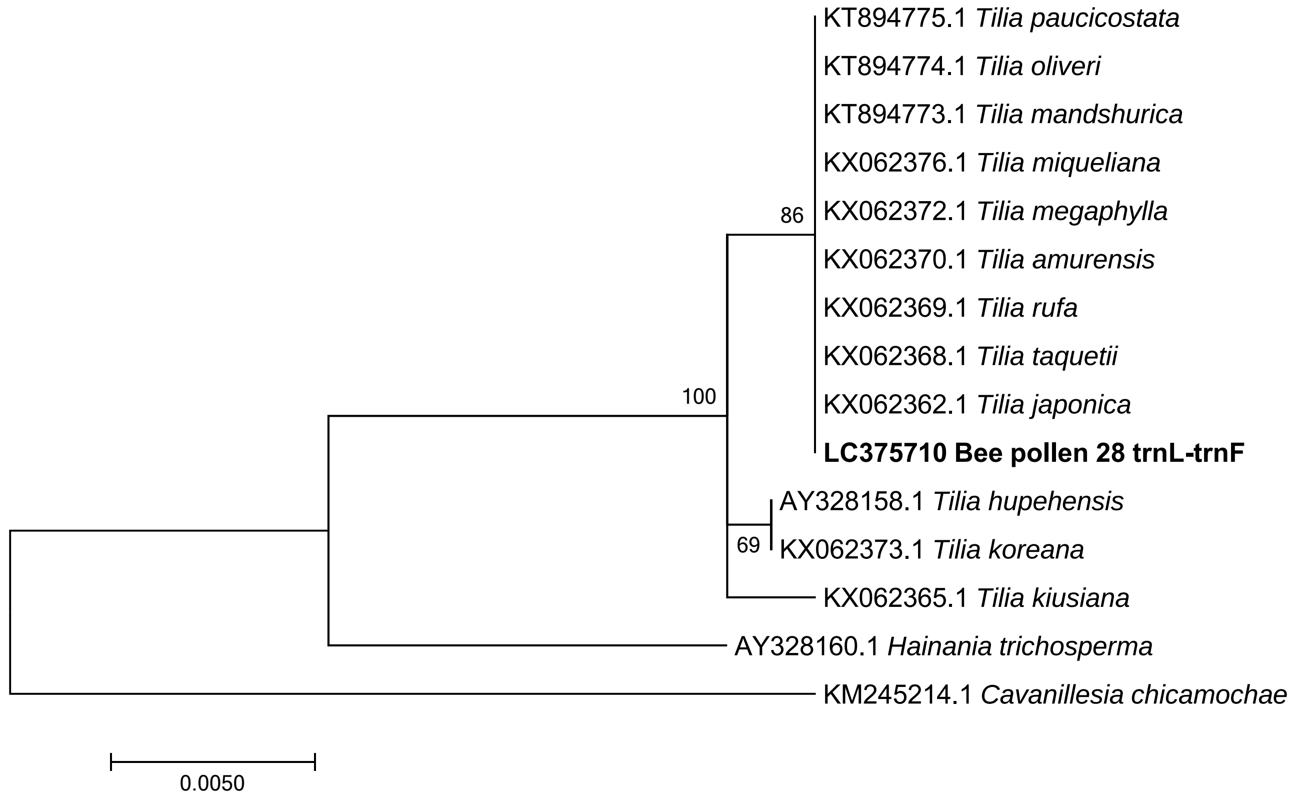

KT894775.1 *Tilia paucicostata*
KT894774.1 *Tilia oliveri*
KT894773.1 *Tilia mandshurica*
KX062376.1 *Tilia miqueliana*
86
KX062372.1 *Tilia megaphylla*
KX062370.1 *Tilia amurensis*
KX062369.1 *Tilia rufa*
KX062368.1 *Tilia taquetii*
100
KX062362.1 *Tilia japonica*
LC375710 Bee pollen 28 trnL-trnF
AY328158.1 *Tilia hupehensis*
69
KX062373.1 *Tilia koreana*
KX062365.1 *Tilia kiusiana*
AY328160.1 *Hainania trichosperma*
KM245214.1 *Cavanillesia chicamochae*
0.0050

(w)

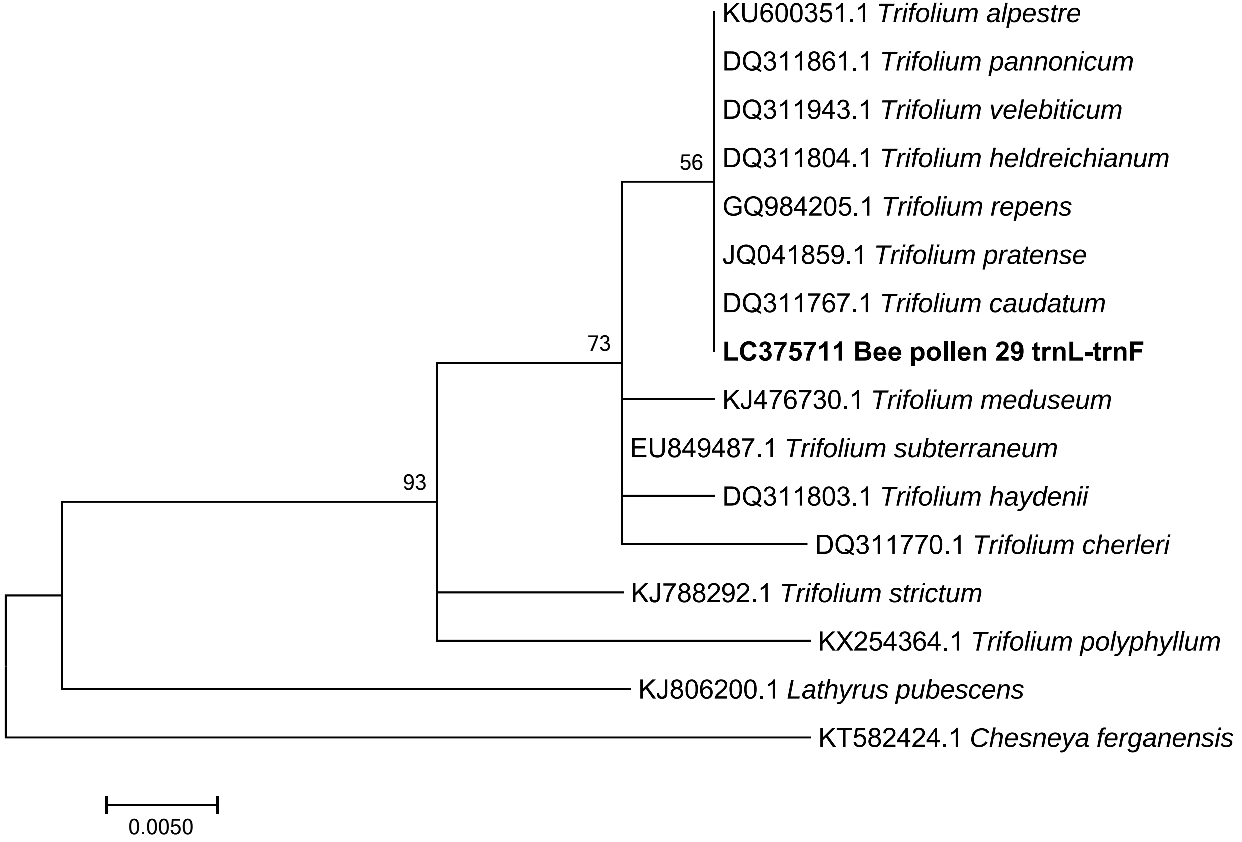
KU600351.1 *Trifolium alpestre*
DQ311861.1 *Trifolium pannonicum*
DQ311943.1 *Trifolium velebiticum*
DQ311804.1 *Trifolium heldreichianum*
GQ984205.1 *Trifolium repens*
JQ041859.1 *Trifolium pratense*
DQ311767.1 *Trifolium caudatum*
LC375711 Bee pollen 29 trnL-trnF
KJ476730.1 *Trifolium meduseum*
EU849487.1 *Trifolium subterraneum*
DQ311803.1 *Trifolium haydenii*
DQ311770.1 *Trifolium cherleri*
KJ788292.1 *Trifolium strictum*
KX254364.1 *Trifolium polyphyllum*
KJ806200.1 *Lathyrus pubescens*
KT582424.1 *Chesneya ferganensis*
56
73
93
0.0050

(x)

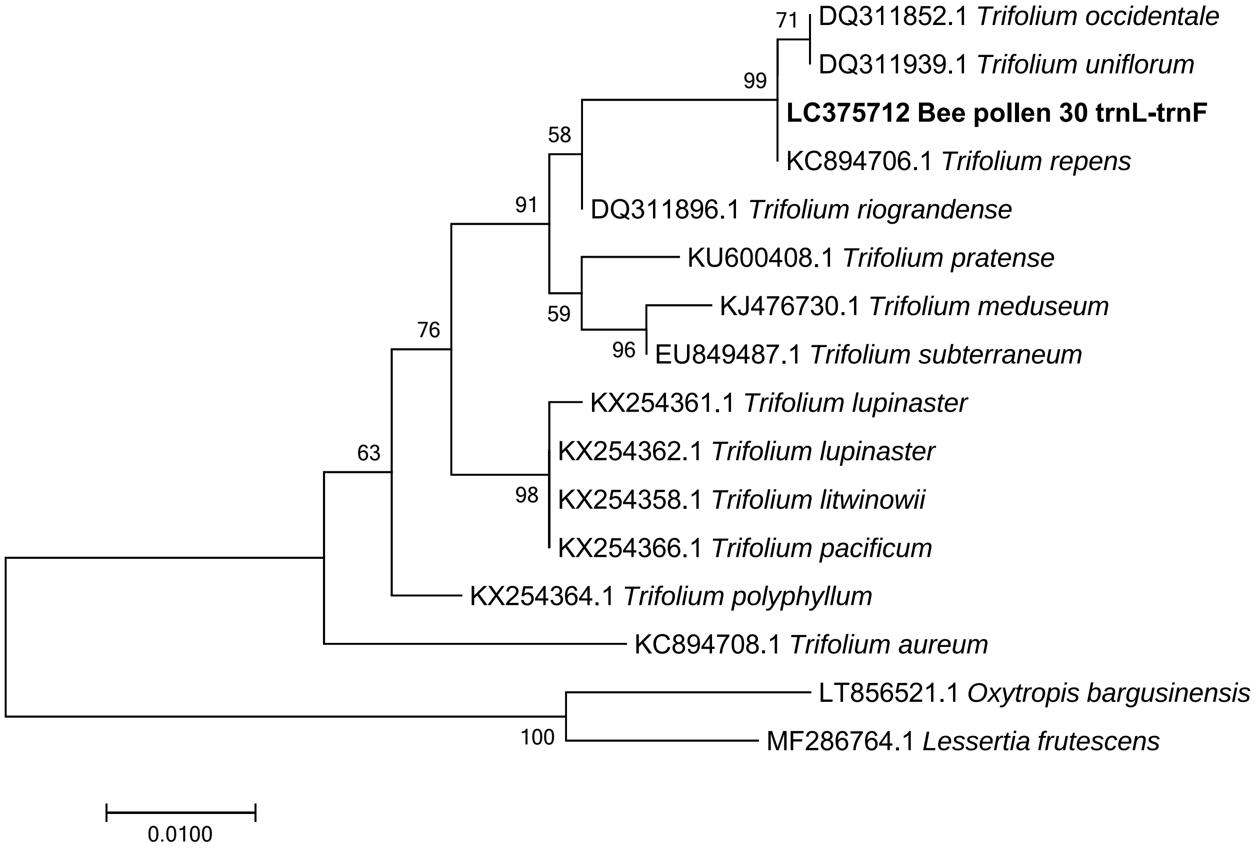
71
DQ311852.1 Trifolium occidentale
DQ311939.1 Trifolium uniflorum
99
LC375712 Bee pollen 30 trnL-trnF
KC894706.1 Trifolium repens
58
DQ311896.1 Trifolium riograndense
91
KU600408.1 Trifolium pratense
KJ476730.1 Trifolium meduseum
59
96
EU849487.1 Trifolium subterraneum
76
KX254361.1 Trifolium lupinaster
KX254362.1 Trifolium lupinaster
63
98
KX254358.1 Trifolium litwinowii
KX254366.1 Trifolium pacificum
KX254364.1 Trifolium polyphyllum
KC894708.1 Trifolium aureum
LT856521.1 Oxytropis bargusinensis
100
MF286764.1 Lessertia frutescens
0.0100

(y)

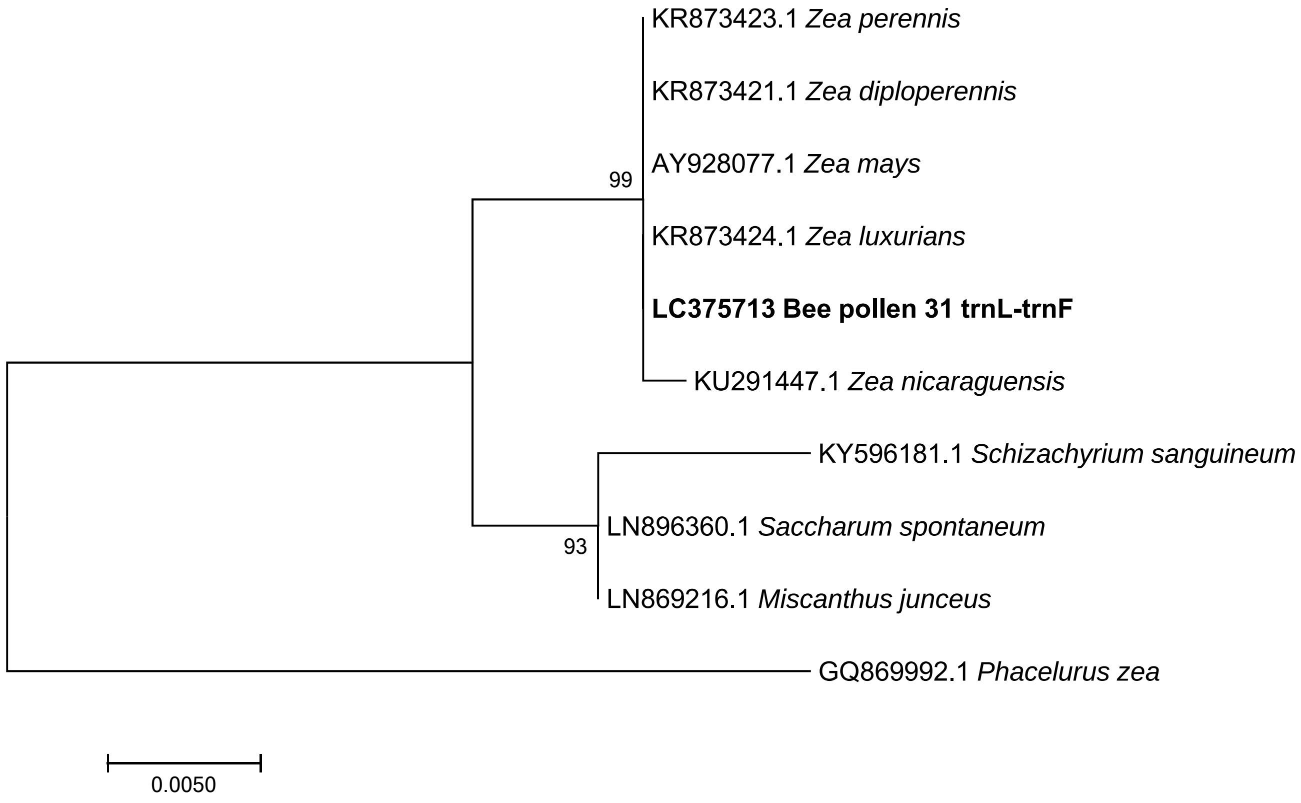
KR873423.1 *Zea perennis*
KR873421.1 *Zea diploperennis*
99
AY928077.1 *Zea mays*
KR873424.1 *Zea luxurians*
LC375713 Bee pollen 31 trnL-trnF
KU291447.1 *Zea nicaraguensis*
KY596181.1 *Schizachyrium sanguineum*
LN896360.1 *Saccharum spontaneum*
93
LN869216.1 *Miscanthus junceus*
GQ869992.1 *Phacelurus zea*
0.0050
