# Supplementary material for: A DNA barcoding method for identifying and quantifying the composition of pollen species collected by European honeybees, Apis mellifera (Hymenoptera: Apidae)
Source: Appl Entomol Zool. 2018 May 16;53(3):353–61. doi: 10.1007/s13355-018-0565-9 (PMC6060998; doi:10.1007/s13355-018-0565-9)
Supplement: Supplementary file 6 — Supplementary material 6 (PDF 221 kb) [file 13355_2018_565_MOESM6_ESM.pdf]

**Fig. S2 Molecular phylogenetic trees of honeybee pollen pellets by maximum likelihood method in ITS2**

The evolutionary history was inferred by using the maximum likelihood method based on the Tamura–Nei model. The tree with the highest log likelihood is shown. The percentage of trees in which the associated taxa clustered together is shown next to the branches. Initial tree(s) for the heuristic search were obtained automatically by applying the Neighbor-Join and BioNJ algorithms to a matrix of pairwise distances estimated using the maximum composite likelihood approach, and then selecting the topology with superior log likelihood value. Each tree is drawn to scale, with branch lengths indicating the number of substitutions per site. The analysis involved 10–26 nucleotide sequences. All positions containing gaps and missing data were eliminated. Evolutionary analyses were conducted in MEGA7. The numbering of honeybee pollen pellets is as follows: (a) No. 1; (b) No. 2; (c) No. 3; (d) No. 4; (e) No. 5; (f) No. 6; (g) No. 7; (h) No. 9; (i) No. 12; (j) No. 13; (k) No. 15; (l) No. 16; (m) No. 19; (n) No. 20; (o) No. 21; (p) No. 22; (q) No. 23; (r) No. 24; (s) No. 25; (t) No. 26; (u) No. 27; (v) No. 28; (w) No. 29; (x) No. 30.

(a)

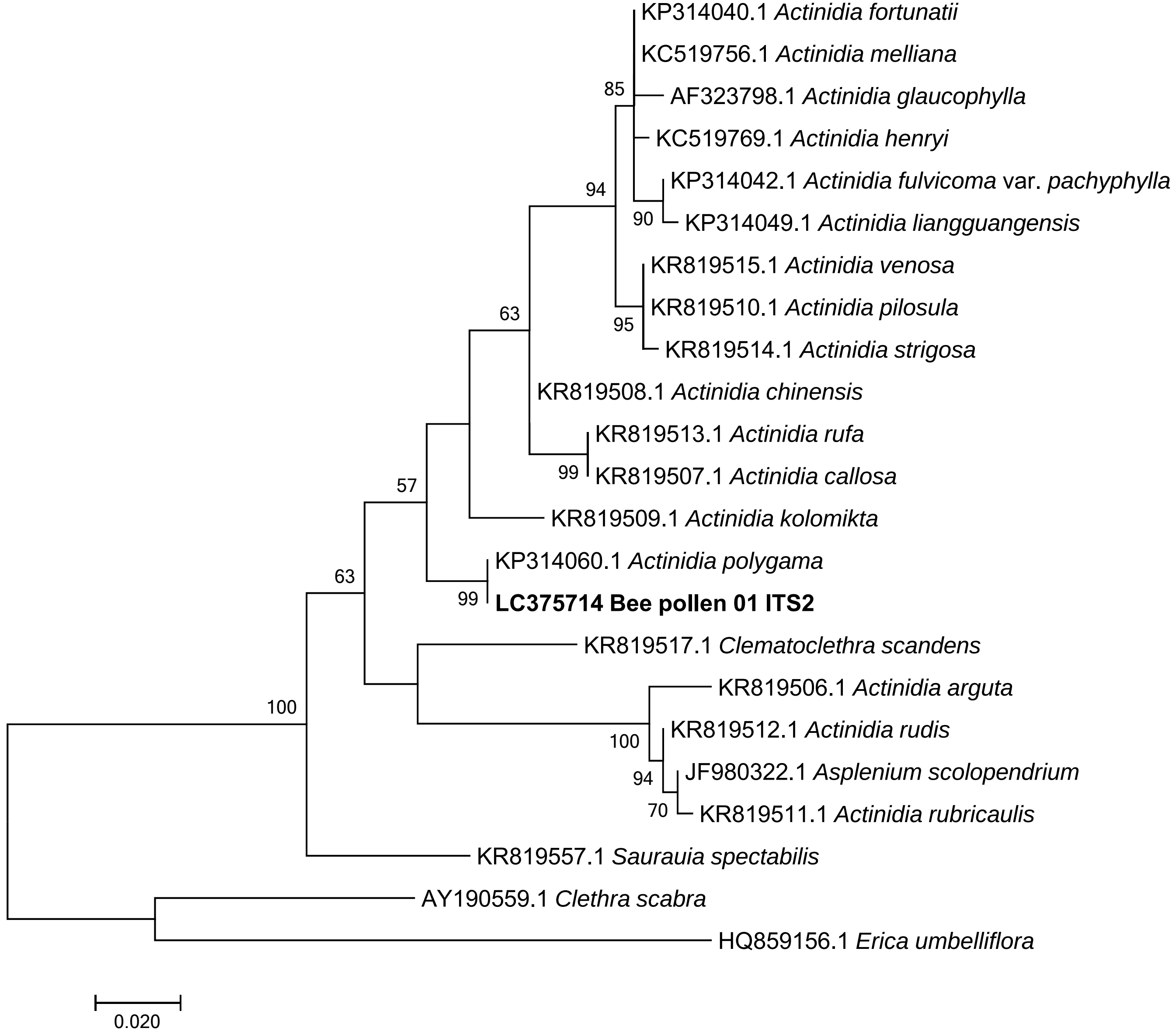
KP314040.1 *Actinidia fortunatii*
KC519756.1 *Actinidia melliana*
AF323798.1 *Actinidia glaucophylla*
KC519769.1 *Actinidia henryi*
KP314042.1 *Actinidia fulvicoma* var. *pachyphylla*
KP314049.1 *Actinidia liangguangensis*
KR819515.1 *Actinidia venosa*
KR819510.1 *Actinidia pilosula*
KR819514.1 *Actinidia strigosa*
KR819508.1 *Actinidia chinensis*
KR819513.1 *Actinidia rufa*
KR819507.1 *Actinidia callosa*
KR819509.1 *Actinidia kolomikta*
KP314060.1 *Actinidia polygama*
**LC375714 Bee pollen 01 ITS2**
KR819517.1 *Clematoclethra scandens*
KR819506.1 *Actinidia arguta*
KR819512.1 *Actinidia rudis*
JF980322.1 *Asplenium scolopendrium*
KR819511.1 *Actinidia rubricaulis*
KR819557.1 *Saurauia spectabilis*
AY190559.1 *Clethra scabra*
HQ859156.1 *Erica umbelliflora*
85
94
90
95
63
99
57
99
63
100
100
94
70
0.020

(b)

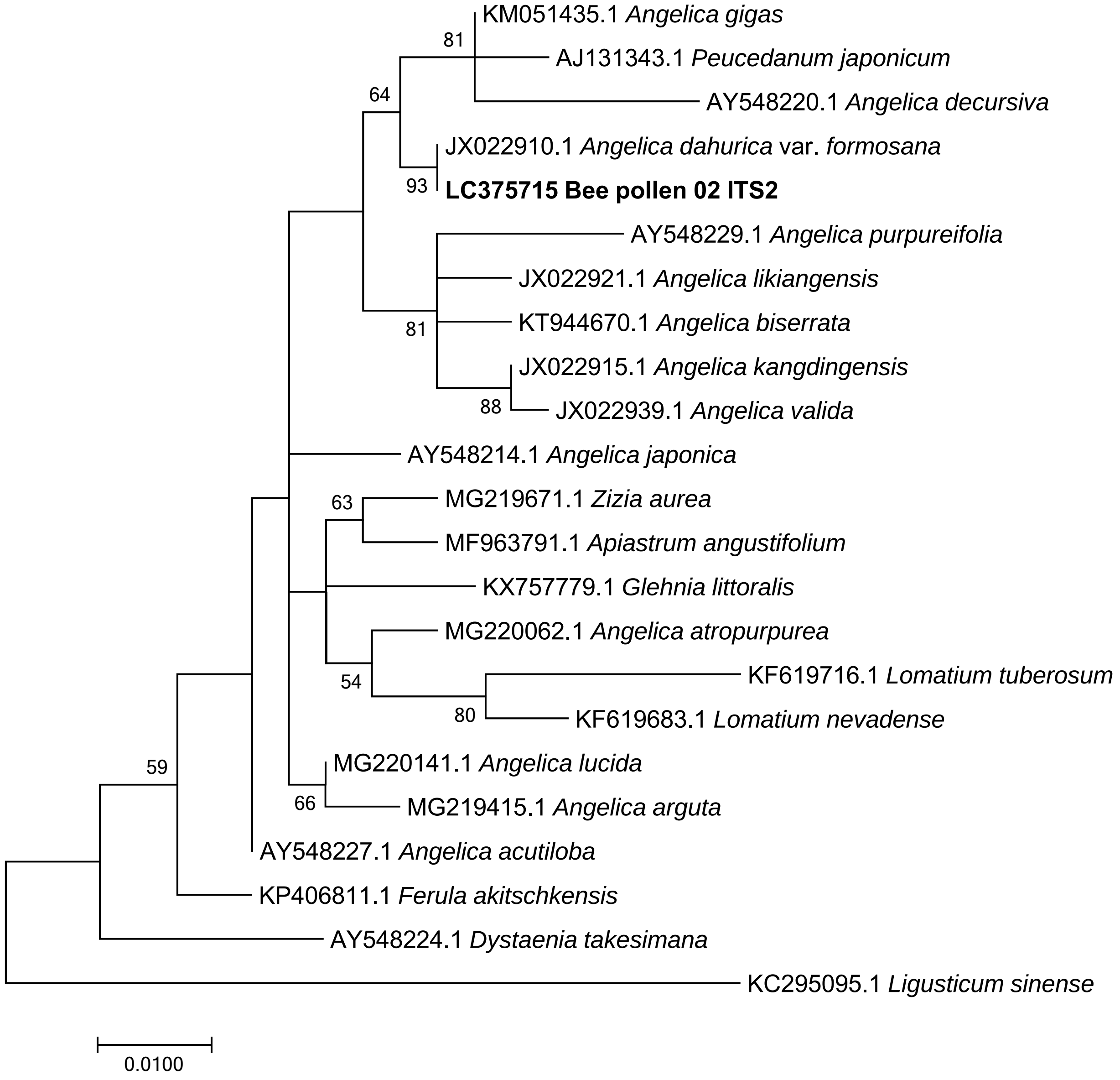
KM051435.1 Angelica gigas
AJ131343.1 Peucedanum japonicum
AY548220.1 Angelica decursiva
JX022910.1 Angelica dahurica var. formosana
LC375715 Bee pollen 02 ITS2
AY548229.1 Angelica purpureifolia
JX022921.1 Angelica likiangensis
KT944670.1 Angelica biserrata
JX022915.1 Angelica kangdingensis
JX022939.1 Angelica valida
AY548214.1 Angelica japonica
MG219671.1 Zizia aurea
MF963791.1 Apiastrum angustifolium
KX757779.1 Glehnia littoralis
MG220062.1 Angelica atropurpurea
KF619716.1 Lomatium tuberosum
KF619683.1 Lomatium nevadense
MG220141.1 Angelica lucida
MG219415.1 Angelica arguta
AY548227.1 Angelica acutiloba
KP406811.1 Ferula akitschkensis
AY548224.1 Dystaenia takesimana
KC295095.1 Ligusticum sinense
81
64
93
81
88
63
54
80
59
66
0.0100

(c)

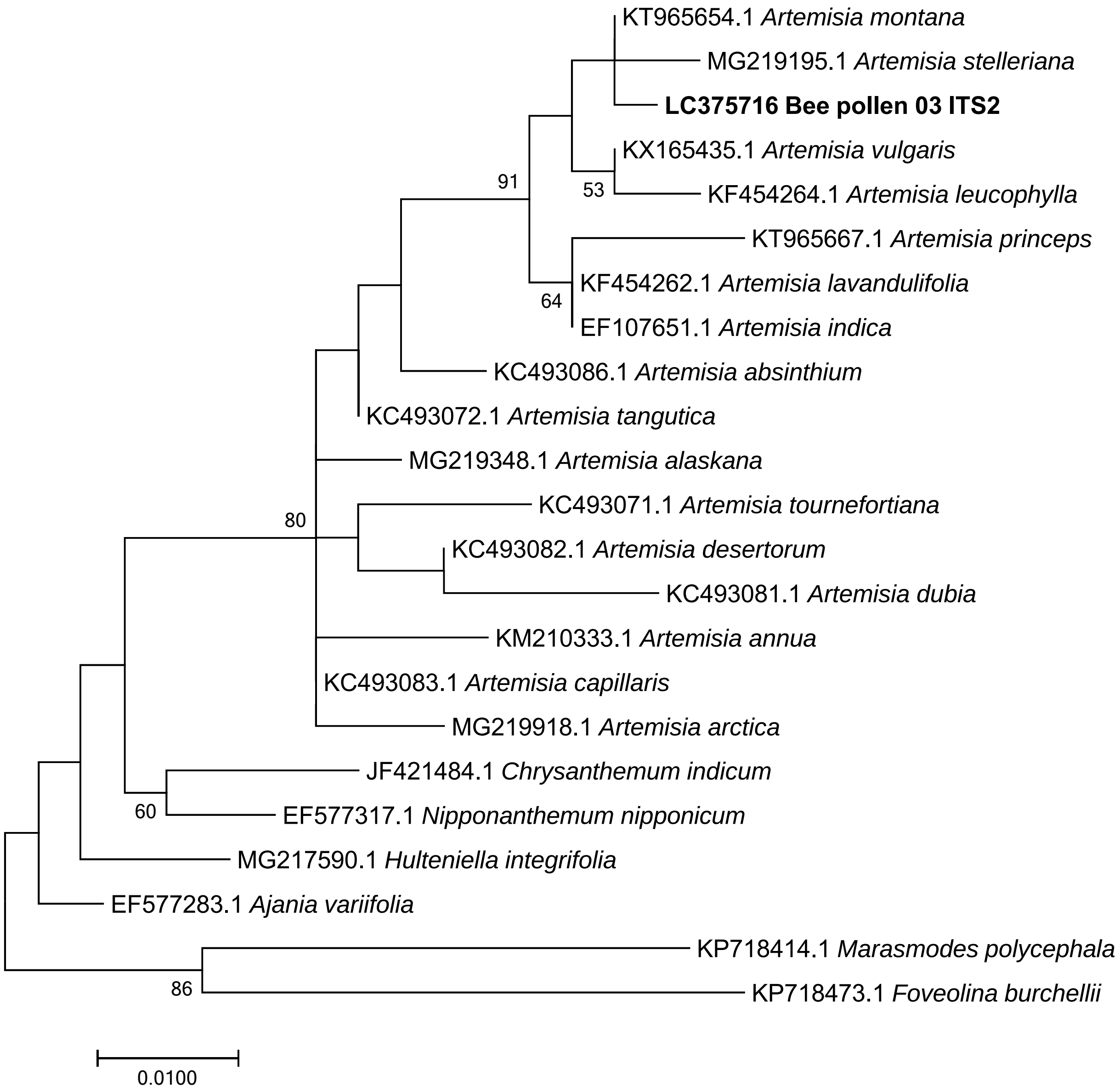
KT965654.1 Artemisia montana
MG219195.1 Artemisia stelleriana
LC375716 Bee pollen 03 ITS2
KX165435.1 Artemisia vulgaris
KF454264.1 Artemisia leucophylla
KT965667.1 Artemisia princeps
KF454262.1 Artemisia lavandulifolia
EF107651.1 Artemisia indica
KC493086.1 Artemisia absinthium
KC493072.1 Artemisia tangutica
MG219348.1 Artemisia alaskana
KC493071.1 Artemisia tournefortiana
KC493082.1 Artemisia desertorum
KC493081.1 Artemisia dubia
KM210333.1 Artemisia annua
KC493083.1 Artemisia capillaris
MG219918.1 Artemisia arctica
JF421484.1 Chrysanthemum indicum
EF577317.1 Nipponanthemum nipponicum
MG217590.1 Hulteniella integrifolia
EF577283.1 Ajania variifolia
KP718414.1 Marasmodes polycephala
KP718473.1 Foveolina burchellii
91
53
64
80
60
86
0.0100

(d)

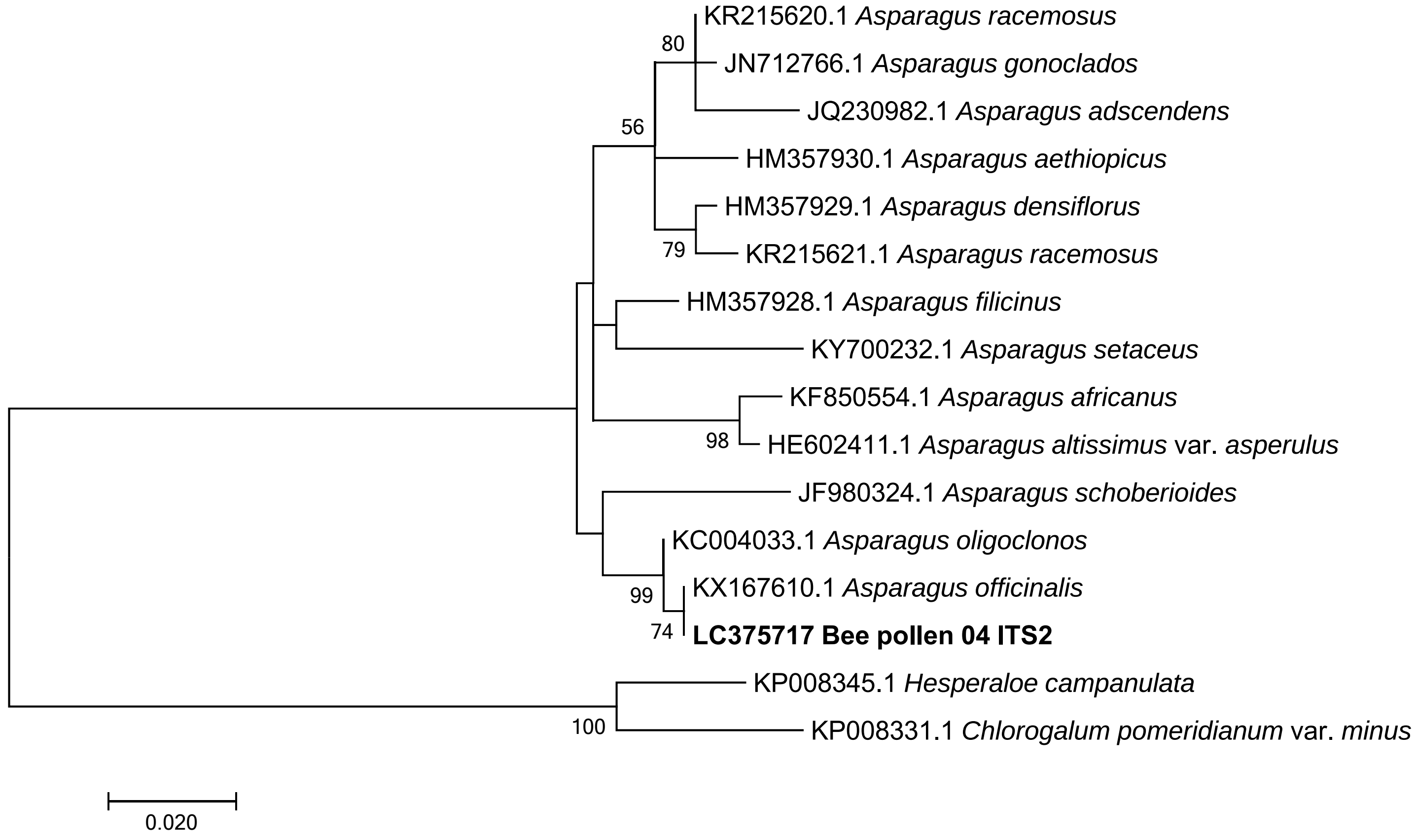

KR215620.1 Asparagus racemosus
JN712766.1 Asparagus gonoclados
JQ230982.1 Asparagus adscendens
HM357930.1 Asparagus aethiopicus
HM357929.1 Asparagus densiflorus
KR215621.1 Asparagus racemosus
HM357928.1 Asparagus filicinus
KY700232.1 Asparagus setaceus
KF850554.1 Asparagus africanus
HE602411.1 Asparagus altissimus var. asperulus
JF980324.1 Asparagus schoberioides
KC004033.1 Asparagus oligoclonos
KX167610.1 Asparagus officinalis
LC375717 Bee pollen 04 ITS2
KP008345.1 Hesperaloe campanulata
KP008331.1 Chlorogalum pomeridianum var. minus
80
56
79
98
99
74
100
0.020

(e)

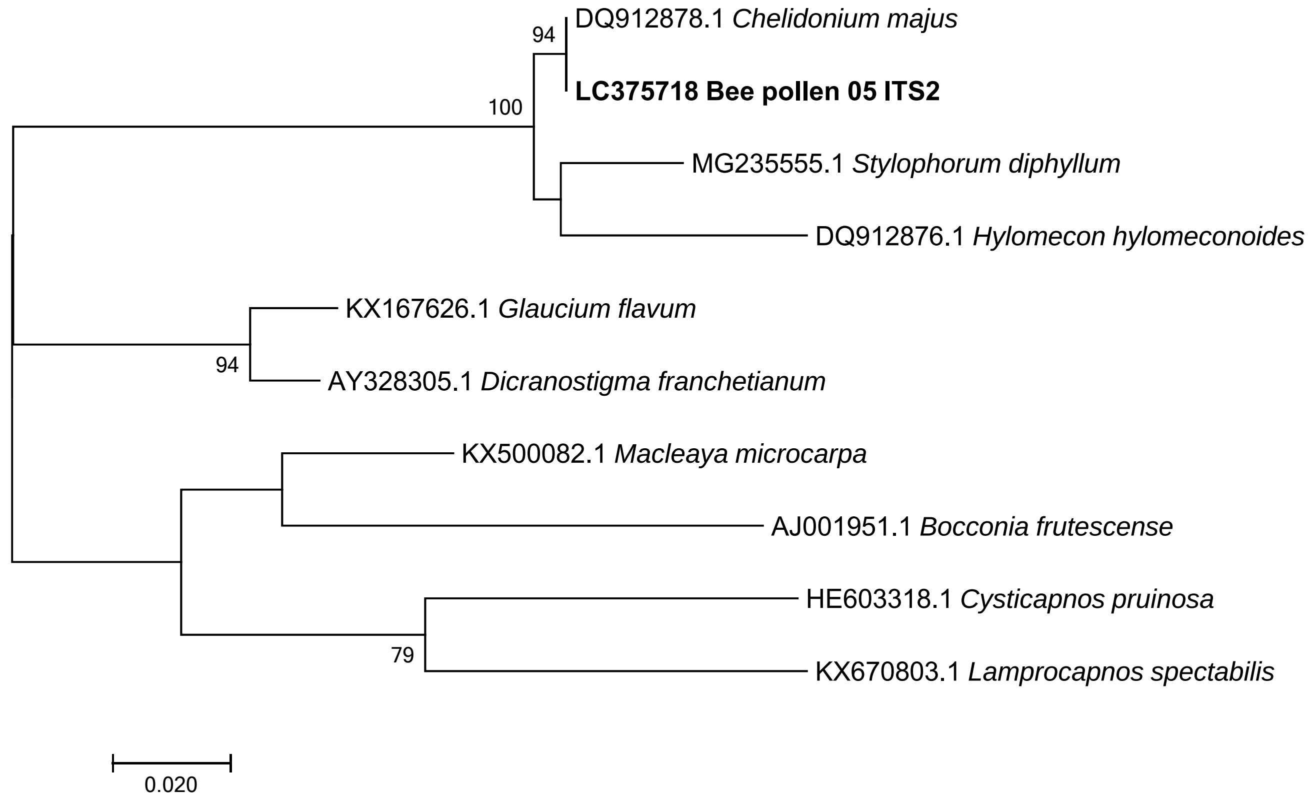
DQ912878.1 *Chelidonium majus*
94
**LC375718 Bee pollen 05 ITS2**
100
MG235555.1 *Stylophorum diphyllum*
DQ912876.1 *Hylomecon hylomeconoides*
KX167626.1 *Glaucium flavum*
94
AY328305.1 *Dicranostigma franchetianum*
KX500082.1 *Macleaya microcarpa*
AJ001951.1 *Bocconia frutescense*
HE603318.1 *Cysticapnos pruinosa*
79
KX670803.1 *Lamprocapnos spectabilis*
0.020

(f)

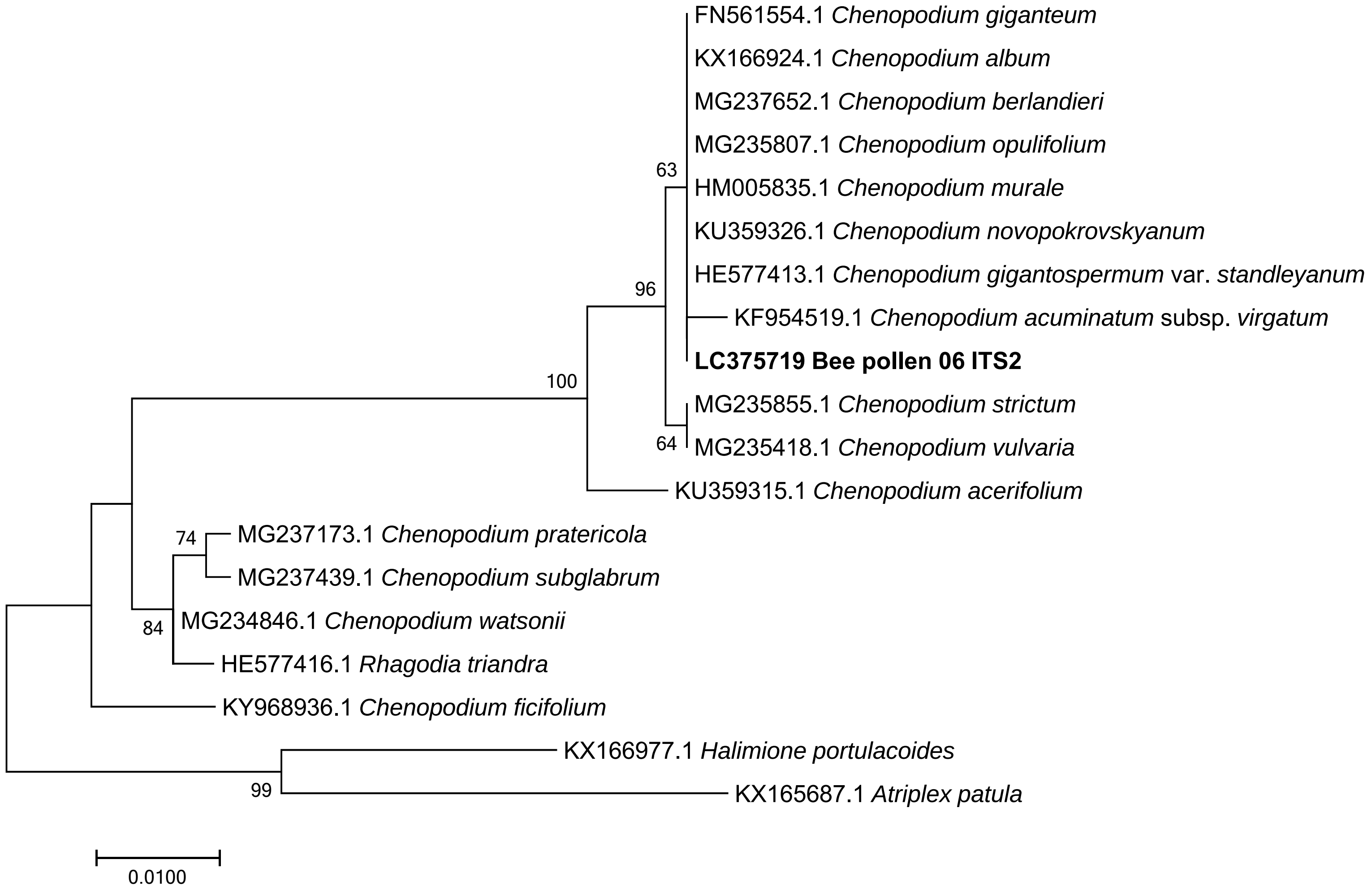

FN561554.1 *Chenopodium giganteum*
KX166924.1 *Chenopodium album*
MG237652.1 *Chenopodium berlandieri*
MG235807.1 *Chenopodium opulifolium*
HM005835.1 *Chenopodium murale*
KU359326.1 *Chenopodium novopokrovskyanum*
HE577413.1 *Chenopodium gigantospermum* var. *standleyanum*
KF954519.1 *Chenopodium acuminatum* subsp. *virgatum*
**LC375719 Bee pollen 06 ITS2**
MG235855.1 *Chenopodium strictum*
MG235418.1 *Chenopodium vulvaria*
KU359315.1 *Chenopodium acerifolium*
MG237173.1 *Chenopodium pratericola*
MG237439.1 *Chenopodium subglabrum*
MG234846.1 *Chenopodium watsonii*
HE577416.1 *Rhagodia triandra*
KY968936.1 *Chenopodium ficifolium*
KX166977.1 *Halimione portulacoides*
KX165687.1 *Atriplex patula*
63
96
100
64
74
84
99
0.0100

(g)

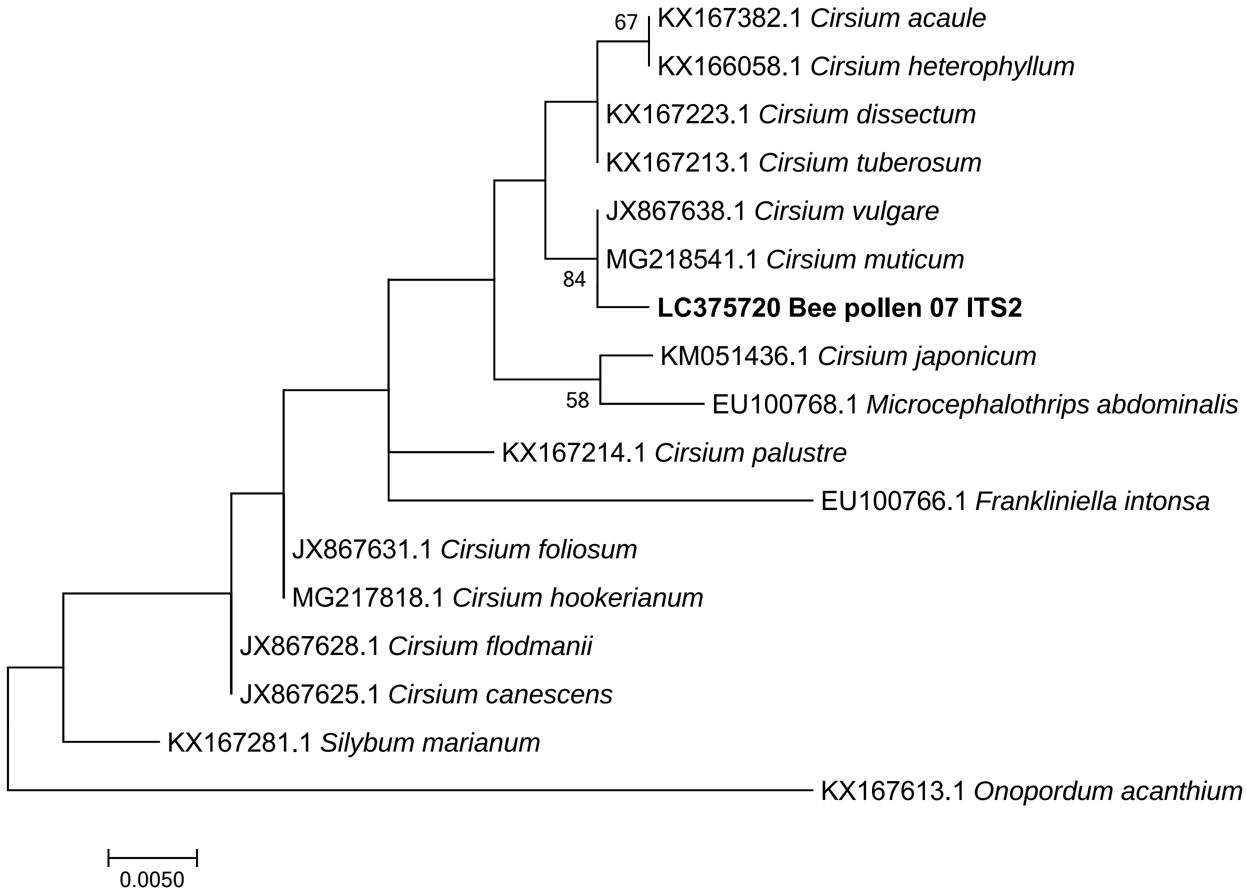
67
KX167382.1 Cirsium acaule
KX166058.1 Cirsium heterophyllum
KX167223.1 Cirsium dissectum
KX167213.1 Cirsium tuberosum
JX867638.1 Cirsium vulgare
MG218541.1 Cirsium muticum
84
LC375720 Bee pollen 07 ITS2
KM051436.1 Cirsium japonicum
58
EU100768.1 Microcephalothrips abdominalis
KX167214.1 Cirsium palustre
EU100766.1 Frankliniella intonsa
JX867631.1 Cirsium foliosum
MG217818.1 Cirsium hookerianum
JX867628.1 Cirsium flodmanii
JX867625.1 Cirsium canescens
KX167281.1 Silybum marianum
KX167613.1 Onopordum acanthium
0.0050

(h)

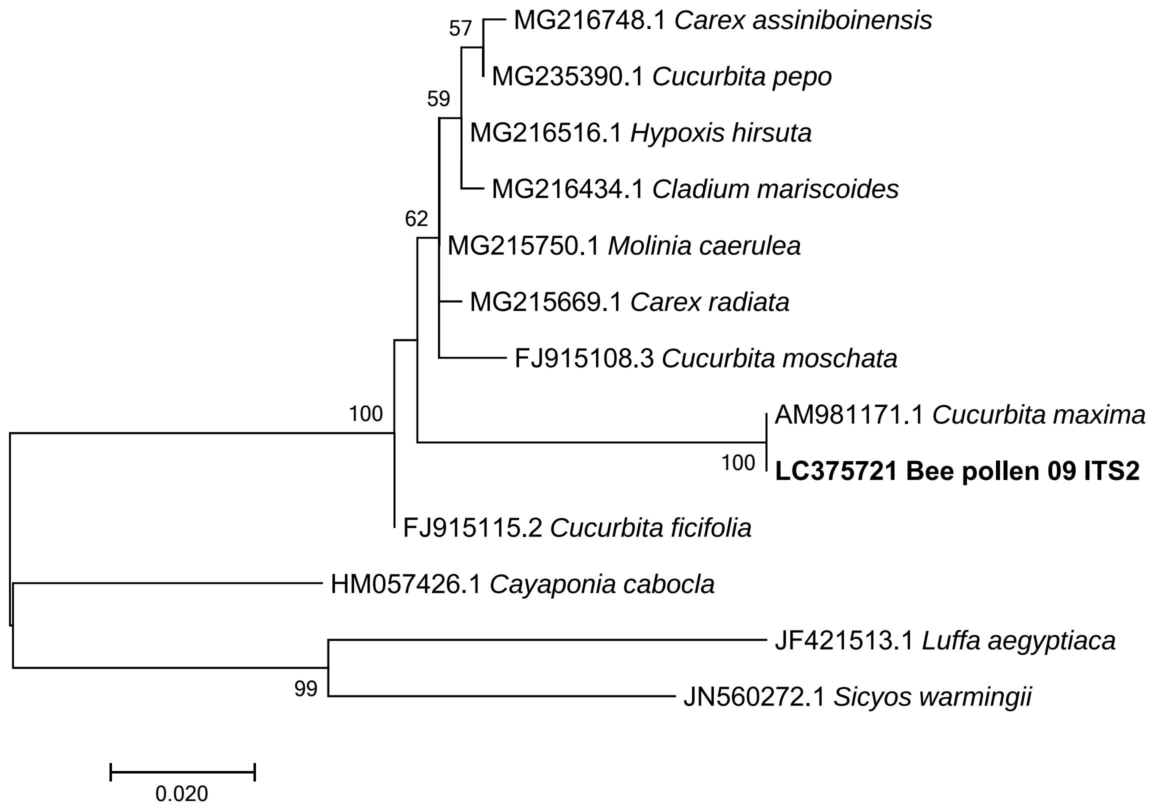
57
MG216748.1 Carex assiniboinensis
MG235390.1 Cucurbita pepo
59
MG216516.1 Hypoxis hirsuta
MG216434.1 Cladium mariscoides
62
MG215750.1 Molinia caerulea
MG215669.1 Carex radiata
FJ915108.3 Cucurbita moschata
100
AM981171.1 Cucurbita maxima
100
LC375721 Bee pollen 09 ITS2
FJ915115.2 Cucurbita ficifolia
HM057426.1 Cayaponia cabocla
JF421513.1 Luffa aegyptiaca
99
JN560272.1 Sicyos warmingii
0.020

(i)

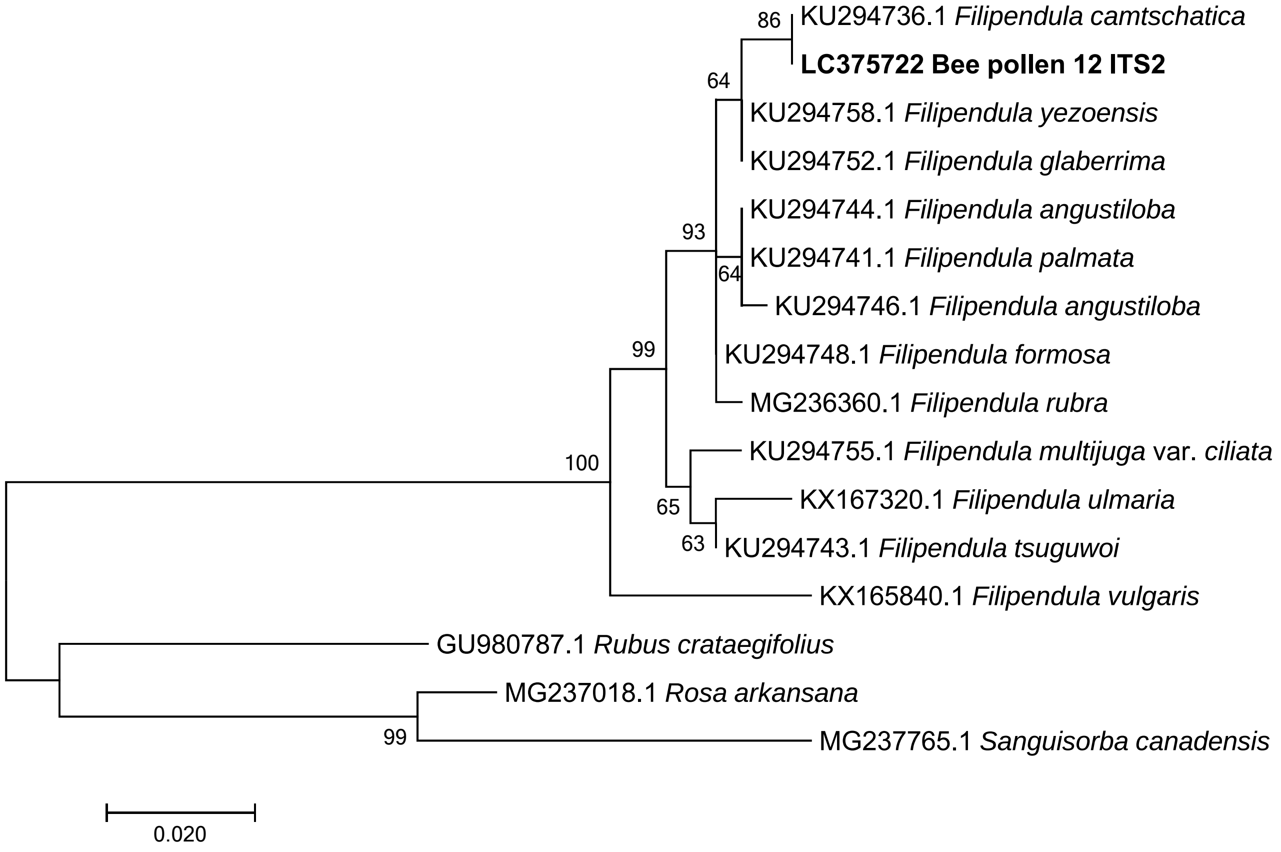

86
KU294736.1 Filipendula camtschatica
LC375722 Bee pollen 12 ITS2
64
KU294758.1 Filipendula yezoensis
KU294752.1 Filipendula glaberrima
KU294744.1 Filipendula angustiloba
93
KU294741.1 Filipendula palmata
64
KU294746.1 Filipendula angustiloba
99
KU294748.1 Filipendula formosa
MG236360.1 Filipendula rubra
KU294755.1 Filipendula multijuga var. ciliata
100
KX167320.1 Filipendula ulmaria
65
63
KU294743.1 Filipendula tsuguwoi
KX165840.1 Filipendula vulgaris
GU980787.1 Rubus crataegifolius
MG237018.1 Rosa arkansana
99
MG237765.1 Sanguisorba canadensis
0.020

(j)

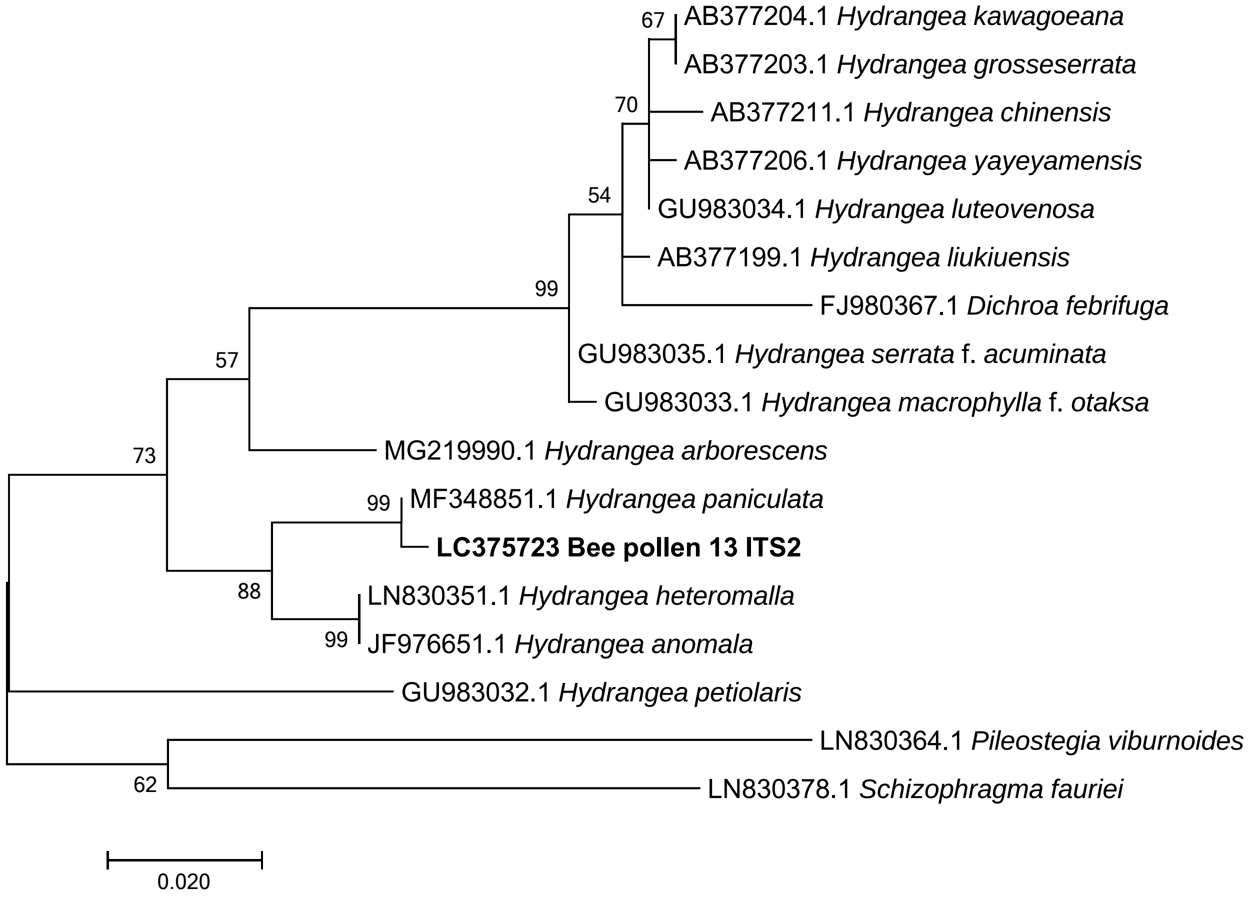

67
AB377204.1 Hydrangea kawagoeana
AB377203.1 Hydrangea grosseserrata
70
AB377211.1 Hydrangea chinensis
AB377206.1 Hydrangea yayeyamensis
54
GU983034.1 Hydrangea luteovenosa
AB377199.1 Hydrangea liukiuensis
99
FJ980367.1 Dichroa febrifuga
57
GU983035.1 Hydrangea serrata f. acuminata
GU983033.1 Hydrangea macrophylla f. otaksa
73
MG219990.1 Hydrangea arborescens
99
MF348851.1 Hydrangea paniculata
LC375723 Bee pollen 13 ITS2
88
LN830351.1 Hydrangea heteromalla
99
JF976651.1 Hydrangea anomala
GU983032.1 Hydrangea petiolaris
LN830364.1 Pileostegia viburnoides
62
LN830378.1 Schizophragma fauriei
0.020

(k)

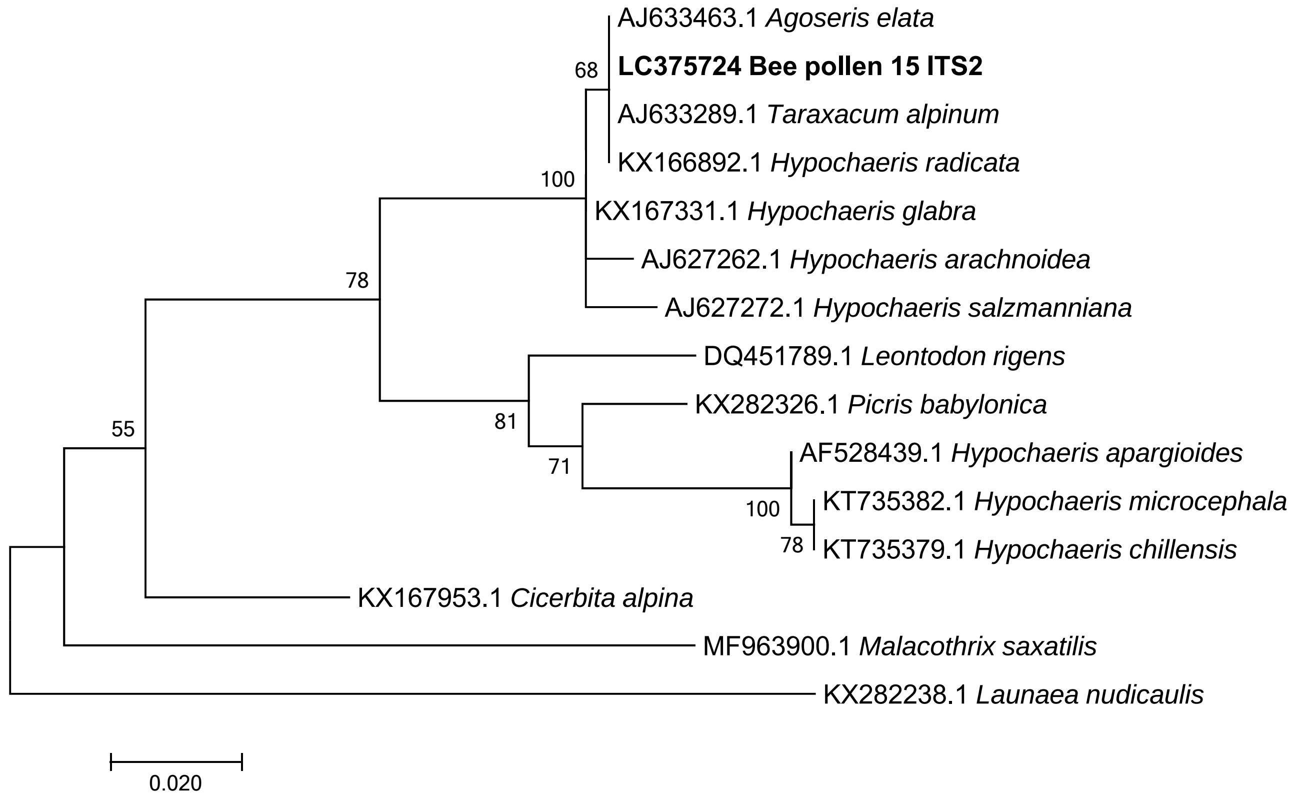
AJ633463.1 *Agoseris elata*
**LC375724 Bee pollen 15 ITS2**
AJ633289.1 *Taraxacum alpinum*
KX166892.1 *Hypochaeris radicata*
KX167331.1 *Hypochaeris glabra*
AJ627262.1 *Hypochaeris arachnoidea*
AJ627272.1 *Hypochaeris salzmanniana*
DQ451789.1 *Leontodon rigens*
KX282326.1 *Picris babylonica*
AF528439.1 *Hypochaeris apargioides*
KT735382.1 *Hypochaeris microcephala*
KT735379.1 *Hypochaeris chillensis*
KX167953.1 *Cicerbita alpina*
MF963900.1 *Malacothrix saxatilis*
KX282238.1 *Launaea nudicaulis*
68
100
78
81
71
55
100
78
0.020

(I)

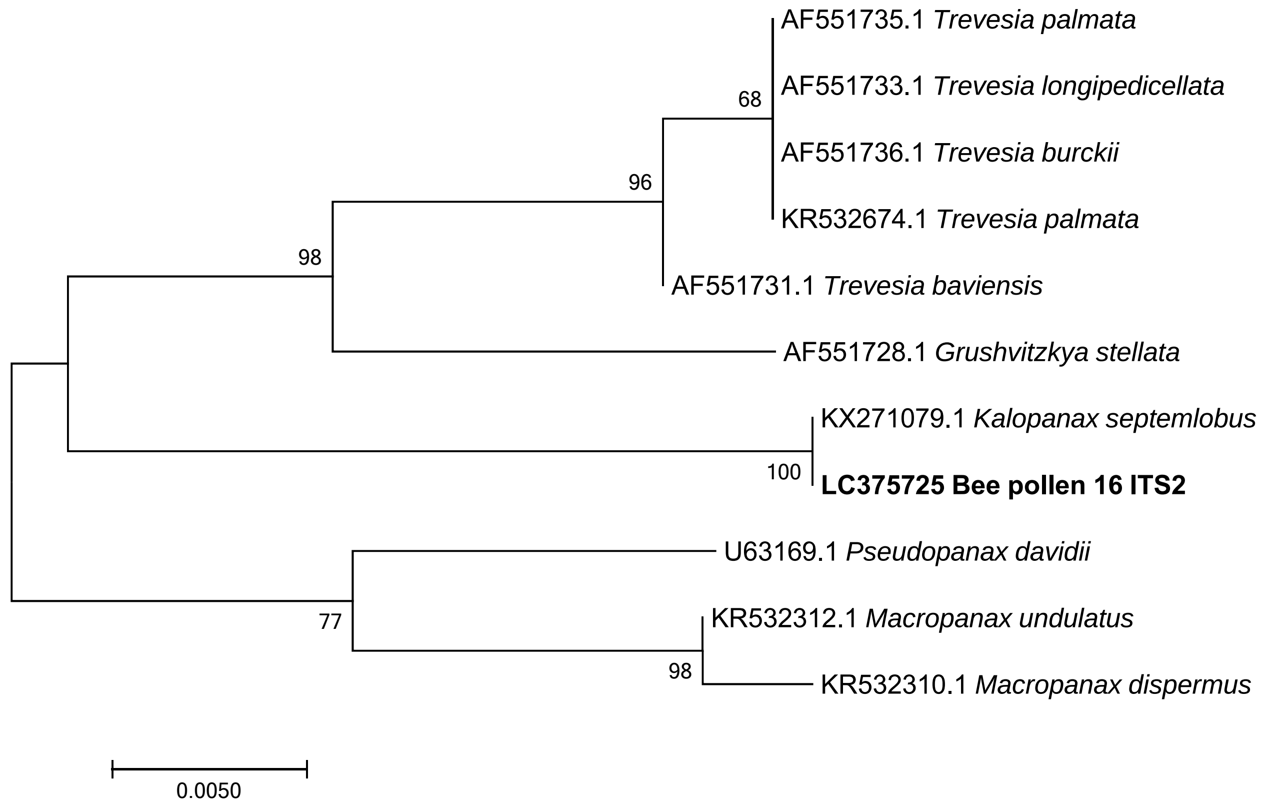
AF551735.1 Trevesia palmata
AF551733.1 Trevesia longipedicellata
AF551736.1 Trevesia burckii
KR532674.1 Trevesia palmata
AF551731.1 Trevesia baviensis
AF551728.1 Grushvitzkya stellata
KX271079.1 Kalopanax septemlobus
LC375725 Bee pollen 16 ITS2
U63169.1 Pseudopanax davidii
KR532312.1 Macropanax undulatus
KR532310.1 Macropanax dispermus
68
96
98
100
77
98
0.0050

(m)

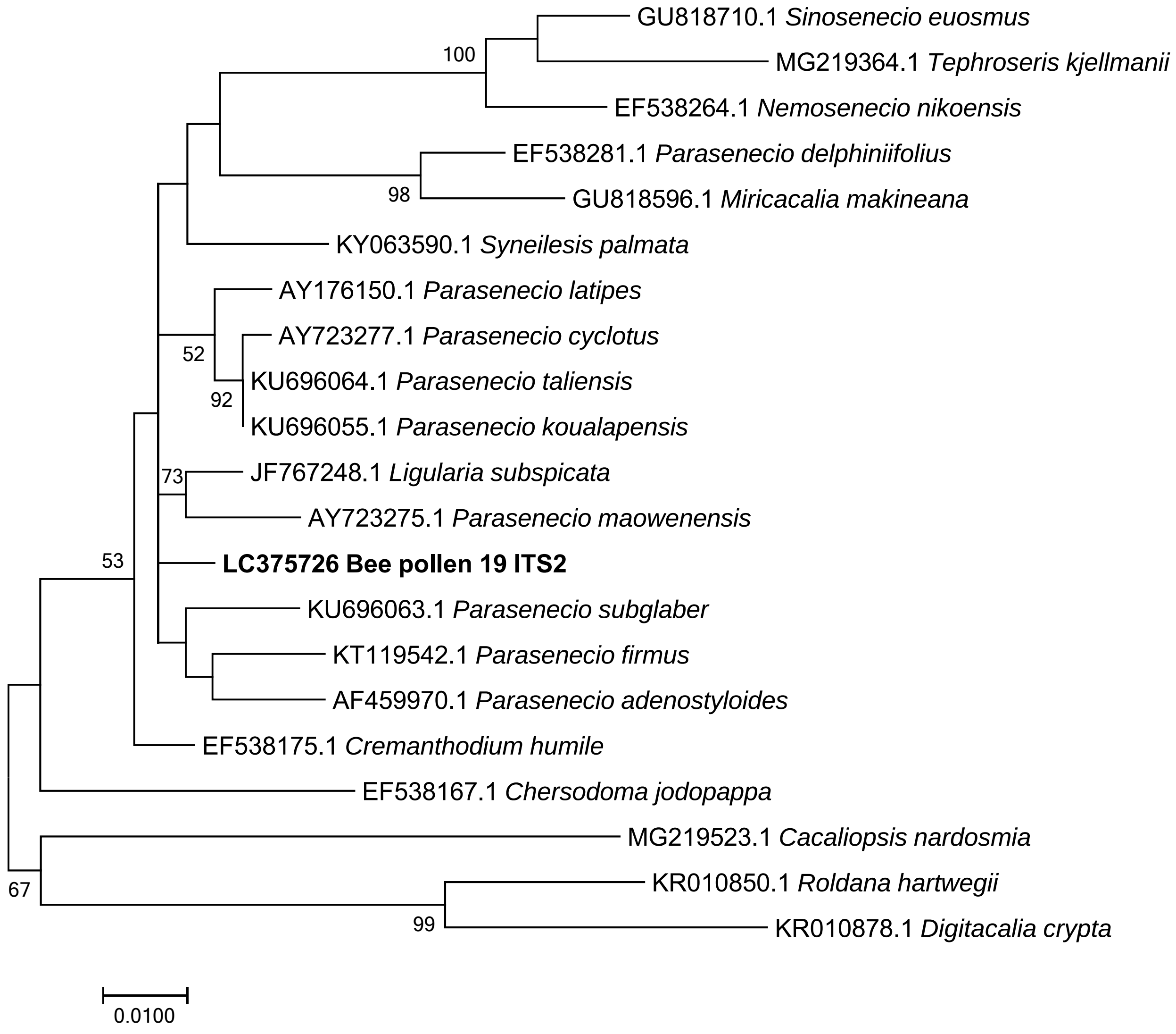
GU818710.1 Sinosenecio euosmus
MG219364.1 Tephroseris kjellmanii
EF538264.1 Nemosenecio nikoensis
EF538281.1 Parasenecio delphiniifolius
GU818596.1 Miricacalia makineana
KY063590.1 Syneilesis palmata
AY176150.1 Parasenecio latipes
AY723277.1 Parasenecio cyclotus
KU696064.1 Parasenecio taliensis
KU696055.1 Parasenecio koualapensis
JF767248.1 Ligularia subspicata
AY723275.1 Parasenecio maowenensis
LC375726 Bee pollen 19 ITS2
KU696063.1 Parasenecio subglaber
KT119542.1 Parasenecio firmus
AF459970.1 Parasenecio adenostyloides
EF538175.1 Cremanthodium humile
EF538167.1 Chersodoma jodopappa
MG219523.1 Cacaliopsis nardosmia
KR010850.1 Roldana hartwegii
KR010878.1 Digitacalia crypta
100
98
52
92
73
53
67
99
0.0100

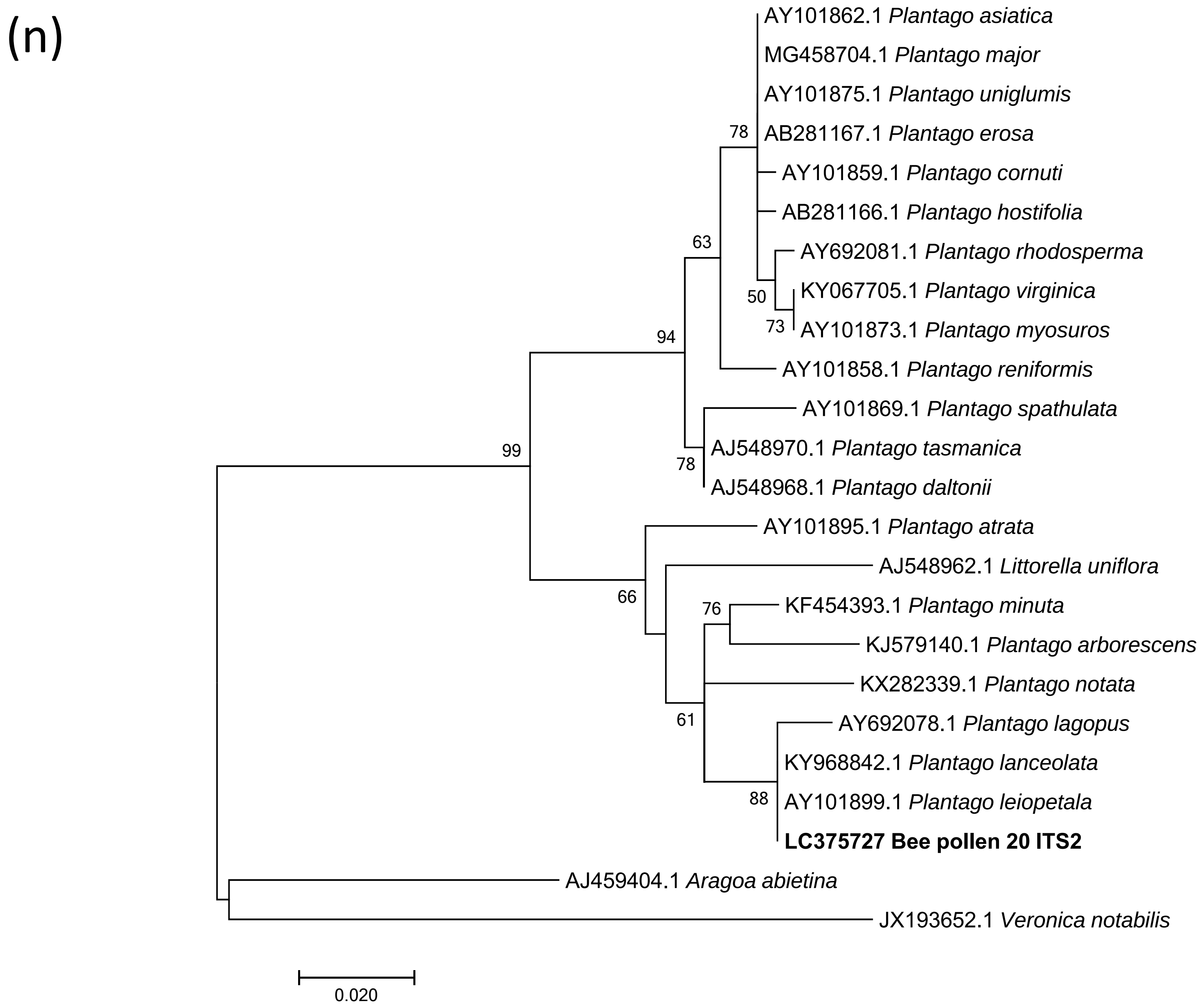
(n)
AY101862.1 Plantago asiatica
MG458704.1 Plantago major
AY101875.1 Plantago uniglumis
AB281167.1 Plantago erosa
AY101859.1 Plantago cornuti
AB281166.1 Plantago hostifolia
AY692081.1 Plantago rhodosperma
KY067705.1 Plantago virginica
AY101873.1 Plantago myosuros
AY101858.1 Plantago reniformis
AY101869.1 Plantago spathulata
AJ548970.1 Plantago tasmanica
AJ548968.1 Plantago daltonii
AY101895.1 Plantago atrata
AJ548962.1 Littorella uniflora
KF454393.1 Plantago minuta
KJ579140.1 Plantago arborescens
KX282339.1 Plantago notata
AY692078.1 Plantago lagopus
KY968842.1 Plantago lanceolata
AY101899.1 Plantago leiopetala
LC375727 Bee pollen 20 ITS2
AJ459404.1 Aragoa abietina
JX193652.1 Veronica notabilis
78
63
94
99
50
73
78
66
76
61
88
0.020

(o)

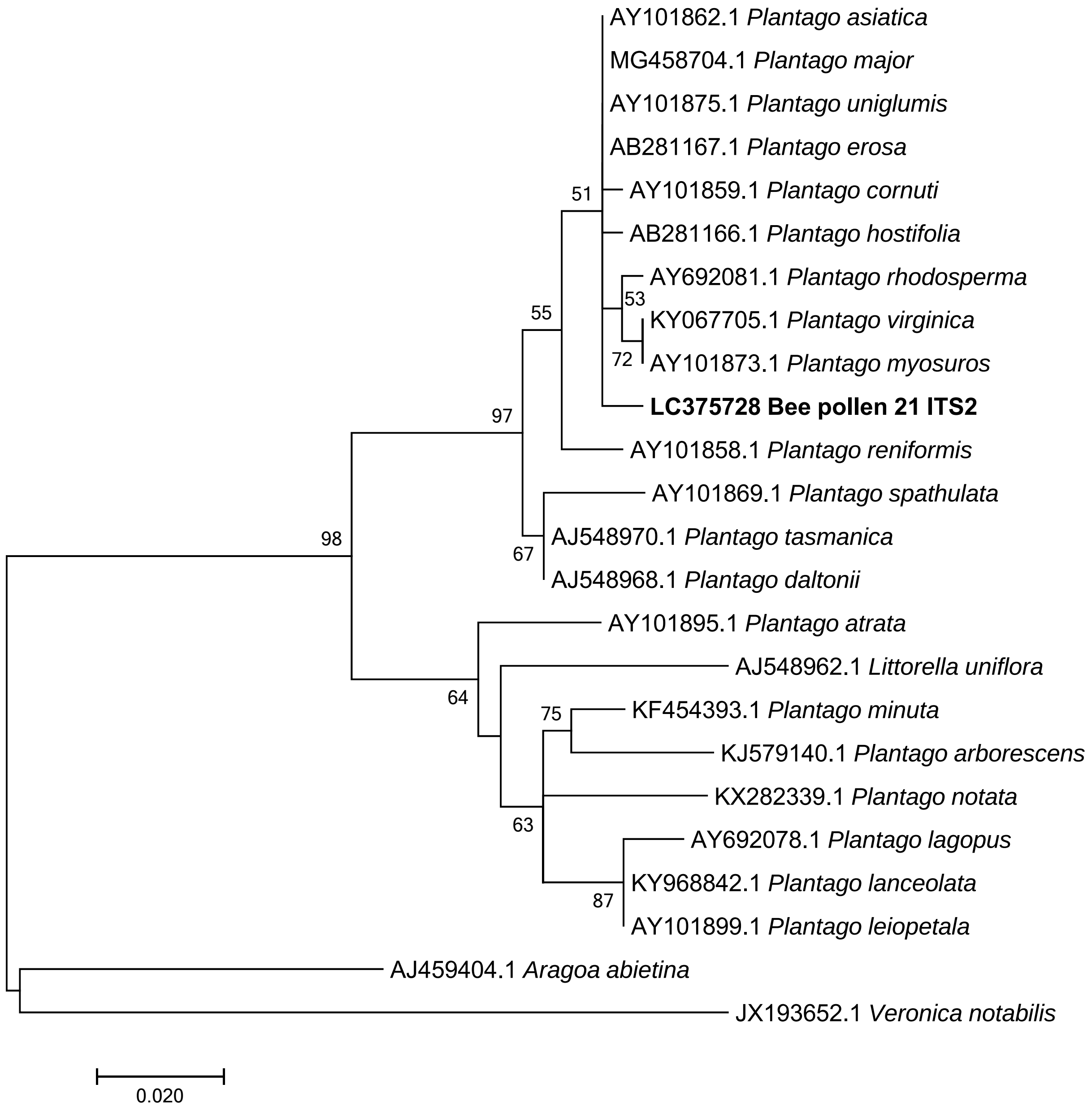

AY101862.1 Plantago asiatica
MG458704.1 Plantago major
AY101875.1 Plantago uniglumis
AB281167.1 Plantago erosa
AY101859.1 Plantago cornuti
AB281166.1 Plantago hostifolia
AY692081.1 Plantago rhodosperma
KY067705.1 Plantago virginica
AY101873.1 Plantago myosuros
LC375728 Bee pollen 21 ITS2
AY101858.1 Plantago reniformis
AY101869.1 Plantago spathulata
AJ548970.1 Plantago tasmanica
AJ548968.1 Plantago daltonii
AY101895.1 Plantago atrata
AJ548962.1 Littorella uniflora
KF454393.1 Plantago minuta
KJ579140.1 Plantago arborescens
KX282339.1 Plantago notata
AY692078.1 Plantago lagopus
KY968842.1 Plantago lanceolata
AY101899.1 Plantago leiopetala
AJ459404.1 Aragoa abietina
JX193652.1 Veronica notabilis
51
53
72
55
97
67
98
64
75
63
87
0.020

(p)

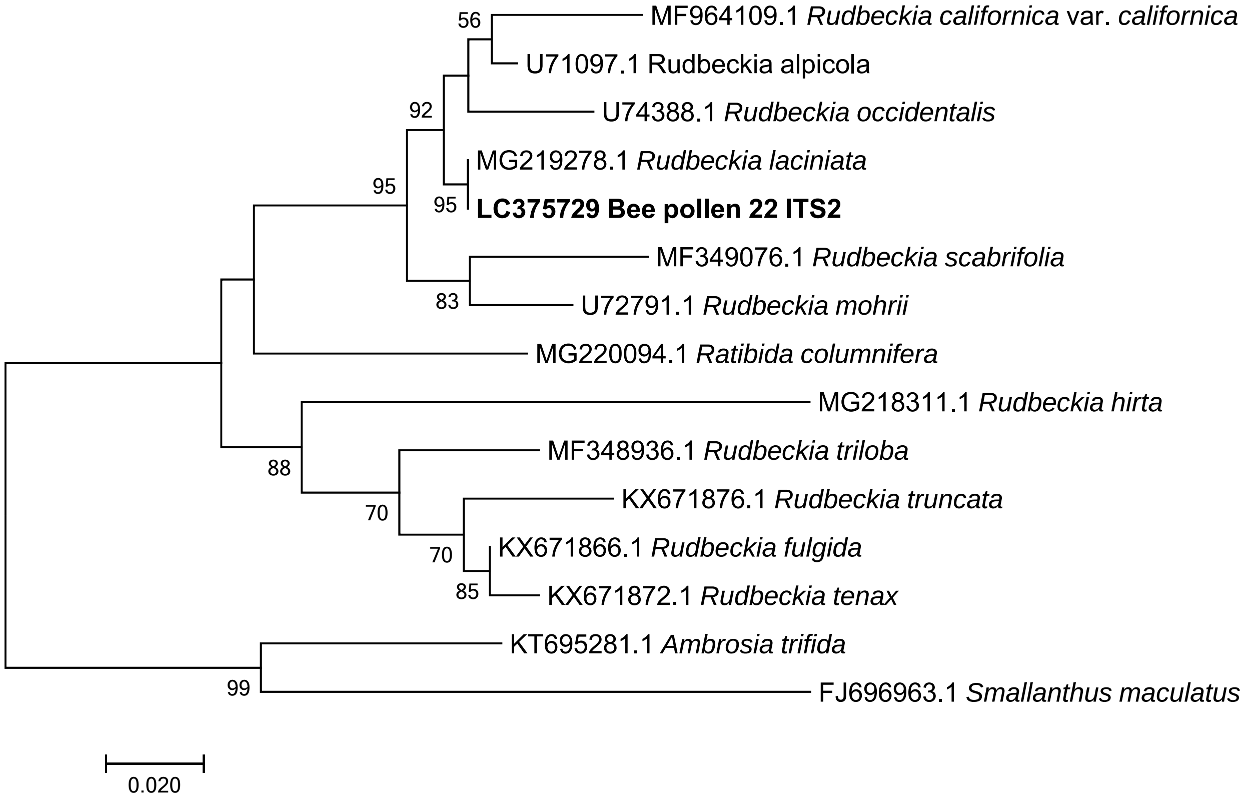
56
MF964109.1 Rudbeckia californica var. californica
U71097.1 Rudbeckia alpicola
92
U74388.1 Rudbeckia occidentalis
MG219278.1 Rudbeckia laciniata
95
95
LC375729 Bee pollen 22 ITS2
MF349076.1 Rudbeckia scabrifolia
83
U72791.1 Rudbeckia mohrii
MG220094.1 Ratibida columnifera
MG218311.1 Rudbeckia hirta
MF348936.1 Rudbeckia triloba
88
70
KX671876.1 Rudbeckia truncata
KX671866.1 Rudbeckia fulgida
70
85
KX671872.1 Rudbeckia tenax
KT695281.1 Ambrosia trifida
99
FJ696963.1 Smallanthus maculatus
0.020

(q)

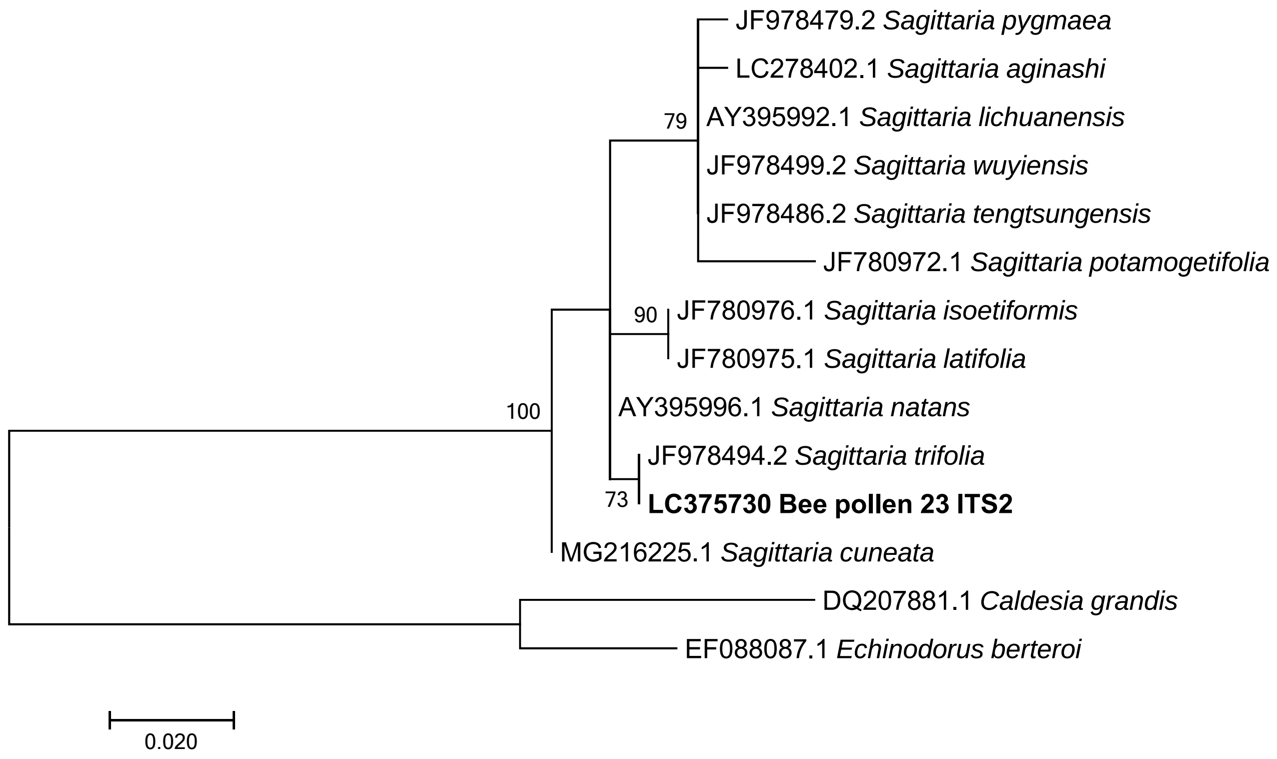
JF978479.2 Sagittaria pygmaea
LC278402.1 Sagittaria aginashi
AY395992.1 Sagittaria lichuanensis
JF978499.2 Sagittaria wuyiensis
JF978486.2 Sagittaria tengtsungensis
JF780972.1 Sagittaria potamogetifolia
JF780976.1 Sagittaria isoetiformis
JF780975.1 Sagittaria latifolia
AY395996.1 Sagittaria natans
JF978494.2 Sagittaria trifolia
LC375730 Bee pollen 23 ITS2
MG216225.1 Sagittaria cuneata
DQ207881.1 Caldesia grandis
EF088087.1 Echinodorus berteroi
79
90
100
73
0.020

(r)

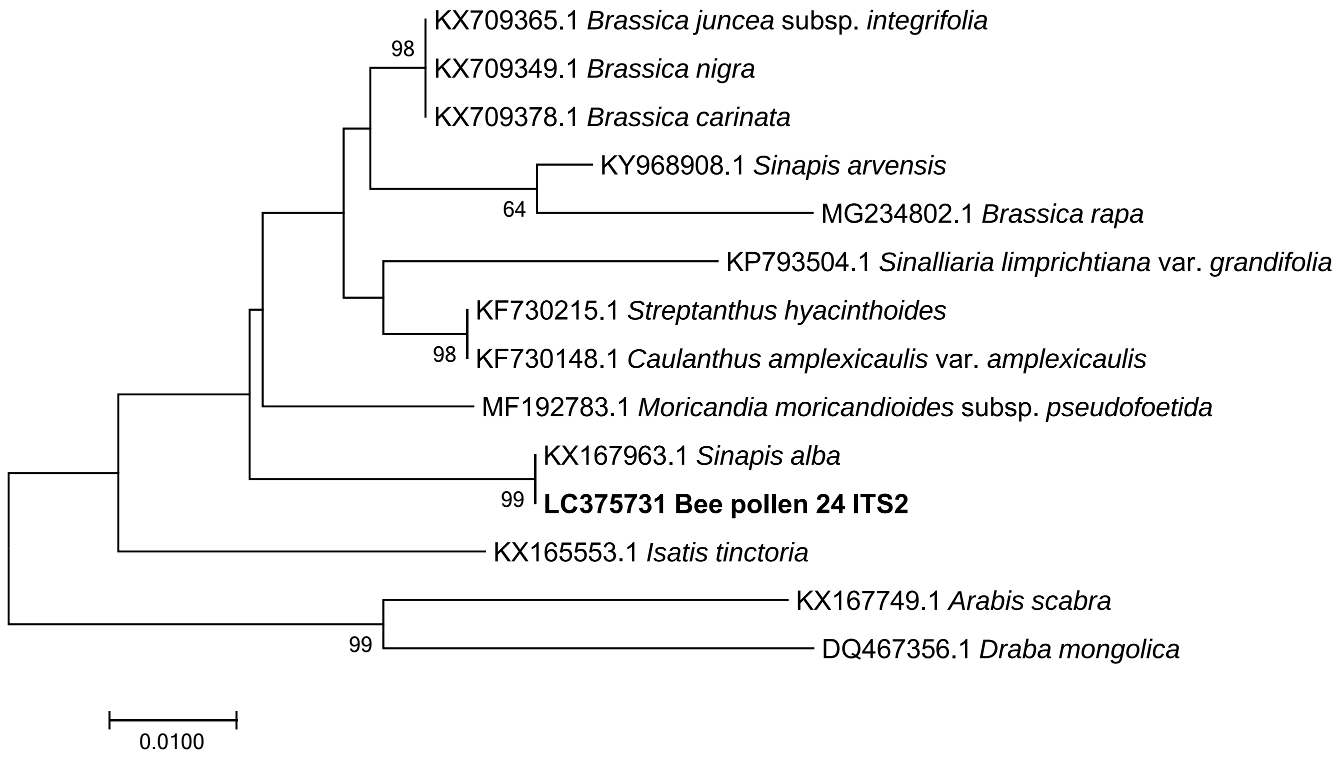
KX709365.1 Brassica juncea subsp. integrifolia
98
KX709349.1 Brassica nigra
KX709378.1 Brassica carinata
KY968908.1 Sinapis arvensis
64
MG234802.1 Brassica rapa
KP793504.1 Sinalliaria limprichtiana var. grandifolia
KF730215.1 Streptanthus hyacinthoides
98
KF730148.1 Caulanthus amplexicaulis var. amplexicaulis
MF192783.1 Moricandia moricandioides subsp. pseudofoetida
KX167963.1 Sinapis alba
99
LC375731 Bee pollen 24 ITS2
KX165553.1 Isatis tinctoria
KX167749.1 Arabis scabra
99
DQ467356.1 Draba mongolica
0.0100

(s)

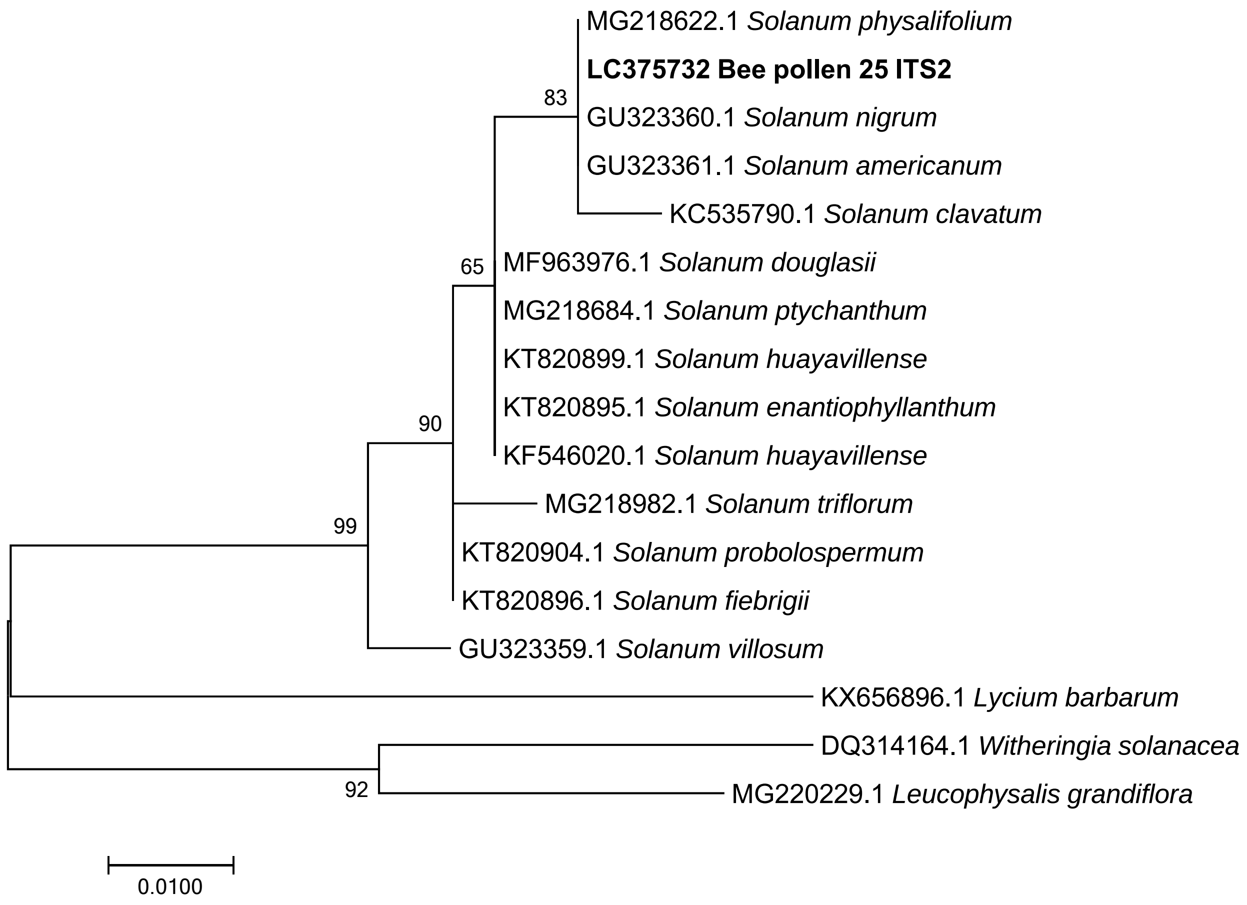
MG218622.1 *Solanum physalifolium*
**LC375732 Bee pollen 25 ITS2**
83
GU323360.1 *Solanum nigrum*
GU323361.1 *Solanum americanum*
KC535790.1 *Solanum clavatum*
65
MF963976.1 *Solanum douglasii*
MG218684.1 *Solanum ptychanthum*
KT820899.1 *Solanum huayavillense*
KT820895.1 *Solanum enantiophyllanthum*
90
KF546020.1 *Solanum huayavillense*
MG218982.1 *Solanum triflorum*
99
KT820904.1 *Solanum probolospermum*
KT820896.1 *Solanum fiebrigii*
GU323359.1 *Solanum villosum*
KX656896.1 *Lycium barbarum*
DQ314164.1 *Witheringia solanacea*
92
MG220229.1 *Leucophysalis grandiflora*
0.0100

(t)

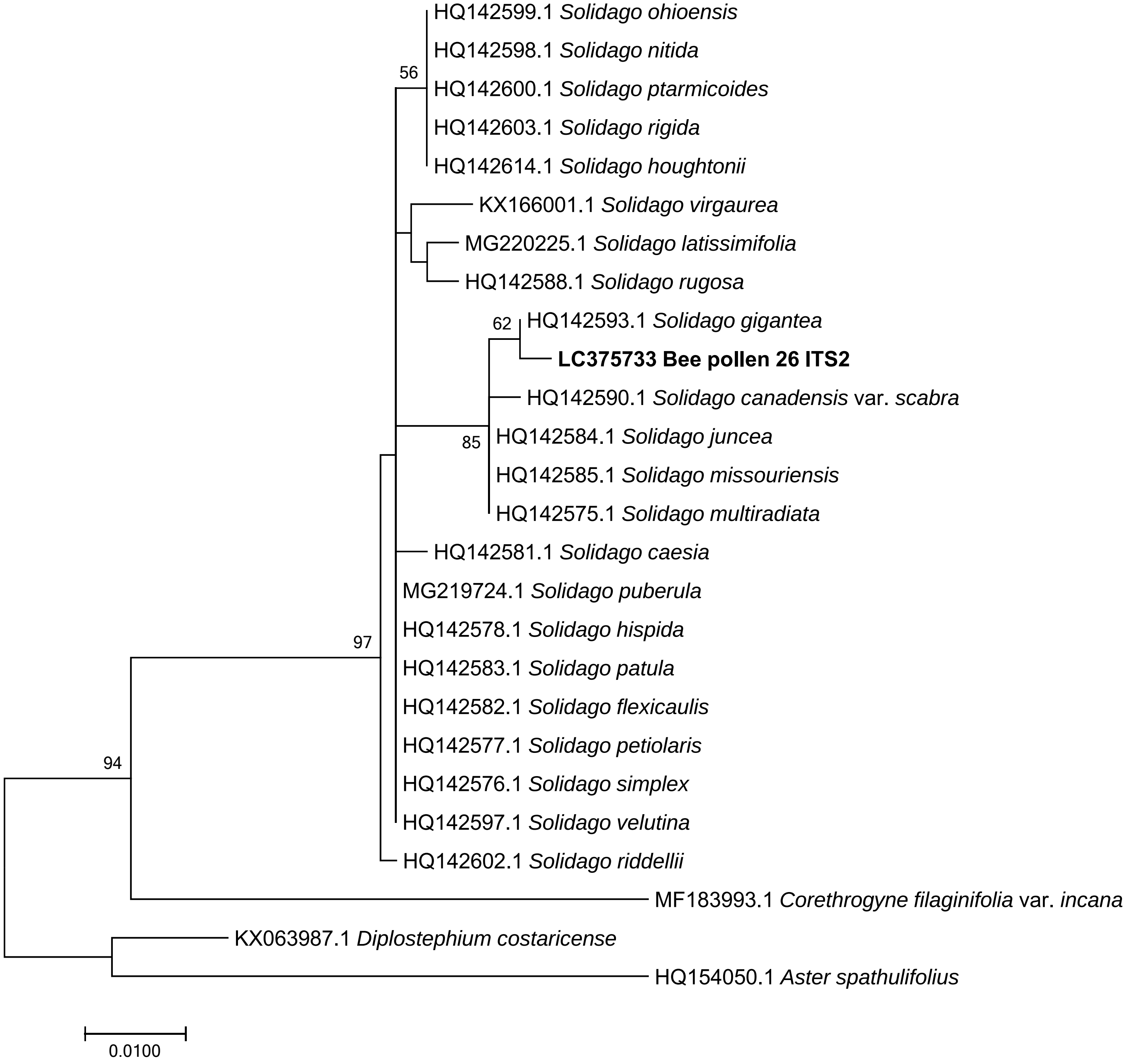

HQ142599.1 Solidago ohioensis
HQ142598.1 Solidago nitida
56
HQ142600.1 Solidago ptarmicoides
HQ142603.1 Solidago rigida
HQ142614.1 Solidago houghtonii
KX166001.1 Solidago virgaurea
MG220225.1 Solidago latissimifolia
HQ142588.1 Solidago rugosa
62
HQ142593.1 Solidago gigantea
LC375733 Bee pollen 26 ITS2
HQ142590.1 Solidago canadensis var. scabra
85
HQ142584.1 Solidago juncea
HQ142585.1 Solidago missouriensis
HQ142575.1 Solidago multiradiata
HQ142581.1 Solidago caesia
MG219724.1 Solidago puberula
97
HQ142578.1 Solidago hispida
HQ142583.1 Solidago patula
HQ142582.1 Solidago flexicaulis
HQ142577.1 Solidago petiolaris
94
HQ142576.1 Solidago simplex
HQ142597.1 Solidago velutina
HQ142602.1 Solidago riddellii
MF183993.1 Corethrogyne filaginifolia var. incana
KX063987.1 Diplostephium costaricense
HQ154050.1 Aster spathulifolius
0.0100

(u)

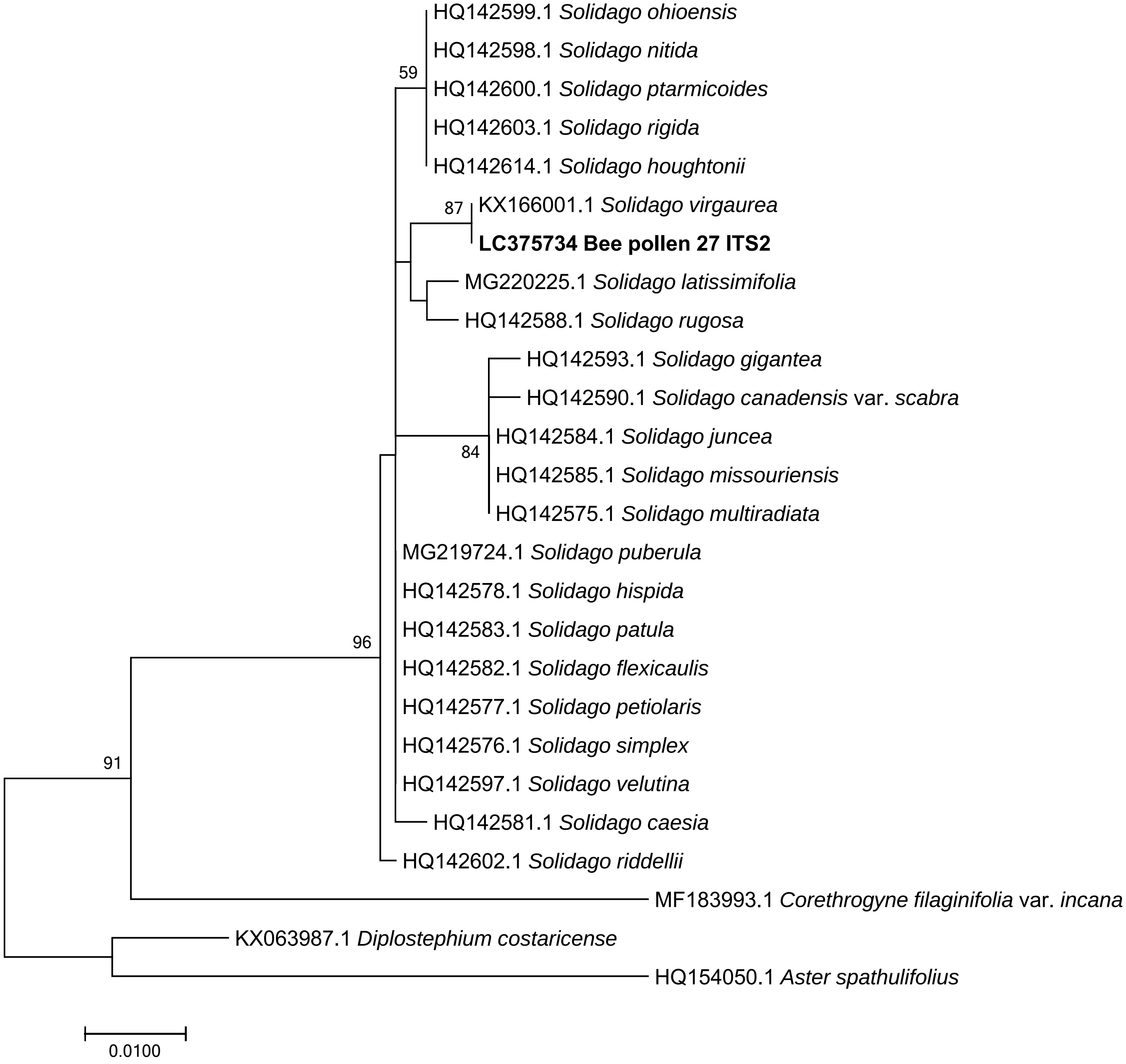
HQ142599.1 Solidago ohioensis
HQ142598.1 Solidago nitida
59
HQ142600.1 Solidago ptarmicoides
HQ142603.1 Solidago rigida
HQ142614.1 Solidago houghtonii
87
KX166001.1 Solidago virgaurea
LC375734 Bee pollen 27 ITS2
MG220225.1 Solidago latissimifolia
HQ142588.1 Solidago rugosa
HQ142593.1 Solidago gigantea
HQ142590.1 Solidago canadensis var. scabra
HQ142584.1 Solidago juncea
84
HQ142585.1 Solidago missouriensis
HQ142575.1 Solidago multiradiata
MG219724.1 Solidago puberula
HQ142578.1 Solidago hispida
HQ142583.1 Solidago patula
96
HQ142582.1 Solidago flexicaulis
HQ142577.1 Solidago petiolaris
HQ142576.1 Solidago simplex
91
HQ142597.1 Solidago velutina
HQ142581.1 Solidago caesia
HQ142602.1 Solidago riddellii
MF183993.1 Corethrogyne filaginifolia var. incana
KX063987.1 Diplostephium costaricense
HQ154050.1 Aster spathulifolius
0.0100

(v)

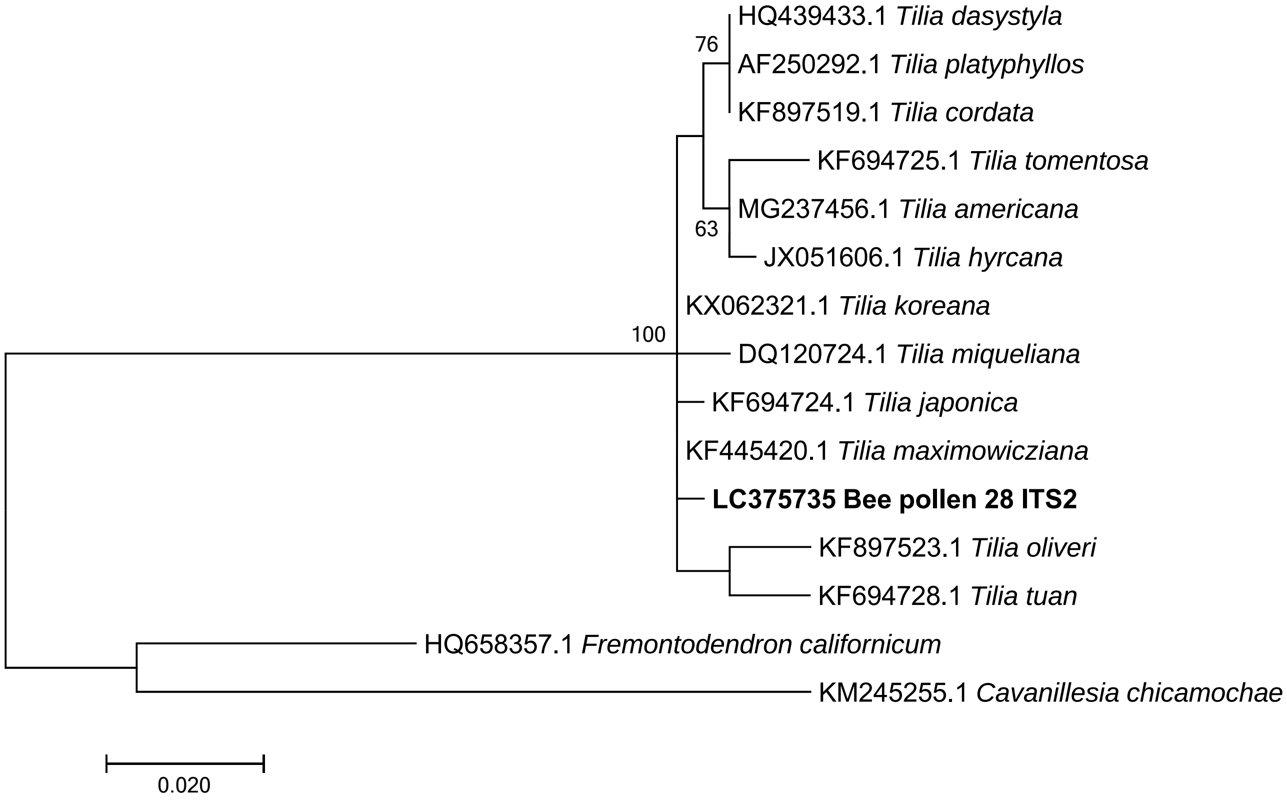
HQ439433.1 Tilia dasystyla
76
AF250292.1 Tilia platyphyllos
KF897519.1 Tilia cordata
KF694725.1 Tilia tomentosa
MG237456.1 Tilia americana
63
JX051606.1 Tilia hyrcana
KX062321.1 Tilia koreana
100
DQ120724.1 Tilia miqueliana
KF694724.1 Tilia japonica
KF445420.1 Tilia maximowicziana
LC375735 Bee pollen 28 ITS2
KF897523.1 Tilia oliveri
KF694728.1 Tilia tuan
HQ658357.1 Fremontodendron californicum
KM245255.1 Cavanillesia chicamochae
0.020

(w)

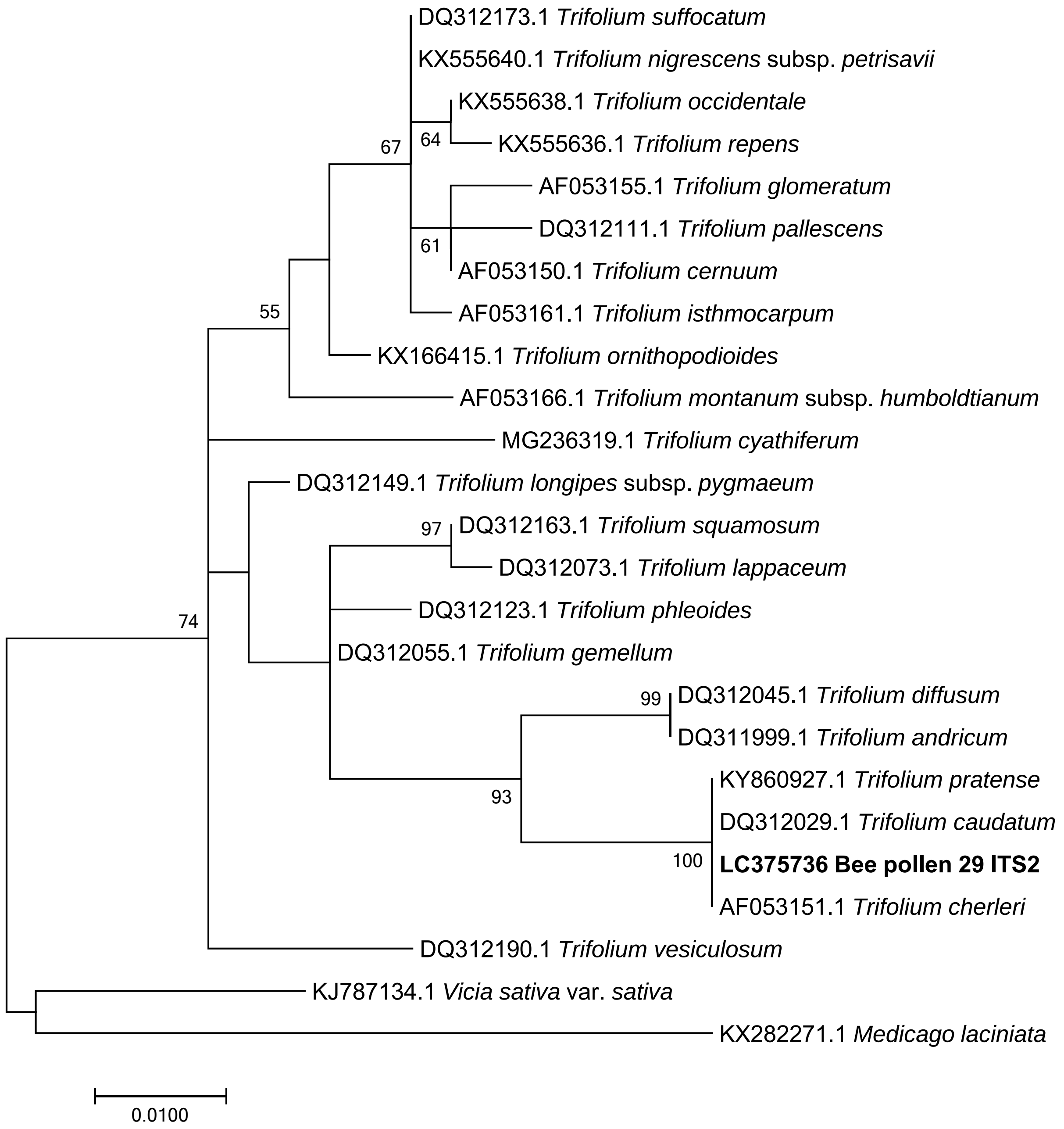
DQ312173.1 Trifolium suffocatum
KX555640.1 Trifolium nigrescens subsp. petrisavii
KX555638.1 Trifolium occidentale
KX555636.1 Trifolium repens
AF053155.1 Trifolium glomeratum
DQ312111.1 Trifolium pallescens
AF053150.1 Trifolium cernuum
AF053161.1 Trifolium isthmocarpum
KX166415.1 Trifolium ornithopodioides
AF053166.1 Trifolium montanum subsp. humboldtianum
MG236319.1 Trifolium cyathiferum
DQ312149.1 Trifolium longipes subsp. pygmaeum
DQ312163.1 Trifolium squamosum
DQ312073.1 Trifolium lappaceum
DQ312123.1 Trifolium phleoides
DQ312055.1 Trifolium gemellum
DQ312045.1 Trifolium diffusum
DQ311999.1 Trifolium andricum
KY860927.1 Trifolium pratense
DQ312029.1 Trifolium caudatum
LC375736 Bee pollen 29 ITS2
AF053151.1 Trifolium cherleri
DQ312190.1 Trifolium vesiculosum
KJ787134.1 Vicia sativa var. sativa
KX282271.1 Medicago laciniata
67
64
61
55
74
97
99
93
100
0.0100

(x)

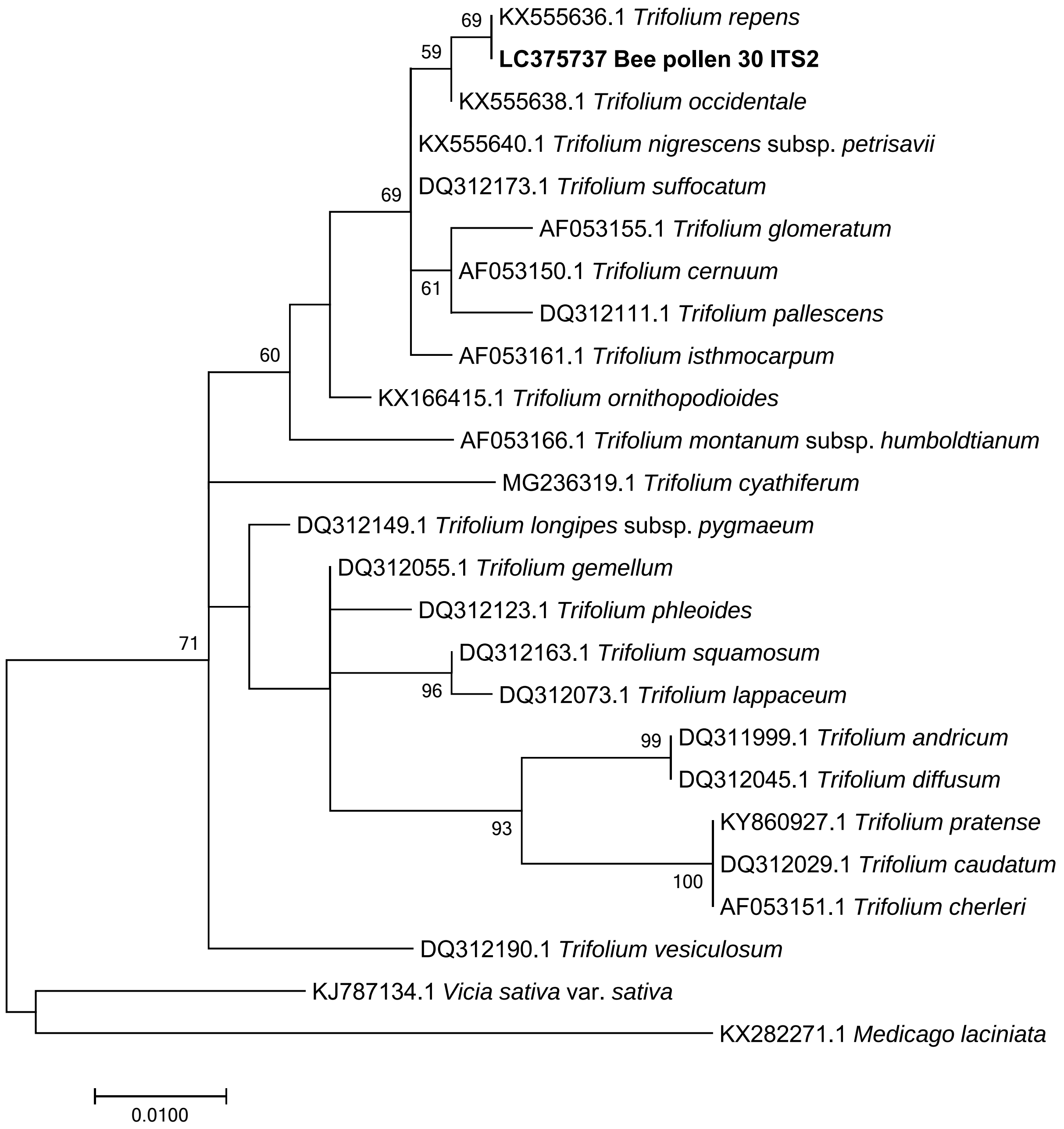
69
KX555636.1 Trifolium repens
59
LC375737 Bee pollen 30 ITS2
KX555638.1 Trifolium occidentale
KX555640.1 Trifolium nigrescens subsp. petrisavii
69
DQ312173.1 Trifolium suffocatum
AF053155.1 Trifolium glomeratum
AF053150.1 Trifolium cernuum
61
DQ312111.1 Trifolium pallescens
AF053161.1 Trifolium isthmocarpum
60
KX166415.1 Trifolium ornithopodioides
AF053166.1 Trifolium montanum subsp. humboldtianum
MG236319.1 Trifolium cyathiferum
DQ312149.1 Trifolium longipes subsp. pygmaeum
DQ312055.1 Trifolium gemellum
DQ312123.1 Trifolium phleoides
71
DQ312163.1 Trifolium squamosum
96
DQ312073.1 Trifolium lappaceum
99
DQ311999.1 Trifolium andricum
DQ312045.1 Trifolium diffusum
KY860927.1 Trifolium pratense
93
DQ312029.1 Trifolium caudatum
100
AF053151.1 Trifolium cherleri
DQ312190.1 Trifolium vesiculosum
KJ787134.1 Vicia sativa var. sativa
KX282271.1 Medicago laciniata
0.0100
