# Supplementary material for: A DNA barcoding method for identifying and quantifying the composition of pollen species collected by European honeybees, Apis mellifera (Hymenoptera: Apidae)
Source: Appl Entomol Zool. 2018 May 16;53(3):353–61. doi: 10.1007/s13355-018-0565-9 (PMC6060998; doi:10.1007/s13355-018-0565-9)
Supplement: Supplementary file 7 — Supplementary material 7 (PDF 125 kb) [file 13355_2018_565_MOESM7_ESM.pdf]

Fig. S3 Molecular phylogenetic tree of honeybee pollen pellet by maximum likelihood method in *psbA-trnH*

The evolutionary history was inferred by using the maximum likelihood method based on the Tamura–Nei model. The tree with the highest log likelihood (−730.8411) is shown. The percentage of trees in which the associated taxa clustered together is shown next to the branches. Initial tree(s) for the heuristic search were obtained automatically by applying the Neighbor-Join and BioNJ algorithms to a matrix of pairwise distances estimated using the maximum composite likelihood approach, and then selecting the topology with superior log likelihood value. The tree is drawn to scale, with branch lengths indicating the number of substitutions per site. The analysis involved 14 nucleotide sequences. All positions containing gaps and missing data were eliminated. There were a total of 370 positions in the final dataset. Evolutionary analyses were conducted in MEGA7.

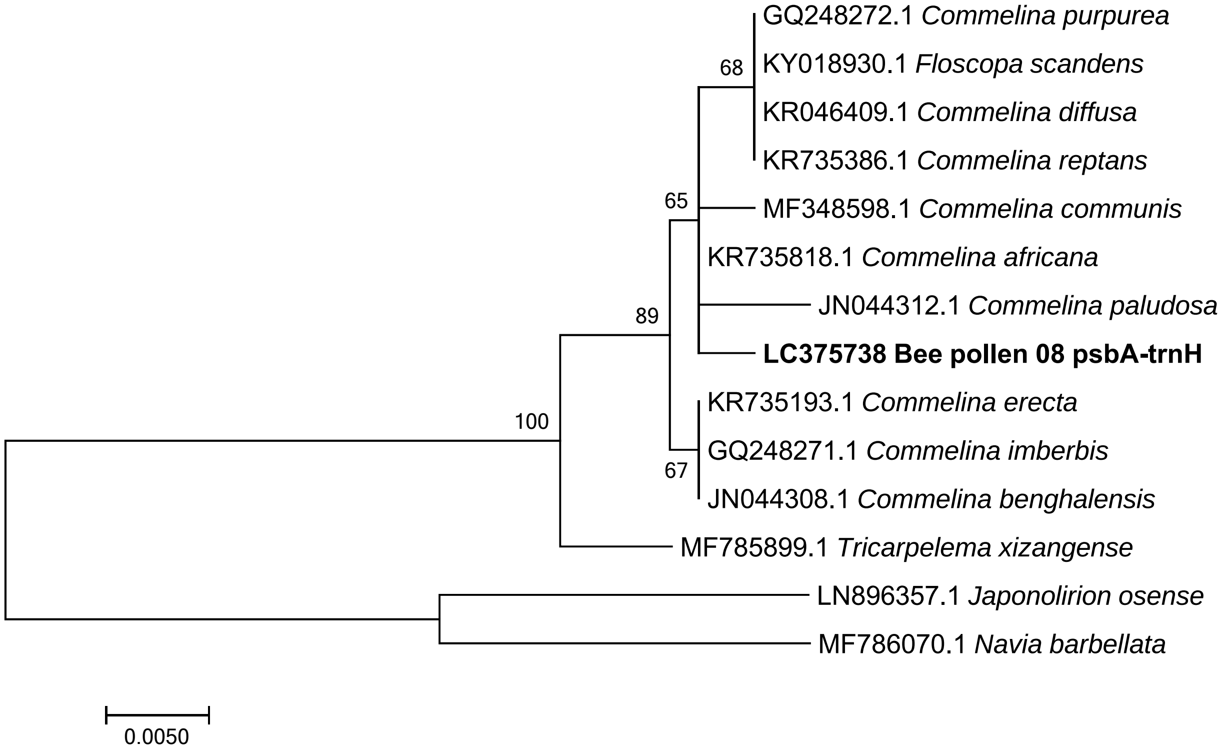
GQ248272.1 Commelina purpurea
KY018930.1 Floscopa scandens
KR046409.1 Commelina diffusa
KR735386.1 Commelina reptans
MF348598.1 Commelina communis
KR735818.1 Commelina africana
JN044312.1 Commelina paludosa
LC375738 Bee pollen 08 psbA-trnH
KR735193.1 Commelina erecta
GQ248271.1 Commelina imberbis
JN044308.1 Commelina benghalensis
MF785899.1 Tricarpelema xizangense
LN896357.1 Japonolirion osense
MF786070.1 Navia barbellata
68
65
89
100
67
0.0050
