# Supplementary material for: A DNA barcoding method for identifying and quantifying the composition of pollen species collected by European honeybees, Apis mellifera (Hymenoptera: Apidae)
Source: Appl Entomol Zool. 2018 May 16;53(3):353–61. doi: 10.1007/s13355-018-0565-9 (PMC6060998; doi:10.1007/s13355-018-0565-9)
Supplement: Supplementary file 8 — Supplementary material 8 (PDF 124 kb) [file 13355_2018_565_MOESM8_ESM.pdf]

Fig. S4 Molecular phylogenetic tree of honeybee pollen pellet by maximum likelihood method in *matK*

The evolutionary history was inferred by using the maximum likelihood method based on the Tamura–Nei model. The tree with the highest log likelihood (−1345.0433) is shown. The percentage of trees in which the associated taxa clustered together is shown next to the branches. Initial tree(s) for the heuristic search were obtained automatically by applying the Neighbor-Join and BioNJ algorithms to a matrix of pairwise distances estimated using the maximum composite likelihood approach, and then selecting the topology with superior log likelihood value. The tree is drawn to scale, with branch lengths indicating the number of substitutions per site. The analysis involved 10 nucleotide sequences. Codon positions included were 1st + 2nd + 3rd + noncoding. All positions containing gaps and missing data were eliminated. There were a total of 641 positions in the final dataset. Evolutionary analyses were conducted in MEGA7.

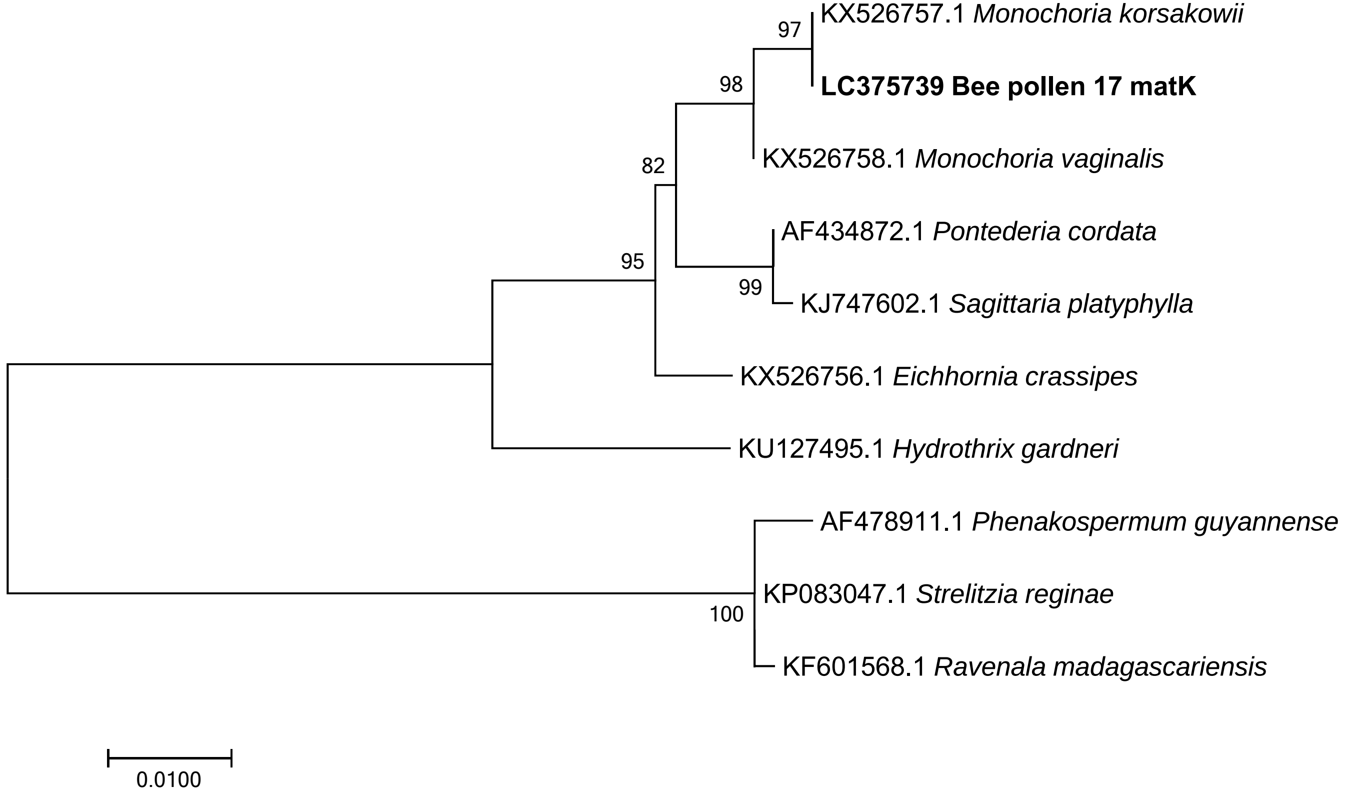
KX526757.1 Monochoria korsakowii
97
98
LC375739 Bee pollen 17 matK
KX526758.1 Monochoria vaginalis
82
AF434872.1 Pontederia cordata
95
99
KJ747602.1 Sagittaria platyphylla
KX526756.1 Eichhornia crassipes
KU127495.1 Hydrothrix gardneri
AF478911.1 Phenakospermum guyannense
KP083047.1 Strelitzia reginae
100
KF601568.1 Ravenala madagascariensis
0.0100
